# Synthesis and Biological Evaluation of New Madecassic Acid Derivatives Targeting ERK Cascade Signaling

**DOI:** 10.3389/fchem.2018.00434

**Published:** 2018-09-28

**Authors:** Ana S. C. Valdeira, Daniel A. Ritt, Deborah K. Morrison, James B. McMahon, Kirk R. Gustafson, Jorge A. R. Salvador

**Affiliations:** ^1^Laboratory of Pharmaceutical Chemistry, Faculty of Pharmacy, University of Coimbra, Coimbra, Portugal; ^2^Center for Neuroscience and Cell Biology, University of Coimbra, Coimbra, Portugal; ^3^Laboratory of Cell and Developmental Signaling, Center for Cancer Research, National Cancer Institute, Frederick, MD, United States; ^4^Molecular Targets Program, Center for Cancer Research, National Cancer Institute, Frederick, MD, United States

**Keywords:** madecassic acid, synthesis, derivatives, NCI-60 cell line screening, B-Raf^V600E^ mutation, ERK cascade, anticancer activity

## Abstract

In the present study, a series of novel madecassic acid derivatives was synthesized and screened against the National Cancer Institute's 60 human cancer cell line panel. Among them, compounds **5**, **12**, and **17** displayed potent and highly differential antiproliferative activity against 80% of the tumor cells harboring the B-Raf^V600E^ mutation within the nanomolar range. Structure-activity analysis revealed that a 5-membered A ring containing an α,β-unsaturated aldehyde substituted at C-23 with a 2-furoyl group seems to be crucial to produce this particular growth inhibition signature. *In silico* analysis of the cytotoxicity pattern of these compounds identified two highly correlated clinically approved drugs with known B-Raf^V600E^ inhibitory activity. Follow-up analysis revealed inhibition of the ERK signaling pathway through the reduction of cellular Raf protein levels is a key mechanism of action of these compounds. In particular, **17** was the most potent compound in suppressing tumor growth of B-Raf^V600E^-mutant cell lines and displayed the highest reduction of Raf protein levels among the tested compounds. Taken together, this study revealed that modifications of madecassic acid structure can provide molecules with potent anticancer activity against cell lines harboring the clinically relevant B-Raf^V600E^ mutation, with compound **17** identified as a promising lead for the development of new anticancer drugs.

## Introduction

The search for novel anticancer agents from natural sources continues to be a productive strategy for the identification of new clinical candidates (Shah et al., 2013; Bandyopadhyay, 2014; Rayan et al., 2017). Plants, in particular, have been a prime source of bioactive small-molecules which tend to present more structurally diverse “drug-like” and “biologically friendly” molecular qualities than most synthetic compounds, thus making them important sources of novel lead structures for anticancer drug discovery (Vuorelaa et al., 2004; Pan et al., 2010; Basmadjian et al., 2014). Triterpenoids have emerged as a prominent group of plant-derived small molecules with multifunctional anticancer activities, as demonstrated by promising results in preclinical studies (Bishayee et al., 2011; Salvador et al., 2012, 2017; Wang et al., 2014; Figueiredo et al., 2017). In the past decades, numerous reports have described the cellular and molecular mechanism(s) underlying the anticancer activity of triterpenoids. Among the most relevant mechanisms involved are cell cycle arrest, apoptosis and autophagy triggered by the effect of these secondary metabolites on the mitogen-activated protein kinase (MAPK) (Konopleva et al., 2005), phosphatidylinositol 3-kinase/Akt/mammalian target of rapamycin (PI3K-Akt-mTOR) (Yore et al., 2011), signal transducer and activator of transcription 3 (STAT3) (Fitzpatrick et al., 2014) and nuclear factor kappa B (NF-κB) (Patil et al., 2015) signaling pathways.

**Figure 1:**
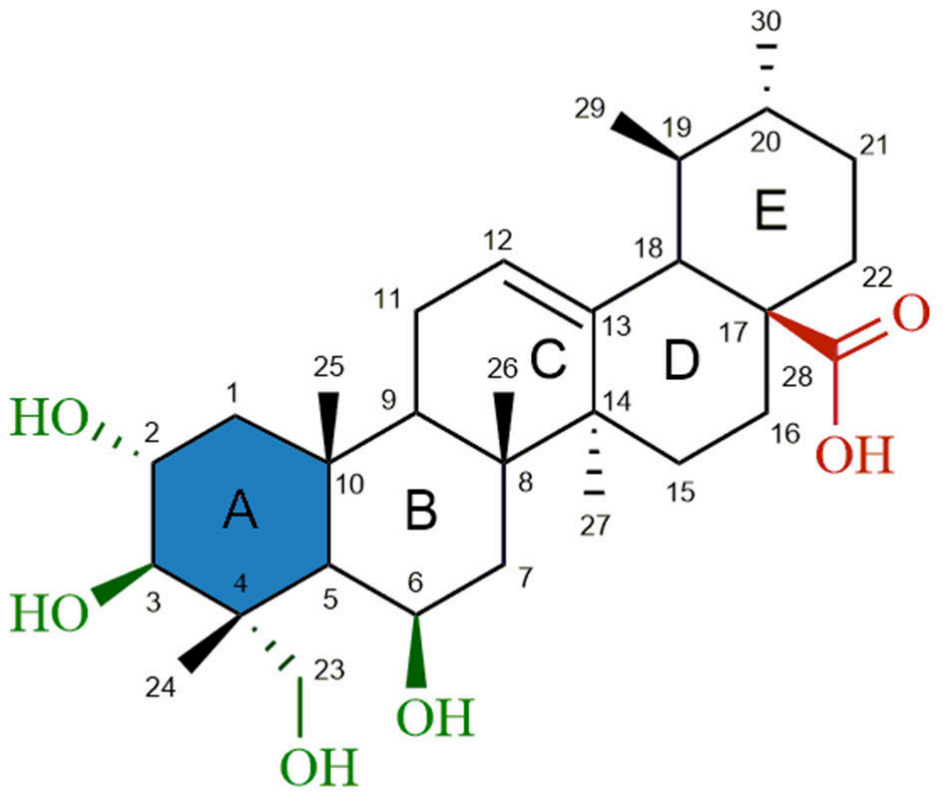
Chemical structure of madecassic acid (**MEA**, **1**). The C-2, C-3, C-6 and C-23 hydroxyl groups (green), the C-28 carboxylic acid group (red) and the A-ring (blue) were the regions targeted for semi-synthetic modification.

Madecassic acid [**MEA** (**1**), Figure 1] is a major triterpenoid carboxylic acid present in *Centella asiatica* (James and Dubery, 2009) and has been shown to possess several attractive pharmacological activities, such as wound healing (Bonte et al., 1994), antioxidant (Yin et al., 2012; Yang et al., 2016), anti-inflammatory (Won et al., 2010) and antidiabetic (Hsu et al., 2015) activities. Furthermore, a recent study (Zhang et al., 2014) gave evidence for an apoptotic effect of MEA in an *in vivo* model using mice bearing CT26 cancer cells. Although this study did not comprehensively explore the mechanism by which cancer cell apoptosis was induced by madecassic acid, immunostaining experiments suggested that madecassic acid treatment decreased the mitochondrial membrane potential, which contributed to the cancer cell apoptosis. A significant increase of CD4^+^ and CD8^+^ T-lymphocyte subpopulations, as well as an increased secretion of IFN-γ and IL-4, was also observed after madecassic acid administration, suggesting that this compound might also play an important role in cancer immunotherapy. Despite its promising biological and pharmaceutical activities, low toxicity and commercial availability, only a few studies have attempted to explore the therapeutic potential of MEA. Furthermore, as compared to other triterpenoids, only a very limited number of derivatives of MEA have been reported and tested for antitumor activity.

Modifications of the A ring account for the vast majority of the semi-synthetic ursane-type triterpenoid derivatives reported in the literature. A particularly successful modification was reported for the first time in 1969 by Sign et al. (Singh and Rastogi, 1969) which resulted in the conversion of the 6-membered A ring of the asiatic acid (AA) into a 5-membered ring containing an α,β-unsaturated carbonyl group. Since then several studies have identified this promising electrophilic Michael acceptor as an important chemical feature that significantly enhances the cytotoxic proprieties of triterpenoids while retaining their ability to induce apoptosis (Sporn et al., 2011; Salvador et al., 2012; Goncalves et al., 2016).

In the light of the above mentioned facts, and in continuation of our ongoing research program which aimed at the design and synthesis of new semi-synthetic madecassic acid derivatives as anticancer agents, we sought to develop a series of new MEA derivatives containing a 5-carbon ring A with an α,β-unsaturated carbonyl moiety, combined with additional modifications at C-6, C-23, and C-28, to obtain MEA (**1**) derivatives with improved anticancer activity.

The antitumor activities of MEA and 14 of these novel semi-synthetic compounds were assessed using a panel of 60 tumor cell lines at the National Cancer Institute (NCI) and a structure-activity relationship (SAR) was established. The NCI antitumor screening identified three highly cytotoxic derivatives (**5**, **12**, and **17**) with remarkable selectivity toward B-Raf^V600E^-mutant cell lines. The molecular mechanisms underlying the anticancer activity of these promising compounds were predicted using the web-based application CellMiner^TM^. CellMiner^TM^ analysis revealed that the mechanism of action (MOA) of these MEA derivatives may involve B-Raf or other elements of the ERK kinase cascade. *In vitro* studies were performed in B-Raf^V600E^ colon (Colo205) and melanoma (Malme-3M and SK-Mel-28) cell lines to confirm this prediction.

## Materials and methods

### Chemistry

#### General

Madecassic acid (MEA) was purchased from Santa Cruz Biotechnology Inc., in over 95% purity. Other reagents and solvents were purchased from Sigma-Aldrich Co., Merck Co. and VWR Portugal and used without further purification. Solvents were dried over standard drying agents according to usual procedures. Thin layer chromatography (TLC) analysis and preparative TLC were carried out in Kieselgel 60HF254 and Kieselgel 60HF254/Kieselgel 60G from Merck Co., respectively. Column chromatographic separations were performed using Kieselgel 60 (230–400 mesh) from Merck Co. Melting points were determined by using open capillary tubes on a BÜCHI B-540 melting point apparatus and are uncorrected. ^1^H, ^13^C, DEPT-135, HSQC, and HMBC NMR experiments were performed in CDCl_3_ or C_6_D_6_ and recorded on a Bruker Avance 400 and Bruker Avance III spectrometers operating at 400 MHz and 600 MHz for ^1^H and 100 MHz and 150 MHz for ^13^C, respectively. The Bruker Avance III NMR spectrometer was equipped with a 3 mm cryogenically cooled probe. Spectra were calibrated to residual solvent signals at δ_H_ 7.26 and δ_C_ 77.16 (CDCl_3_) and δ_H_ 7.16 and δ_C_ 128.06 (C_6_D_6_). Chemical shifts (δ) were expressed in parts per million (ppm) and the coupling constants (*J*) in Hertz (Hz). The following abbreviations were used in reporting spectra: s = singlet, br s = broad singlet, d = doublet, dd = double doublet, t = triplet, td = triple doublet, q = quartet, m = multiple. IR spectra were recorded on a Perkin Elmer Spectrum 2000 FT-IR spectrometer spectrophotometer using NaCl circular cell windows. Low-resolution ESI-MS spectra were recorded on a Linear Ion Trap Mass Spectrometer (LTQ XL, THERMO). High-resolution ESI-MS spectra were performed with an Agilent 6530B Accurate Mass Q-TOF mass spectrometer and presented as *m*/*z*. The purity of compounds was determined to be greater than 95% based on LC-MS analysis.

#### Synthesis and structural characterization of compounds 2-17

##### Methyl 2α,3β,6β,23-tetrahydroxyurs-12-en-28-oate (2)

To a stirred solution of **1** (400 mg, 0.79 mmol) and anhydrous potassium carbonate (274 mg, 1.98 mmol) in DMF (8 mL), methyl iodide (0.1 mL, 1.6 mmol) was added. After 1 h at room temperature the reaction was completed (monitored by TLC). The reaction mixture was evaporated under reduced pressure, the obtained residue diluted with water (100 mL) and extracted with diethyl ether (3 × 100 mL). The combined organic layers were washed with 10% aqueous Na_2_SO_3_ (3 × 50 mL), water (3 × 50 mL), brine (50 mL) and dried over anhydrous magnesium sulfate. Filtration and evaporation of the solvent at reduced pressure gave a crude solid, which was subjected to flash column chromatography with an isocratic elution of petroleum ether (40–65°C)/EtOAc 1:25 (v/v) to afford **2** as a white solid (338 mg, 82%). Mp: 177.3–179.1°C. IR *v*_max_ (NaCl): 3358, 3020, 2922, 2852, 1742, 1634, 1463, 1242, 1168 cm^−1^. ^1^H NMR (600 MHz, CDCl_3_): δ_H_ 5.30 (1H, br t, *J* = 3.5 Hz, H-12), 4.41 (1H, br s, H-6), 3.84 (1H, dt, *J* = 10, 4.4 Hz, H-2), 3.75 and 3.54 (each 1H, d, *J* = 11 Hz, H-23), 3.60 (3H, s, COOCH_3_), 3.37 (1H, d, *J* = 9.5 Hz, H-3), 2.26 (1H, d, *J* = 12 Hz, H-18), 2.08 and 2.01 (each 1H, m, H-11), 2.00 (1H, m, H-16a), 1.99 (1H, m, H-1a), 1.82 (1H, m, H-15a), 1.78 (1H, m, H-7a), 1.68 (1H, m, H-16b), 1.67 (1H, m, H-22a), 1.66 (1H, m, H-9), 1.59 (1H, m, H-22b), 1.50 (1H, m, H-21), 1.47 (1H, m, H-7b), 1.40 (3H, s, H-25), 1.33 (1H, m, H-19), 1.28 (1H, m, H-21b), 1.21 (3H, s, H-24), 1.19 (1H, m, H-5), 1.06 (1H, m, H-15b), 1.05 (3H, s, H-27), 1.03 (3H, s, H-26), 1.01 (1H, m, H-20), 0.96 (1H, m, H-1b), 0.95 (3H, d, *J* = 6.5 Hz, H-30), 0.87 (3H, d, *J* = 6.5 Hz, H-29). ^13^C NMR (150 MHz, CDCl_3_): δ_C_ 178.2 (COOCH_3_, C-28), 137.6 (C-13), 125.7 (C-12), 79.0 (C-3), 69.0 (C-2), 68.4 (C-23), 68.0 (C-6), 53.0 (C-18), 51.7 (COO*C*H_3_), 49.0 (C-5), 48.9 (C-9), 48.7 (C-17), 48.2 (C-1), 43.4 (C-4), 42.7 (C-14), 40.9 (C-7), 39.0 (C-19), 38.7 (C-10 and C-20), 37.7 (C-8), 36.8 (C-22), 30.8 (C-21), 28.1 (C-15), 24.3 (C-16), 23.9 (C-27), 23.4 (C-11), 21.3 (C-30), 18.9 (C-25), 18.5 (C-26), 17.2 (C-29), 14.6 (C-24). MS (LIT) *m/z* [M+Na]^+^: 541.36; HRMS (Q-TOF) *m*/*z* [M + Na]^+^ calculated for C_31_H_50_O_6_Na = 541.3505, found = 541.3508 (Δ = 0.55 ppm).

##### Methyl 2-formyl-6β,23-dihydroxy-A(1)-norursa-2,12-dien-28-oate (3)

To a solution of **2** (400 mg, 0.77 mmol) in methanol/water (7.8 mL/0.4 mL, 20:1), sodium periodate (249.81 mg, 1.17 mmol) was added. After 2 h at room temperature the reaction was completed (monitored by TLC). The reaction mixture was evaporated under reduced pressure, the obtained residue diluted with water (100 mL) and extracted with diethyl ether (3 × 100 mL). The combined organic layers were washed with water (3 × 100 mL) and brine (100 mL). The organic phase was dried over anhydrous magnesium sulfate. Filtration and evaporation of the solvent at reduced pressure gave a crude solid. Dry benzene (24 mL), piperidine (2.1 mL) and glacial acetic acid (2.1 mL) were added and the reaction mixture was heated at 60°C for 1 h under nitrogen atmosphere. Anhydrous magnesium sulfate (400 mg, 3.32 mmol) was then added and the reaction continued for another 2 h. The reaction mixture was evaporated under reduced pressure, the obtained residue diluted with water (100 mL) and extracted with diethyl ether (3 × 100 mL). The combined organic layers were washed with water (3 × 100 mL) and brine (100 mL). The organic phase was dried over anhydrous magnesium sulfate. Filtration and evaporation of the solvent at reduced pressure gave a crude solid, which was subjected to flash column chromatography with a gradient elution of petroleum ether (40–65°C)/EtOAc from 5:1 to 2:1 (v/v) to afford **3** as a white solid (294 mg, 77%). Mp: 216.3–218.1°C. IR *v*_max_ (NaCl): 3501, 3067, 2924, 2870, 2737, 1733, 1684, 1456, 1379, 1273, 1195 cm^−1^. ^1^H NMR (600 MHz, CDCl_3_): δ_H_ 9.71 (1H, s, CHO), 6.61 (1H, s, H-3), 5.33 (1H, br t, *J* = 3.6 Hz, H-12), 4.51 (1H, m, H-6), 3.68 and 3.51 (each 1H, d, *J* = 11 Hz, H-23), 3.61 (3H, s, COOCH_3_), 2.54 and 2.37 (each 1H, m, H-11), 2.25 (1H, d, *J* = 11 Hz, H-18), 2.19 (1H, m, H-9), 1.99 (1H, m, H-16a), 1.89 (1H, br s, H-5), 1.87 (1H, m, H-15a), 1.85 (1H, m, H-7a), 1.68 (1H, m, H-16b), 1.64 (1H, m, H-22a), 1.61 (3H, s, H-25), 1.60 (1H, m, H-22b), 1.54 (1H, m, H-7b), 1.49 (1H, m, H-21a) 1.30 (3H, s, H-24), 1.29 (1H, m, H-19), 1.28 (1H, m, H-21b), 1.15 (3H, s, H-26), 1.06 (3H, s, H-27), 1.02 (1H, m, H-15b), 1.01 (1H, m, H-20), 0.94 (3H, d, *J* = 6.3 Hz, H-30), 0.85 (3H, d, *J* = 6.4 Hz, H-29). ^13^C NMR (150 MHz, CDCl_3_): δ_C_ 190.8 (*C*HO), 178.2 (*C*OOCH_3_, C-28), 158.7 (C-3), 158.6 (C-2), 137.3 (C-13), 126.7 (C-12), 69.3 (C-23), 68.2 (C-6), 56.2 (C-5), 53.0 (C-18), 51.7 (COO*C*H_3_), 50.8 (C-10), 49.7 (C-4), 48.2 (C-17), 44.5 (C-9), 43.0 (C-14), 41.8 (C-7), 41.2 (C-8), 39.0 (C-20), 38.9 (C-19), 36.7 (C-22), 30.8 (C-21), 28.4 (C-15), 27.2 (C-11), 24.3 (C-16), 24.2 (C-27), 21.3 (C-30), 21.1 (C-25), 20.2 (C-26), 17.5 (C-24), 17.2 (C-29). MS (LIT) *m/z* [M + Na]^+^: 521.38; HRMS (Q-TOF) *m*/*z* [M + H]^+^ calculated for C_31_H_47_O_5_ = 499.3423, found = 499.3424 (Δ = 0.20 ppm).

##### Methyl 2-formyl-6β-hydroxy-23-acetyloxy-A(1)-norursa-2,12-dien-28-oate (4)

To a solution of **3** (150 mg, 0.30 mmol) in dry THF (1.5 mL), acetic anhydride (0.09 mL, 0.9 mmol, 3 eq.) and a catalytic amount of DMAP (15 mg, 0.12 mmol) were added. After 2 h at room temperature the reaction was completed (monitored by TLC). The reaction mixture was evaporated under reduced pressure, the obtained residue diluted with water (60 mL) and extracted with diethyl ether (3 × 60 mL). The combined organic layers were washed with water (60 mL) and brine (60 mL). The organic phase was dried over anhydrous magnesium sulfate. Filtration and evaporation of the solvent at reduced pressure gave a crude solid, which was subjected to flash column chromatography with a gradient elution of petroleum ether (40–65°C)/EtOAc from 11:1 to 8:1 (v/v) to afford **4** as a white solid (108 mg, 66%). Mp: 117.8-119.3°C. IR *v*_max_ (NaCl): 3361, 3110, 2923, 2853, 2748, 1738, 1725, 1691, 1458, 1377, 1237, 1195 cm^−1^. ^1^H NMR (600 MHz, CDCl_3_): δ_H_ 9.70 (1H, s, CHO), 6.58 (1H, s, H-3), 5.33 (1H, br t, *J* = 3.4 Hz, H-12), 4.51 (1H, m, H-6), 4.07–4.03 (2H, m, H-23), 3.61 (3H, s, COOCH_3_), 2.53 and 2.37 (each 1H, m, H-11), 2.25 (1H, d, *J* = 11 Hz, H-18), 2.12 (1H, m, H-9), 2.06 (3H, s, OCOCH_3_), 1.86 (1H, m, H-15a), 1.99 (1H, m, H-16a), 1.78 (1H, m, H-7a), 1.69 (1H, m, H-16b), 1.67 (1H, m, H-5), 1.64 (1H, m, H-22a), 1.60 (3H, s, H-25), 1.59 (1H, m, H-22b), 1.54 (1H, m, H-7b), 1.48 (1H, m, H-21a), 1.33 (3H, s, H-24), 1.29 (1H, m, H-19), 1.28 (1H, m, H-21b), 1.15 (3H, s, H-26), 1.04 (3H, s, H-27), 1.00 (1H, m, H-20), 1.02 (1H, m, H-15b), 0.94 (3H, d, *J* = 6.3 Hz, H-30), 0.86 (3H, d, *J* = 6.4 Hz, H-29). ^13^C NMR (150 MHz, CDCl_3_): δ_C_ 190.7 (*C*HO), 178.1 (*C*OOCH_3_, C-28), 171.1 (C_23_-O*C*O), 157.9 (C-2 and C-3), 137.3 (C-13), 126.8 (C-12), 69.8 (C-23), 67.9 (C-6), 57.1 (C-5), 53.0 (C-18), 51.6 (COO*C*H_3_), 50.5 (C-10), 48.2 (C-17), 47.9 (C-4), 44.8 (C-9), 42.9 (C-14), 41.9 (C-7), 41.2 (C-8), 39.0 (C-20), 38.9 (C-19), 36.7 (C-22), 30.7 (C-21), 28.3 (C-15), 27.2 (C-11), 24.2 (C-16), 24.0 (C-27), 21.3 (C-30), 21.0 (C_23_-OCO*C*H_3_), 20.8 (C-25), 20.2 (C-26), 17.6 (C-24), 17.2 (C-29). MS (LIT) *m/z* [M + Na]^+^: 563.39; HRMS (Q-TOF) *m*/*z* [M + Na]^+^ calculated for C_33_H_48_O_6_Na = 563.3349, found = 563.3348 (Δ = −0.18 ppm).

##### Methyl 2-formyl-6β-hydroxy-23-(2-furoyloxy)-A(1)-norursa-2,12-dien-28-oate (5)

To a stirred solution of **3** (200 mg, 0.40 mmol) in dry benzene (10 mL), 2-furoyl chloride (0.16 mL, 1.6 mmol, 4 eq.) and DMAP (196.34 mg, 1.6 mmol, 4 eq.) were added. After 2 h at 60°C under nitrogen atmosphere, the reaction was completed (monitored by TLC). The reaction mixture was evaporated under reduced pressure, the obtained residue was diluted with water (60 mL) and extracted with diethyl ether (3 × 60 mL). The combined organic layers were washed with water (3 × 60 mL) and brine (60 mL). The organic phase was dried over anhydrous magnesium sulfate. Filtration and evaporation of the solvent at reduced pressure gave a crude solid, which was subjected to flash column chromatography with a gradient elution of petroleum ether (40–65°C)/EtOAc from 7:1 to 2:1 (v/v) to afford **5** as a white solid (154 mg, 65%). Mp: 142.7–143.9°C. IR *v*_max_ (NaCl): 3566, 3073, 2923, 2853, 2737, 1730, 1722, 1689, 1457, 1379, 1294, 1180 cm^−1^. ^1^H NMR (600 MHz, CDCl_3_): δ_H_ 9.71 (1H, s, CHO), 7.58 (1H, m, H-5′), 7.14 (1H, br d, *J* = 3.4 Hz, H-3′), 6.65 (1H, s, H-3), 6.52 (1H, m, H-4′), 5.32 (1H, br t, *J* = 3.5 Hz, H-12), 4.58 (1H, m, H-6), 4.32 and 4.25 (each 1H, d, *J* = 11 Hz, H-23), 3.61 (3H, s, COOCH_3_), 2.53 and 2.35 (each 1H, m, H-11), 2.24 (1H, d, *J* = 11 Hz, H-18), 2.11 (1H, m, H-9), 1.97 (1H, m, H-16a), 1.85 (1H, m, H-15a), 1.81 (1H, m, H-5), 1.79 (1H, m, H-7a), 1.67 (1H, m, H-16b), 1.64 (1H, m, H-22a), 1.61 (3H, s, H-25), 1.60 (1H, m, H-22b), 1.52 (1H, m, H-7b), 1.48 (1H, m, H-21a), 1.40 (3H, s, H-24), 1.28 (1H, m, C-21b), 1.27 (1H, m, H-19), 1.15 (3H, s, H-26), 1.01 (1H, m, H-15b), 0.99 (1H, m, H-20), 0.96 (3H, s, H-27), 0.94 (3H, d, *J* = 6.4 Hz, H-30), 0.85 (3H, d, *J* = 6.4 Hz, H-29). ^13^C NMR (150 MHz, CDCl_3_): δ_C_ 190.7 (*C*HO), 178.1 (*C*OOCH_3_, C-28), 158.6 (C_23_-O*C*O), 158.1 (C-2), 157.9 (C-3), 146.9 (C-5′), 144.3 (C-2′), 137.3 (C-13), 126.7 (C-12), 118.4 (C-3′), 112.1 (C-4′), 69.7 (C-23), 67.9 (C-6), 56.9 (C-5), 53.0 (C-18), 51.7 (COO*C*H_3_), 50.6 (C-10), 48.3 (C-17), 48.2 (C-4), 44.8 (C-9), 42.9 (C-14), 41.9 (C-7), 41.2 (C-8), 39.0 (C-20), 38.9 (C-19), 36.7 (C-22), 30.7 (C-21), 28.3 (C-15), 27.2 (C-11), 24.2 (C-16), 23.8 (C-27), 21.3 (C-30), 20.8 (C-25), 20.2 (C-26), 17.7 (C-24), 17.3 (C-29). MS (LIT) *m/z* [M + Na]^+^: 615.39; HRMS (Q-TOF) *m*/*z* [M + Na]^+^ calculated for C_36_H_48_O_7_Na = 615.3298, found = 615.3297 (Δ = −0.16 ppm).

##### Methyl 2α,3β,23-triacetyloxy-6β-hydroxyurs-12-en-28-oate (6)

To a solution of **2** (200 mg, 0.39 mmol) in dry THF (2 mL), acetic anhydride (0.29 mL, 3.12 mmol, 8 eq.) and a catalytic amount of DMAP (20 mg, 0.16 mmol) were added. After 1 h 30 min at room temperature the reaction was completed (monitored by TLC). The reaction mixture was evaporated under reduced pressure, the obtained residue diluted with water (60 mL) and extracted with diethyl ether (3 × 60 mL). The combined organic layers were washed with 5% aqueous HCl (3 × 30 mL), 10% aqueous NaHCO_3_ (3 × 30 mL), water (30 mL), and brine (30 mL). The organic phase was dried over anhydrous magnesium sulfate. Filtration and evaporation of the solvent at reduced pressure gave a crude solid, which was subjected to flash column chromatography with a gradient elution of petroleum ether (40–65°C)/EtOAc from 6:1 to 2:1 (v/v) to afford **6** as a white solid (171 mg, 68%). Mp: 131.7-132.5 °C. IR *v*_max_ (NaCl): 3524, 3025, 2923, 2853, 1751, 1740, 1730, 1456, 1371, 1233, 1045 cm^−1^. ^1^H NMR (600 MHz, CDCl_3_): δ_H_ 5.30 (1H, br t, *J* = 3.6 Hz, H-12), 5.23 (1H, dt, *J* = 11, 4.8 Hz, H-2), 5.01 (1H, d, *J* = 10 Hz, H-3), 4.35 (1H, br s, H-6), 3.95 and 3.70 (each 1H, d, *J* = 12 Hz, H-23), 3.60 (3H, s, COOCH_3_), 2.26 (1H, d, *J* = 11 Hz, H-18), 2.09 (1H, m, H-11a), 2.06 (3H, s, C_23_-OCOCH_3_), 2.04 (1H, m, H-1a), 2.03 (3H, s, C_3_-OCOCH_3_), 1.99 (1H, m, H-16a), 1.98 (3H, s, C_2_-OCOCH_3_), 1.96 (1H, m, H-11b), 1.82 (1H, m, H-15a), 1.75 (1H, dd, *J* = 15, 3.3 Hz, H-7a), 1.68 (1H, m, H-16b), 1.67 (1H, m, H-9), 1.66 and 1.59 (each 1H, m, H-22), 1.49 (1H, m, H-21a), 1.48 (3H, s, H-25), 1.47 (1H, m, H-7b), 1.37 (1H, br s, H-5), 1.32 (1H, m, H-19), 1.28 (1H, m, H-21b), 1.27 (3H, s, H-24), 1.11 (1H, m, H-1b), 1.05 (1H, m, H-15b), 1.04 (3H, s, H-27), 1.03 (3H, s, H-26), 1.01 (1H, m, H-20), 0.95 (3H, d, *J* = 6.3 Hz, H-30), 0.86 (3H, d, *J* = 6.5 Hz, H-29). ^13^C NMR (150 MHz, CDCl_3_): δ_C_ 178.1 (*C*OOCH_3_, C-28), 171.0 (C_23_-O*C*O), 170.6 (C_2_-O*C*O), 170.5 (C_3_-O*C*O), 137.5 (C-13), 125.6 (C-12), 75.0 (C-3), 70.0 (C-2), 68.1 (C-6), 65.4 (C-23), 53.0 (C-18), 51.7 (COO*C*H_3_), 48.2 (C-5 and C-17), 47.9 (C-9), 46.0 (C-1), 42.6 (C-4 and C-14), 41.2 (C-7), 39.2 (C-19), 39.0 (C-20), 38.8 (C-10), 37.5 (C-8), 36.7 (C-22), 30.8 (C-21), 28.0 (C-15), 24.3 (C-16), 23.7 (C-27), 23.5 (C-11), 21.3 (C-30), 21.2 (C_2_-OCO*C*H_3_), 21.0 (C_23_-OCO*C*H_3_), 20.9 (C_3_-OCO*C*H_3_), 18.7 (C-25), 18.6 (C-26), 17.1 (C-29), 15.5 (C-24). MS (LIT) *m/z* [M + Na]^+^: 667.45; HRMS (Q-TOF) *m/z* [M + Na]^+^ calculated for C_37_H_56_O_9_Na = 667.3822, found = 667.3825 (Δ = 0.45 ppm).

##### Methyl 2α,3β,23-triacetyloxy-6-oxours-12-en-28-oate (7)

A solution of **6** (150 mg, 0.23 mmol) in acetone (2 mL), cooled in ice-salt, was treated dropwise with Jones reagent (0.4 mL). The reaction mixture was stirred at 0°C for 30 min, and methanol (3 mL) was added to quench excess Jones reagent. Then the reaction mixture was allowed to warm up to room temperature and stirred for 15 min. The solvents were evaporated under reduced pressure, the obtained residue diluted with water (60 mL) and extracted with diethyl ether (3 × 60 mL). The combined organic layers were washed with water (3 × 60 mL) and brine (60 mL). The organic phase was dried over anhydrous magnesium sulfate. Filtration and evaporation of the solvent at reduced pressure gave a crude solid, which was subjected to flash column chromatography with a gradient elution of petroleum ether (40–65°C)/EtOAc from 5:1 to 2:1 (v/v) to afford **7** as a white solid (111 mg, 75%). Mp: 136.8–137.1°C. IR *v*_max_ (NaCl): 3023, 2923, 2853, 1746, 1738, 1732, 1715, 1456, 1367, 1231, 1046 cm^−1^. ^1^H NMR (600 MHz, CDCl_3_): δ_H_ 5.31 (1H, br t, *J* = 3.4 Hz, H-12), 5.14 (1H, dt, *J* = 11, 4.8 Hz, H-2), 4.95 (1H, d, *J* = 10 Hz, H-3), 3.93 and 3.78 (each 1H, d, *J* = 11 Hz, H-23), 3.59 (3H, s, COOCH_3_), 5.31 (1H, br t, *J* = 3.4 Hz, H-12), 2.66 (1H, s, H-5), 2.50 (1H, d, *J* = 13 Hz, H-7a), 2.30 (1H, d, *J* = 11 Hz, H-18), 2.18 (1H, m, H-1a), 2.17 (1H, m, H-9), 2.06 (1H, m, H-11a), 2.05 (3H, s, C_23_-OCOCH_3_), 2.04 (1H, m, H-16a), 2.03 (3H, s, C_3_-OCOCH_3_), 1.98 (3H, s, C_2_-OCOCH_3_), 1.93 (1H, d, *J* = 13 Hz, H-7b), 1.71 (1H, m, H-11b), 1.71 (1H, m, H-16b), 1.70 (1H, m, H-15a), 1.68 and 1.59 (each 1H, m, H-22), 1.51 (1H, m, H-21a), 1.37 (1H, m, H-1b), 1.33 (1H, m, H-19), 1.32 (3H, s, H-24), 1.29 (1H, m, H-21b), 1.20 (3H, s, H-27), 1.11 (3H, s, H-25), 1.02 (1H, m, H-20), 0.97 (1H, m, H-15b), 0.96 (3H, d, *J* = 6.3 Hz, H-30), 0.88 (3H, d, *J* = 6.4 Hz, H-29), 0.81 (3H, s, H-26). ^13^C NMR (150 MHz, CDCl_3_): δ_C_ 210.3 (*C* = O, C-6), 177.8 (*C*OOCH_3_, C-28), 170.6 (C_23_-O*C*O), 170.4 (C_2_-O*C*O), 170.4 (C_3_-O*C*O), 138.2 (C-13), 124.8 (C-12), 73.7 (C-3), 69.3 (C-2), 65.6 (C-23), 57.5 (C-5), 52.8 (C-18), 51.7 (COO*C*H_3_), 50.1 (C-7), 48.1 (C-9 and C-17), 46.2 (C-8), 44.1 (C-1), 43.4 (C-10), 42.5 (C-14), 40.7 (C-4), 39.0 (C-19), 38.9 (C-20), 36.5 (C-22), 30.7 (C-21), 28.0 (C-15), 24.1 (C-16 and C-27), 23.9 (C-11), 21.3 (C-30), 21.3 (C_2_-OCO*C*H_3_), 21.0 (C_23_-OCO*C*H_3_), 20.9 (C_3_-OCO*C*H_3_), 18.5 (C-25), 17.7 (C-26), 17.1 (C-29), 14.2 (C-24). MS (LIT) *m/z* [M + Na]^+^: 665.43; HRMS (Q-TOF) *m*/*z* [M + Na]^+^ calculated for C_37_H_54_O_9_Na = 665.3666, found = 665.3668 (Δ = 0.30 ppm).

##### Methyl 2α,3β,23-trihydroxy-6-oxours-12-en-28-oate (8)

A solution of **7** (160 mg, 0.25 mmol) and KOH (900 mg, 16.04 mmol) in methanol (9 mL), was heated under reflux for 2 h. After removal of the methanol under vacuum, the resultant mixture was acidified with 1M aqueous HCl solution and extracted with diethyl ether (3 × 60 mL). The combined organic layers were washed with 10% aqueous NaHCO_3_ (3 × 30 mL), water (3 × 30 mL), and brine (30 mL). The organic phase was dried over anhydrous magnesium sulfate. Filtration and evaporation of the solvent at reduced pressure gave a crude solid, which was subjected to flash column chromatography with a gradient elution of petroleum ether (40–65°C)/EtOAc from 4:1 to 1:2 (v/v) to afford **8** as a white solid (84 mg, 65%). Mp: 188.2-190.1 °C. IR *v*_max_ (NaCl): 3391, 3036, 2923, 2855, 1732, 1716, 1456, 1379, 1232, 1037 cm^−1^. ^1^H NMR (600 MHz, CDCl_3_): δ_H_ 5.30 (1H, br t, *J* = 3.4 Hz, H-12), 3.75 (1H, dt, *J* = 11, 4.6 Hz, H-2), 3.63 (1H, d, *J* = 10 Hz, H-23a), 3.59 (3H, s, COOCH_3_), 3.46 (1H, d, *J* = 10 Hz, H-23b), 3.34 (1H, d, *J* = 9.2 Hz, H-3), 2.54 (1H, d, *J* = 13 Hz, H-7a), 2.48 (1H, s, H-5), 2.27 (1H, d, *J* = 11 Hz, H-18), 2.14 (1H, m, H-9), 2.09 (1H, m, H-1a), 2.06 (1H, m, H-11a), 2.00 (1H, m, H-16a), 1.89 (1H, d, *J* = 13 Hz, H-7b), 1.73 (1H, m, H-15a), 1.69 (1H, m, H-11b), 1.67 (1H, m, H-16b), 1.66 and 1.57 (each 1H, m, H-22a), 1.49 and 1.26 (each 1H, m, H-21), 1.31 (1H, m, H-19), 1.21 (1H, m, H-1b), 1.20 (6H, H-24 and H-27), 1.03 (3H, s, H-25), 1.00 (1H, m, H-20), 0.96 (3H, d, *J* = 6.3 Hz, H-30), 0.95 (1H, m, H-15b), 0.88 (3H, d, *J* = 6.4 Hz, H-29), 0.79 (3H, s, H-26). ^13^C NMR (150 MHz, CDCl_3_): δ_C_ 212.1 (*C* = O, C-6), 177.8 (*C*OOCH_3_, C-28), 138.0 (C-13), 124.9 (C-12), 78.2 (C-3), 68.1 (C-2), 67.6 (C-23), 58.2 (C-5), 52.7 (C-18), 51.6 (COO*C*H_3_), 50.2 (C-7), 48.0 (C-17), 47.8 (C-9), 46.4 (C-8), 46.2 (C-1), 43.6 (C-10), 42.4 (C-4), 41.7 (C-14), 38.9 (C-19), 38.8 (C-20), 36.4 (C-22), 30.5 (C-21), 27.9 (C-15), 24.2 (C-16), 24.0 (C-27), 23.7 (C-11), 21.2 (C-30), 18.4 (C-25), 17.5 (C-26), 17.1 (C-29), 12.9 (C-24). MS (LIT) *m/z* [M + Na]^+^: 539.38; HRMS (Q-TOF) *m/z* [M + Na]^+^ calculated for C_31_H_48_O_6_Na = 539.3349, found = 539.3351 (Δ = 0.37 ppm).

##### Methyl 2α-hydroxy-a(2)-homo-3-formyl-6-oxours-12-en-28-oate (9)

To a solution of **8** (400 mg, 0.77 mmol) in methanol/water (7.8 mL/0.4 mL, 20:1), sodium periodate (249.81 mg, 1.17 mmol) was added. After 2 h at room temperature the reaction was completed (monitored by TLC). The reaction mixture was evaporated under reduced pressure, the obtained residue diluted with water (100 mL) and extracted with diethyl ether (3 × 100 mL). The combined organic layers were washed with water (3 × 100 mL) and brine (100 mL). The organic phase was dried over anhydrous magnesium sulfate. Filtration and evaporation of the solvent at reduced pressure gave a crude solid, which was subjected to flash column chromatography with a gradient elution of petroleum ether (40–65°C)/EtOAc from 3:1 to 2:1 (v/v) to afford **9** as a white solid (309 mg, 78%). Mp: 134.9–136.1°C. IR *v*_max_ (NaCl): 3452, 3062, 2923, 2853, 1732, 1722, 1717, 1651, 1456, 1378, 1231, 1195, 1037 cm^−1^. ^1^H NMR (600 MHz, CDCl_3_): δ_H_ 10.23 (1H, s, H-3), 5.34 (1H, m, H-12), 5.12 (1H, dd, *J* = 10, 5.0 Hz, H-2), 3.89 and 3.57 (each 1H, d, *J* = 13 Hz, H-23), 3.59 (3H, s, COOCH_3_), 2.62 (1H, s, H-5), 2.54 (1H, m, H-7a), 2.35 (1H, dd, *J* = 15, 5.2 Hz, H-1a), 2.29 (1H, d, *J* = 12Hz, H-18), 2.21 (1H, m, H-9), 2.09 (1H, m, H-11a), 2.08 (1H, m, H-16a), 2.04 (1H, m, H-7b), 1.98 (1H, m, H-11b), 1.78 (1H, m, H-1b), 1.76 (1H, m, H-15a), 1.75 (1H, m, H-16b), 1.70 and 1.59 (each 1H, m, H-22), 1.54 (1H, m, H-21a), 1.32 (1H, m, H-19), 1.31 (1H, m, H-21b), 1.24 (3H, s, H-27), 1.23 (3H, s, H-25), 1.05 (1H, m, H-15b), 0.97 (1H, m, H-20), 0.95 (3H, d, *J* = 6.1 Hz, H-30), 0.92 (3H, s, H-24), 0.88 (3H, d, *J* = 6.5 Hz, H-29), 0.87 (3H, s, H-26). ^13^C NMR (150 MHz, CDCl_3_): δ_C_ 212.7 (*C* = O, C-6), 206.1 (*C*HO, C-3), 177.8 (*C*OOCH_3_, C-28), 138.1 (C-13), 125.3 (C-12), 93.4 (C-2), 70.2 (C-5), 67.4 (C-23), 52.8 (C-18), 52.3 (C-4), 51.7 (COO*C*H_3_), 49.3 (C-7), 48.1 (C-17), 47.1 (C-10), 47.0 (C-8), 44.6 (C-1), 44.4 (C-9), 43.0 (C-14), 39.0 (C-19), 38.9 (C-20), 36.5 (C-22), 30.7 (C-21), 28.0 (C-15), 25.0 (C-11), 24.1 (C-16), 24.0 (C-27), 21.2 (C-30), 20.9 (C-24), 18.1 (C-26), 17.2 (C-29), 16.3 (C-25). MS (LIT) *m/z* [M + Na]^+^: 537.37; HRMS (Q-TOF) *m*/*z* [M + H]^+^ calculated for C_31_H_47_O_6_ = 515.3373, found = 515.3374 (Δ = 0.19 ppm).

##### Methyl 2-formyl-6-hydroxy-6,23-epoxy-a(1)-norursa-2,12-dien-28-oate (10)

To a solution of **9** (380 mg, 0.74 mmol) in dry benzene (37.5 mL), piperidine (1.9 mL, 19.26 mmol) and glacial acetic acid (1.9 mL, 33.20 mmol) were added. The reaction mixture was heated at 60°C for 1 h under nitrogen atmosphere. Anhydrous magnesium sulfate (380 mg, 3.16 mmol) was then added and the reaction continued for another 2 h. The reaction mixture was evaporated under reduced pressure, the obtained residue diluted with water (100 mL) and extracted with diethyl ether (3 × 100 mL). The combined organic layers were washed with water (3 × 100 mL) and brine (100 mL). The organic phase was dried over anhydrous magnesium sulfate. Filtration and evaporation of the solvent at reduced pressure gave a crude solid, which was subjected to flash column chromatography with a gradient elution of petroleum ether (40–65°C)/EtOAc from 2:1 to 1:1 (v/v) to afford **10** as a white solid (255 mg, 69%). Mp: 113.2–114.8°C. IR *v*_max_ (NaCl): 3442, 3065, 2924, 2854, 2728, 1733, 1683, 1652, 1456, 1379, 1228, 1195 cm^−1^. ^1^H NMR (600 MHz, C_6_D_6_): δ_H_ 9.46 (1H, s, CHO), 6.40 (1H, s, H-3), 5.60 (2H, m, H-3 and H-12), 3.79 and 3.60 (each 1H, d, *J* = 8.6 Hz, H-23), 3.43 (3H, s, COOCH_3_), 2.89 (1H, m, H-11a), 2.50 (1H, d, *J* = 11 Hz, H-18), 2.38 (1H, m, H-9), 2.37 (1H, m, H-11b), 2.00 (1H, m, H-16a), 2.11 (1H, s, H-5), 1.87 (1H, m, H-7a), 1.85 (1H, m, H-15a), 1.67 (1H, m, H-16b), 1.66 (1H, m, H-22a), 1.65 (1H, m, H-7b), 1.59 (1H, m, H-22b), 1.48 (1H, m, H-21a), 1.33 (1H, m, H-19), 1.26 (1H, m, H-21b), 1.15 (3H, s, H-25), 1.08 (1H, m, H-15b), 1.06 (3H, s, H-27), 1.01 (3H, s, H-26), 1.00 (3H, s, H-24), 0.98 (1H, m, H-20), 0.93 (3H, d, *J* = 6.1 Hz, H-29) and 0.84 (3H, m, H-30). ^13^C NMR (150 MHz, C_6_D_6_): δ_C_ 189.4 (*C*HO), 177.5 (*C*OOCH_3_, C-28), 156.2 (C-3), 156.0 (C-2), 136.8 (C-13), 127.9 (C-12), 107.3 (C-6), 74.6 (C-23), 66.6 (C-5), 57.7 (C-4), 54.0 (C-18), 51.2 (COO*C*H_3_), 49.6 (C-10), 48.8 (C-17), 44.3 (C-7), 42.4 (C-8), 39.7 (C-14), 39.6 (C-19), 39.2 (C-20), 38.7 (C-9), 37.0 (C-22), 31.1 (C-21), 28.9 (C-15), 28.1 (C-25), 27.2 (C-11), 24.8 (C-16), 23.9 (C-27), 21.7 (C-24), 21.2 (C-30), 17.8 (C-26), 17.3 (C-29). MS (LIT) *m/z* [M + Na]^+^: 519.32; HRMS (Q-TOF) *m*/*z* [M + Na]^+^ calculated for C_31_H_44_O_5_Na = 519.3086, found = 519.3086 (Δ = 0 ppm).

##### Methyl 2-formyl-6-oxo-23-acetyloxy-A(1)-norursa-2,12-dien-28-oate (11)

Prepared accordingly to the method described for **4** using **10** (200 mg, 0.40 mmol), dry THF (2 mL), acetic anhydride (0.11 mL, 1.2 mmol, 3 eq.) and DMAP (20 mg, 0.16 mmol). After 1 h 30 min at room temperature, the reaction was completed (monitored by TLC). The crude solid was subjected to flash column chromatography with a gradient elution of petroleum ether (40–65°C)/EtOAc from 12:1 to 8:1 (v/v) to afford **11** as a white solid (131 mg, 61%). Mp: 79.8–81.6°C. IR *v*_max_ (NaCl): 3063, 2923, 2855, 2735, 1742, 1739, 1717, 1689, 1457, 1372, 1231, 1195 cm^−1^. ^1^H NMR (600 MHz, CDCl_3_): δ_H_ 9.73 (1H, s, CHO), 6.52 (1H, s, H-3), 5.38 (1H, m, H-12), 4.34 and 4.09 (each 1H, d, *J* = 11 Hz, H-23), 3.60 (3H, s, COOCH_3_), 2.53 (1H, s, H-5), 2.51 (1H, m, H-7a), 2.27 (1H, d, *J* = 12 Hz, H-18), 2.26 and 2.18 (each 1H, m, H-11), 2.17 (1H, m, H-9), 2.16 (1H, m, H-7b), 2.01 (3H, s, C_23_-OCOCH_3_), 2.00 (1H, m, H-16a), 1.73 (1H, m, H-15a), 1.71 (1H, m, H-16b), 1.67 and 1.58 (each 1H, m, H-22), 1.49 (1H, m, H-21a), 1.48 (3H, s, H-25), 1.40 (3H, s, H-24), 1.30 (1H, m, H-19), 1.27 (1H, m, H-21b), 1.18 (1H, m, H-15b), 1.05 (3H, s, H-27), 0.99 (1H, m, H-20), 0.92 (3H, d, *J* = 6.4 Hz, H-30), 0.90 (3H, s, H-26), 0.82 (3H, d, *J* = 6.4 Hz, H-29). ^13^C NMR (150 MHz, CDCl_3_): δ_C_ 212.7 (*C* = O, C-6), 190.3 (*C*HO), 177.9 (*C*OOCH_3_, C-28), 170.5 (C_23_-O*C*O), 157.2 (C-3), 153.3 (C-2), 137.5 (C-13), 127.0 (C-12), 69.5 (C-5), 67.9 (C-23), 53.6 (C-18), 52.9 (C-10), 51.8 (C-4), 51.7 (COO*C*H_3_), 48.5 (C-17), 48.2 (C-7), 43.4 (C-8), 43.3 (C-14), 41.7 (C-9), 39.0 (C-19 and C-20), 36.5 (C-22), 30.7 (C-21), 27.9 (C-15), 26.9 (C-11), 26.7 (C-24), 26.0 (C-25), 24.2 (C-16), 23.6 (C-27), 21.2 (C-30), 21.1 (C_23_-OCO*C*H_3_), 18.9 (C-26), 17.2 (C-29). MS (LIT) *m/z* [M + Na]^+^: 561.36; HRMS (Q-TOF) *m*/*z* [M + Na]^+^ calculated for C_33_H_46_O_6_Na = 561.3192, found = 561.3194 (Δ = 0.36 ppm).

##### Methyl 2-formyl-23-(2-furoyloxy)-6-oxo-A(1)-norursa-2,12-dien-28-oate (12)

Prepared accordingly to the method described for **5** using **10** (200 mg, 0.40 mmol), dry benzene (10 mL), 2-furoyl chloride (0.16 mL, 1.6 mmol, 4 eq.) and DMAP (196.34 mg, 1.6 mmol, 4 eq.). After 2 h at 60°C under nitrogen atmosphere, the reaction was completed (monitored by TLC). The crude solid was subjected to flash column chromatography with a gradient elution of petroleum ether (40–65°C)/EtOAc from 7:1 to 6:1 (v/v) to afford **12** as a white solid (132 mg, 56%). Mp: 98.6–100.2°C. IR *v*_max_ (NaCl): 3112, 3076, 2924, 2855, 2735, 1733, 1723, 1717, 1683, 1471, 1397, 1231, 1178, 763 cm^−1^. ^1^H NMR (600 MHz, CDCl_3_): δ_H_ 9.75 (1H, s, CHO), 7.55 (1H, m, H-5′), 7.17 (1H, br d, *J* = 3.4 Hz, H-3′), 6.59 (1H, s, H-3), 6.51 (1H, m, H-4′), 5.38 (1H, m, H-12), 4.47 and 4.31 (each 1H, d, *J* = 11 Hz, H-23), 3.59 (3H, s, COOCH_3_), 2.59 (1H, s, H-5), 2.58 (1H, d, *J* = 15 Hz, H-7a), 2.27 (1H, d, *J* = 11 Hz, H-18), 2.26 and 2.20 (each H, m, H-11), 2.19 (1H, m, H-9), 2.15 (1H, d, *J* = 15 Hz, H-7b), 1.98 (1H, m, H-16a), 1.70 (1H, m, H-15a), 1.69 (1H, m, H-16b), 1.66 and 1.58 (each 1H, m, H-22), 1.48 (1H, m, H-21a), 1.47 (6H, s, H-24 and H-25), 1.28 (1H, m, H-19), 1.26 (1H, m, H-21b), 1.14 (1H, m, H-15b), 1.03 (3H, s, H-27), 0.98 (1H, m, H-20), 0.92 (3H, d, *J* = 6.3 Hz, H-30), 0.87 (3H, s, H-26), 0.81 (3H, d, *J* = 6.4 Hz, H-29). ^13^C NMR (150 MHz, CDCl_3_): δ_C_ 212.7 (*C* = O, C-6), 190.3 (*C*HO), 177.9 (*C*OOCH_3_, C-28), 158.2 (C_23_-O*C*O), 156.6 (C-3), 153.8 (C-2), 146.7 (C-5′), 144.2 (C-2′), 137.3 (C-13), 126.9 (C-12), 118.8 (C-3′), 112.2 (C-4′), 69.6 (C-5), 68.7 (C-23), 53.5 (C-18), 52.9 (C-10), 52.0 (C-4), 51.7 (COO*C*H_3_), 48.5 (C-17), 48.2 (C-7), 43.4 (C-14), 43.0 (C-8), 41.8 (C-9), 39.0 (C-19), 38.9 (C-20), 36.5 (C-22), 30.8 (C-21), 28.0 (C-15), 26.9 (C-11), 26.6 (C-24), 26.1 (C-25), 24.2 (C-16), 23.6 (C-27), 21.2 (C-30), 18.6 (C-26), 17.2 (C-29). MS (LIT) *m/z* [M + Na]^+^: 613.35; HRMS (Q-TOF) *m*/*z* [M + Na]^+^ calculated for C_36_H_46_O_7_Na = 613.3141, found = 613.3150 (Δ = 1.47 ppm).

##### Methyl 2α,3β,23-triacetyloxyursa-5,12-dien-28-oate (13)

To a solution of **6** (250 mg, 0.39 mmol) in pyridine (0.7 mL), thionyl chloride (0.7 mL) was slowly added. After 1 h 30 min at room temperature the reaction was completed (monitored by TLC). Cold water (1 mL) was added dropwise to the solution. *Caution: This reaction is highly exothermic and a vigorous expulsion of SO*_2_ *was observed. All manipulations with this reagent should be carried out in a fume hood, and full face protection should be worn when the window of the hood is raised*. After gas evolution ceases, additional cold water (59 mL) was added to the reaction mixture followed by extraction with diethyl ether (3 × 60 mL). The combined organic layers were washed with water (3 × 60 mL) and brine (60 mL). The organic phase was dried over anhydrous magnesium sulfate. Filtration and evaporation of the solvent at reduced pressure gave a crude solid, which was subjected to flash column chromatography with an isocratic elution of petroleum ether (40–65°C)/EtOAc 4:1 (v/v) to afford **13** as a white solid (183 mg, 75%). Mp: 93.6–95.1°C. IR *v*_max_ (NaCl): 3033, 2921, 2853, 1742, 1739, 1736, 1652, 1456, 1377, 1234 cm^−1^. ^1^H NMR (600 MHz, CDCl_3_): δ_H_ 5.56 (1H, m, H-6), 5.37 (1H, br t, *J* = 3.6 Hz, H-12), 5.31 (1H, dt, *J* = 11.2, 6.8 Hz, H-2), 5.15 (1H, d, *J* = 11 Hz, H-3), 4.25 and 3.67 (each 1H, d, *J* = 12 Hz, H-23), 3.60 (3H, s, COOCH_3_), 2.36 (1H, dd, *J* = 19, 6.3 Hz, H-7a), 2.27 (1H, d, *J* = 11 Hz, H-18), 2.05 (3H, s, C_3_-OCOCH_3_), 2.02 (1H, m, H-11a), 2.00 (3H, s, C_2_-OCOCH_3_), 1.99 (1H, m, H-1a), 1.98 (3H, s, C_23_-OCOCH_3_), 1.96 (1H, m, H-11b), 1.95 (1H, m, H-16a), 1.75 (1H, m, H-15a), 1.73 (1H, m, H-9), 1.69 (1H, m, H-16b), 1.65 (1H, m, H-22a), 1.62 (1H, m, H-7b), 1.57 (1H, m, H-22b), 1.48 (1H, m, H-21a), 1.34 (1H, m, H-1b), 1.28 (1H, m, H-21b), 1.27 (1H, m, H-19), 1.22 (3H, s, H-25), 1.17 (1H, m, H-15b), 1.12 (3H, s, H-24), 0.99 (1H, m, H-20), 0.95 (3H, s, H-27), 0.94 (3H, d, *J* = 6.0 Hz, H-30), 0.90 (3H, s, H-26), 0.85 (3H, d, *J* = 6.3 Hz, H-29). ^13^C NMR (150 MHz, CDCl_3_): δ_C_ 178.1 (*C*OOCH_3_, C-28), 171.2 (C_23_-O*C*O), 170.6 (C_2_-O*C*O), 170.4 (C_3_-O*C*O), 144.2 (C-5), 139.4 (C-13), 126.5 (C-12), 122.8 (C-6), 73.7 (C-3), 69.2 (C-2), 65.1 (C-23), 53.6 (C-18), 51.6 (COO*C*H_3_), 48.6 (C-17), 46.2 (C-9), 45.2 (C-4), 43.6 (C-14), 42.5 (C-1), 39.0 (C-20), 38.6 (C-19), 38.4 (C-8 and C-10), 36.5 (C-22), 32.4 (C-7), 30.7 (C-21), 27.4 (C-15), 24.1 (C-16), 23.6 (C-11), 23.1 (C-27), 22.4 (C-25), 22.1 (C-24), 21.4 (C-26), 21.2 (C-30 and C_2_-OCO*C*H_3_), 21.02 (C_23_-OCO*C*H_3_), 20.97 (C_3_-OCO*C*H_3_), 17.2 (C-29). MS (LIT) *m/z* [M + Na]^+^: 649.37; HRMS (Q-TOF) *m*/*z* [M + Na]^+^ calculated for C_37_H_54_O_8_Na = 649.3716, found = 649.3717 (Δ = 0.15 ppm).

##### Methyl 2α,3β,23-trihydroxyursa-5,12-dien-28-oate (14)

Prepared accordingly to the method described for **8** using **13** (400 mg, 0.64 mmol), methanol (22 mL) and KOH (2.21 g). The reaction mixture was stirred at reflux temperature for 2 h. The crude solid was subjected to flash column chromatography with a gradient elution of petroleum ether (40–65°C)/EtOAc from 1:1 to 1:2 (v/v) to afford **14** as a white solid (269 mg, 84%). Mp: 129.8–131.4°C. IR *v*_max_ (NaCl): 3452, 3027, 2923, 2854, 1741, 1645, 1464, 1377, 1242, 1168 cm^−1^. ^1^H NMR (600 MHz, CDCl_3_): δ_H_ 5.56 (1H, m, H-6), 5.36 (1H, br t, *J* = 3.6 Hz, H-12), 3.92 (1H, m, H-2), 3.73 and 3.65 (each 1H, d, *J* = 11 Hz, H-23), 3.60 (3H, s, COOCH_3_), 3.53 (1H, d, *J* = 10 Hz, H-3), 2.38 (1H, dd, *J* = 19, 5.9 Hz, H-7a), 2.27 (1H, d, *J* = 11 Hz, H-18), 2.01 (1H, m, H-11a), 1.99 (1H, m, H-16a), 1.97 (1H, m, H-11b), 1.92 (1H, m, H-1a), 1.75 (1H, m, H-15a), 1.69 (1H, m, H-9), 1.68 (1H, m, H-16b), 1.67 (1H, m, H-7b), 1.47 (1H, m, H-21a), 1.27 (1H, m, H-19), 1.26 (1H, m, H-22b), 1.15 (1H, m, H-15b), 1.16 (3H, s, H-25), 1.09 (1H, m, H-1b), 1.03 (3H, s, H-24), 0.99 (1H, m, H-20), 0.98 (3H, s, H-27), 0.94 (3H, d, *J* = 5.8 Hz, H-30), 0.90 (3H, s, H-26), 0.84 (3H, d, *J* = 6.4 Hz, H-29). ^13^C NMR (150 MHz, CDCl_3_): δ_C_ 178.1 (*C*OOCH_3_, C-28), 147.5 (C-5), 139.2 (C-13), 126.6 (C-12), 121.9 (C-6), 77.0 (C-3), 67.6 (C-2), 67.0 (C-23), 53.5 (C-18), 51.6 (COO*C*H_3_), 48.6 (C-17), 46.6 (C-4), 45.9 (C-9), 44.9 (C-1), 43.5 (C-14), 39.0 (C-8), 38.7 (C-20), 38.5 (C-19), 38.4 (C-10), 36.5 (C-22), 32.5 (C-7), 30.7 (C-21), 27.4 (C-15), 24.2 (C-16), 23.6 (C-11), 23.5 (C-27), 22.5 (C-25), 21.4 (C-26), 21.3 (C-30), 21.2 (C-24), 17.3 (C-29). MS (LIT) *m/z* [M + Na]^+^: 523.36; HRMS (Q-TOF) *m*/*z* [M + Na]^+^ calculated for C_31_H_48_O_5_Na = 523.3399, found = 523.3403 (Δ = 0.76 ppm).

##### Methyl 2-formyl-23-hydroxy-A(1)-norursa-2,5,12-trien-28-oate (15)

Prepared accordingly to the method described for **3** using **14** (240 mg, 0.48 mmol), methanol/water (5 mL/0.25 mL, 20:1) and sodium periodate (156.14 mg, 0.73 mmol). The reaction mixture was stirred at room temperature for 2 h and the solvents evaporated under reduced pressure. The obtained residue was then diluted with water (60 mL) and extracted with diethyl ether (3 × 60 mL). The combined organic layers were washed with water (3 × 60 mL) and brine (60 mL). The organic phase was dried over anhydrous magnesium sulfate. Filtration and evaporation of the solvent at reduced pressure gave a crude solid. Dry benzene (14.4 mL), piperidine (1.25 mL) and glacial acetic acid (1.25 mL) were added and the reaction mixture was heated at 60°C for 1 h under nitrogen atmosphere. Anhydrous magnesium sulfate (240 mg, 1.99 mmol) was then added and the reaction continued for another 1 h 30 min. The solvent was evaporated under reduced pressure, the obtained residue diluted with water (60 mL) and extracted with diethyl ether (3 × 60 mL). The combined organic layers were washed with water (3 × 60 mL) and brine (60 mL). The organic phase was dried over anhydrous magnesium sulfate. Filtration and evaporation of the solvent at reduced pressure gave a crude solid, which was subjected to flash column chromatography with an isocratic elution of petroleum ether (40–65°C)/EtOAc 4:1 (v/v) to afford **15** as a white solid (150 mg, 65%). Mp: 133.2–135.0°C. IR *v*_max_ (NaCl): 3447, 3100, 2924, 2868, 2740, 1734, 1684, 1653, 1457, 1375, 1231, 1195 cm^−1^. ^1^H NMR (600 MHz, CDCl_3_): δ_H_ 9.80 (1H, s, CHO), 6.68 (1H, s, H-3), 5.58 (1H, m, H-6), 5.41 (1H, m, H-12), 3.67 (1H, d, *J* = 11 Hz, H-23a), 3.61 (3H, s, COOCH_3_), 3.60 (1H, m, H-23b), 2.37 (1H, dd, *J* = 18, 7.6 Hz, H-7a), 2.30 (1H, m, H-11a), 2.27 (1H, d, *J* = 11 Hz, H-18), 2.00 (1H, m, H-16a), 1.91 (1H, m, H-11b), 1.80 (1H, m, H-7b), 1.79 (1H, m, H-15a), 1.71 (1H, m, H-16b), 1.65 (1H, m, H-22a), 1.64 (1H, m, H-9), 1.59 (1H, m, H-22b), 1.49 (1H, m, H-21a), 1.35 (3H, s, H-25), 1.27 (1H, m, H-21b), 1.25 (1H, m, H-19), 1.24 (1H, m, H-15b), 1.16 (3H, s, H-24), 1.01 (3H, s, H-26), 0.98 (1H, m, H-20), 0.91 (3H, s, H-27), 0.90 (3H, m, H-30), 0.77 (3H, d, *J* = 6.4 Hz, H-29). ^13^C NMR (150 MHz, CDCl_3_): δ_C_ 190.9 (*C*HO), 178.1 (*C*OOCH_3_, C-28), 158.5 (C-3), 155.1 (C-5), 153.6 (C-2), 139.0 (C-13), 128.5 (C-12), 118.5 (C-6), 68.5 (C-23), 53.9 (C-4 and C-18), 53.2 (C-10), 51.6 (COO*C*H_3_), 48.9 (C-17), 44.7 (C-9), 44.2 (C-14), 40.6 (C-8), 39.0 (C-20), 38.3 (C-19), 36.5 (C-22), 31.3 (C-7), 30.7 (C-21), 27.6 (C-15), 27.3 (C-11), 24.3 (C-16), 24.1 (C-24), 23.1 (C-27), 22.0 (C-26), 21.2 (C-30), 19.7 (C-25), 17.4 (C-29). MS (LIT) *m/z* [M + Na]^+^: 503.37; HRMS (Q-TOF) *m*/*z* [M + Na]^+^ calculated for C_31_H_44_O_4_Na = 503.3137, found = 503.3140 (Δ = 0.60 ppm).

##### Methyl 2-formyl-23-acetyloxy-A(1)-norursa-2,5,12-trien-28-oate (16)

Prepared accordingly to the method described for **4** using **15** (300 mg, 0.62 mmol), dry THF (3 mL), acetic anhydride (0.18 mL, 1.86 mmol, 3 eq.) and DMAP (30 mg, 0.25 mmol). After 1 h 30 min at room temperature, the reaction was completed (monitored by TLC). The crude solid was subjected to flash column chromatography with an isocratic elution of petroleum ether (40–65°C)/EtOAc 11:1 (v/v) to afford **16** as a white solid (202 mg, 62%). Mp: 77.5–79.1°C. IR *v*_max_ (NaCl): 3043, 2924, 2854, 2729, 1734, 1684, 1653, 1457, 1369, 1233, 1195 cm^−1^. ^1^H NMR (600 MHz, CDCl_3_): δ_H_ 9.79 (1H, s, CHO), 6.64 (1H, s, H-3), 5.58 (1H, m, H-6), 5.41 (1H, m, H-12), 4.24 and 3.93 (each 1H, d, *J* = 11 Hz, H-23), 3.61 (3H, s, COOCH_3_), 2.35 (1H, m, H-7a), 2.29 (1H, m, H-11a), 2.27 (1H, d, *J* = 11 Hz, H-18), 2.00 (1H, m, H-16a), 1.99 (3H, s, C_23_-OCOCH_3_), 1.88 (1H, m, H-11b), 1.78 (1H, m, H15a), 1.75 (1H, m, H-7b), 1.71 (1H, m, H-16b), 1.68 (1H, m, H-9), 1.65 and 1.59 (each 1H, m, H-22), 1.49 (1H, m, H-21a), 1.33 (3H, s, H-25), 1.29 (1H, m, H-19), 1.27 (1H, m, H-21b), 1.24 (1H, m, H-15b), 1.21 (3H, s, H-24), 0.99 (3H, s, H-26), 0.97 (1H, m, H-20), 0.91 (3H, d, *J* = 6.3 Hz, H-30), 0.89 (3H, s, H-27), 0.77 (3H, d, *J* = 6.4 Hz, H-29). ^13^C NMR (150 MHz, CDCl_3_): δ_C_ 191.0 (*C*HO), 178.1 (*C*OOCH_3_, C-28), 171.1 (C_23_-O*C*O), 156.7 (C-3), 154.4 (C-5), 153.1 (C-2), 139.2 (C-13), 128.4 (C-12), 118.7 (C-6), 69.7 (C-23), 54.1 (C-18), 53.2 (C-10), 51.6 (COO*C*H_3_), 50.9 (C-4), 48.9 (C-17), 44.9 (C-9), 44.3 (C-14), 40.7 (C-8), 39.0 (C-20), 38.4 (C-19), 36.5 (C-22), 31.3 (C-7), 30.8 (C-21), 27.9 (C-15), 27.2 (C-11), 24.5 (C-24), 24.3 (C-16), 22.9 (C-27), 22.0 (C-26), 21.2 (C-30), 21.1 (C_23_-OCO*C*H_3_), 19.8 (C-25), 17.2 (C-29). MS (LIT) *m/z* [M + Na]^+^: 545.45; HRMS (Q-TOF) *m*/*z* [M + Na]^+^ calculated for C_33_H_46_O_5_Na = 545.3243, found = 545.3239 (Δ = −0.73 ppm).

##### Methyl 2-formyl-23-(2-furoyloxy)-A(1)-norursa-2,5,12-trien-28-oate (17)

Prepared accordingly to the method described for **5** using **15** (200 mg, 0.42 mmol), dry benzene (10 mL), 2-furoyl chloride (0.17 mL, 1.68 mmol, 4 eq.) and DMAP (206.15 mg, 1.68 mmol, 4 eq.). After 1 h 30 min at 60°C under nitrogen atmosphere, the reaction was completed (monitored by TLC). The crude solid was subjected to flash column chromatography with an isocratic elution of petroleum ether (40–65°C)/EtOAc 12:1 (v/v) to afford **17** as a white solid (154 mg, 64%). Mp: 92.2–93.7°C. IR *v*_max_ (NaCl): 3050, 2925, 2870, 2743, 1733, 1717, 1689, 1653, 1456, 1377, 1293, 1180, 762 cm^−1^. ^1^H NMR (600 MHz, CDCl_3_): δ_H_ 9.82 (1H, s, CHO), 7.54 (1H, m, H-5′), 7.08 (1H, br d, *J* = 3.4 Hz, H-3′), 6.73 (1H, s, H-3), 6.46 (1H, m, H-4′), 5.66 (1H, m, H-6), 5.38 (1H, m, H-12), 4.34 and 4.31 (each 1H, d, *J* = 11 Hz, H-23), 3.60 (3H, s, COOCH_3_), 2.33 (1H, m, H-7a), 2.29 (1H, m, H-11a), 2.24 (1H, d, *J* = 11 Hz, H-18), 1.95 (1H, m, H-16a), 1.89 (1H, m, H-11b), 1.74 (1H, m, H-7b), 1.73 (1H, m, H-15a), 1.68 (1H, m, H-16b), 1.67 (1H, m, H-9), 1.63 and 1.57 (each 1H, m, H-22), 1.47 (1H, m, H-21a), 1.35 (3H, s, H-25), 1.27 (3H, s, H-24), 1.25 (1H, m, H-21b), 1.20 (1H, m, H-15b), 1.18 (1H, m, H-19), 0.98 (3H, s, H-26), 0.95 (1H, m, H-20), 0.90 (3H, d, *J* = 6.2 Hz, H-30), 0.74 (3H, s, H-27), 0.71 (3H, d, *J* = 6.4 Hz, H-29). ^13^C NMR (150 MHz, CDCl_3_): δ_C_ 191.0 (*C*HO), 178.1 (*C*OOCH_3_, C-28), 158.6 (C_23_-O*C*O), 156.6 (C-3), 154.1 (C-5), 153.3 (C-2), 146.6 (C-5′), 144.5 (C-2′), 139.2 (C-13), 128.2 (C-12), 118.8 (C-6), 118.2 (C-3′), 112.0 (C-4′), 69.6 (C-23), 54.0 (C-18), 53.1 (C-10), 51.6 (COO*C*H_3_), 51.1 (C-4), 48.9 (C-17), 44.8 (C-9), 44.2 (C-14), 40.6 (C-8), 38.9 (C-20), 38.3 (C-19), 36.4 (C-22), 31.3 (C-7), 30.7 (C-21), 27.8 (C-15), 27.2 (C-11), 24.6 (C-24), 24.3 (C-16), 22.7 (C-27), 22.0 (C-26), 21.2 (C-30), 20.0 (C-25), 17.5 (C-29). MS (LIT) *m/z* [M + Na]^+^: 597.40; HRMS (Q-TOF) *m*/*z* [M + Na]^+^ calculated for C_36_H_46_O_6_Na = 597.3192, found = 597.3200 (Δ = 1.34 ppm).

### Biology

#### NCI-60 anticancer drug screen

The NCI-60 cell line panel is organized into nine subpanels with diverse histology representing leukemia, melanoma, non-small cell lung, colon, kidney, ovarian, breast, prostate, and central nervous system cancers. Details of the NCI-60 cell line screening protocols and reporting procedures have been described previously (Monga and Sausville, 2002; Shoemaker, 2006; Holbeck et al., 2010). A complete list of cells in the NCI-60 panel and additional details are available at https://dtp.cancer.gov/organization/btb/docs/DCTDTumorRepositoryCatalog.pdf. All cultures were maintained at 37°C, 5% CO_2_, 95% air and 100% relative humidity. The NCI-60 cancer cell lines were grown in RPMI 1640 medium supplemented with 5% fetal bovine serum and 2 mM L-glutamine. Cells were dispersed into a series of 96-well microtiter plates at an appropriate density and incubated for 24 h in the absence of drug; some of the plates are then processed to determine the density at time zero. After 24 h, serial 10-fold dilutions of the experimental drugs over a 5-log mol/L concentration range were added for 48 h of exposure. The protein content was determined by sulforhodamine B staining after the cells were fixed in 10% trichloroacetic acid (TCA). The percentage growth inhibition was determined relative to cells without drug treatment and the time zero control. The use of the time zero control allows the evaluation of cell kill as well as net growth inhibition. Three endpoints are routinely calculated for each test sample. The GI_50_ is defined as the molar concentration of the compound that causes 50% growth inhibition relative to the control (only treated with DMSO), TGI, or total growth inhibition, is the molar concentration that yields no net growth over the course of the 2 day assay, and LC_50_ reflects the molar concentration required to kill 50% of the cells that were present at the time of drug addition (Holbeck, 2004). Values are calculated for each of these three parameters if the level of activity is reached; however, if the effect is not reached or is exceeded, the value for that parameter is expressed as greater or less than the maximum or minimum concentration tested (Doroshow et al., 2012).

#### Clustered image map

The heat map was generated using CIMminer (http://discover.nci.nih.gov/cimminer). Hierarchical clustering of the GI_50_ activity patterns was done using the Euclidian distance method and the average linkage cluster algorithm.

#### CellMiner^TM^

Analysis of the GI_50_ data from the NCI-60 cell line screening for the compounds **5**, **12**, and **17** was performed using the publicly accessible web tool CellMiner^TM^ (http://discover.nci.nih.gov/cellminer/).

#### Cell culture and antibodies

Colo205, SK-Mel-28, Malme-3M and A549 cells were obtained directly from the NCI Developmental Therapeutics Program (https://dtp.cancer.gov/organization/btb/docs/DCTDTumorRepositoryCatalog.pdf) and HeLa cells were purchased from ATCC HeLa (ATCC® CCL-2™). All cells were grown in RPMI-1640 supplemented with 10% fetal bovine serum (FBS) and 2 mM L-glutamine. Culture conditions were maintained at 37°C in a humidified atmosphere containing 5% CO_2_. Compounds **5**, **12** and **17**, and Raf inhibitor SB-590885 were added to culture medium dissolved in DMSO (final concentration in the assays was 0.1% v/v); controls received vehicle only. Antibodies to B-Raf, C-Raf, and ERK2 were from Santa Cruz Biotechnology; antibodies to pS217/221-MEK and pT202/Y204-ERK were from Cell Signaling Technologies; antibodies to pERK were from Sigma-Aldrich, and antibodies to MEK1 were from BD Biosciences.

#### Cell lysis

Cells were washed twice with ice-cold phosphate buffered saline 1X (PBS-1X) and lysed under stringent conditions using radioimmunoprecipitation assay (RIPA) buffer (20 mM Tris [pH 8.0], 137 mM NaCl, 10% glycerol, 1% NP-40, 0.5% sodium deoxycholate, 0.1% SDS, 0.15 U/mL aprotinin, 1 mM phenylmethylsulfonyl fluoride, 0.5 mM sodium vanadate, 20 μM leupeptin). Lysates were clarified by centrifugation and equalized for protein content, prior to analysis by SDS-polyacrylamide gel electrophoresis (SDS-PAGE) and immunoblotting.

#### *In vitro* kinase assays

To monitor the effect of the compounds on Raf kinase activity, purified kinase-active Raf proteins were added to 10 μL 30 mM Tris [pH 7.4] containing 10 μM of the indicated compound/drug and incubated at room temperature for 20 min, prior to the addition of 40 μL kinase buffer (30 mM Tris [pH 7.4], 1 mM DTT, 10 mM MgCl_2_, 5 mM MnCl_2_, 1 mM ATP) containing 20 μCi of [γ^32^P]ATP and 0.1 μg kinase-inactive MEK. To evaluate the effect on MEK1 kinase activity, purified WT MEK1 proteins were incubated with the compounds/drugs as indicated above, prior to the addition of 40 μL kinase buffer containing 20 μCi of [γ^32^P]ATP and 0.1 μg kinase-inactive ERK2. All kinase reactions were incubated at 30°C for 30 min, following which the assays were terminated by the addition of gel sample buffer (250 mM Tris [pH 6.8], 50 mM DTT, 10% SDS, 30% glycerol). The samples were then analyzed by SDS-PAGE and autoradiography.

## Results and discussion

### Chemistry

#### Design and synthesis of MEA derivatives

As shown in Figure 1, structural modifications were carried out on the C-2, C-3, C-6, and C-23 hydroxy groups and the C-28 carboxylic acid of **MEA** (**1**) in order to improve its anticancer potential and establish structure-activity relationships (SARs). We also investigated the effects of converting the 6-membered ring A of **MEA** (**1**) into a 5-membered ring with an α,β-unsaturated aldehyde substituent. It has been reported that the presence of an electrophilic Michael acceptor in the A-ring of pentacyclic triterpenoids significantly enhances their anti-inflammatory and cytoprotective activities (Honda et al., 1998; Sporn et al., 2011; Salvador et al., 2012, 2017). Thus we developed a series of new MEA derivatives with either a 6-membered A-ring or an aldehyde substituted cyclopentene A-ring, along with variously functionalized ring B moieties and different substituents at C-23 in an attempt to improve the compounds' cytotoxicity and selectivity profiles.

**Scheme 1:**
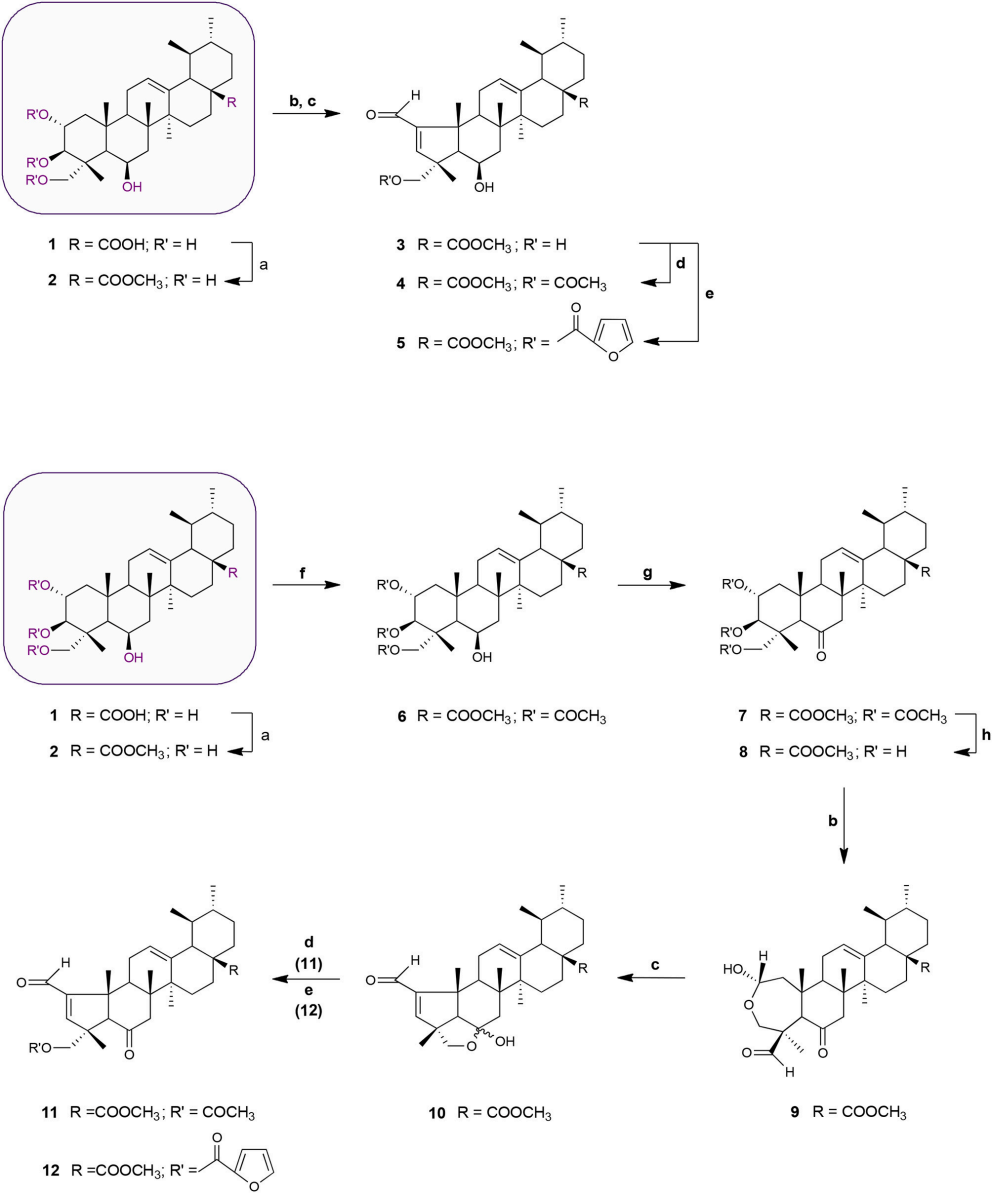
Synthesis of MEA derivatives 2-12. *Reagents and conditions*: (a) CH_3_I, K_2_CO_3_, DMF, r.t., 1 h, 82%; (b) NaIO_4_, MeOH/H_2_O 20:1, v/v, r.t., 2 h, **9**: 78%; (c) glacial acetic acid, piperidine, anhydrous MgSO_4_, dry benzene, 60°C, N_2_, **3**: 2 h, 77%; **10**: 3 h, 69%; (d) 3 eq. acetic anhydride, DMAP, dry THF, r.t., **4**: 2 h, 66%; **11**: 1 h 30 min, 61%; (e) 2-furoyl chloride, DMAP, dry benzene, 60°C, N_2_, **5**: 2 h, 65%; **12**: 2 h, 56%; (f) 8 eq. acetic anhydride, DMAP, dry THF, r.t., 1 h 30 min, 68%; (g) Jones reagent (CrO_3_ in aqueous H_2_SO_4_), acetone, 0°C, 30 min to r.t, 45 min, 75%; (h) KOH, MeOH, 65°C, 2 h, 65%.

**Scheme 2:**
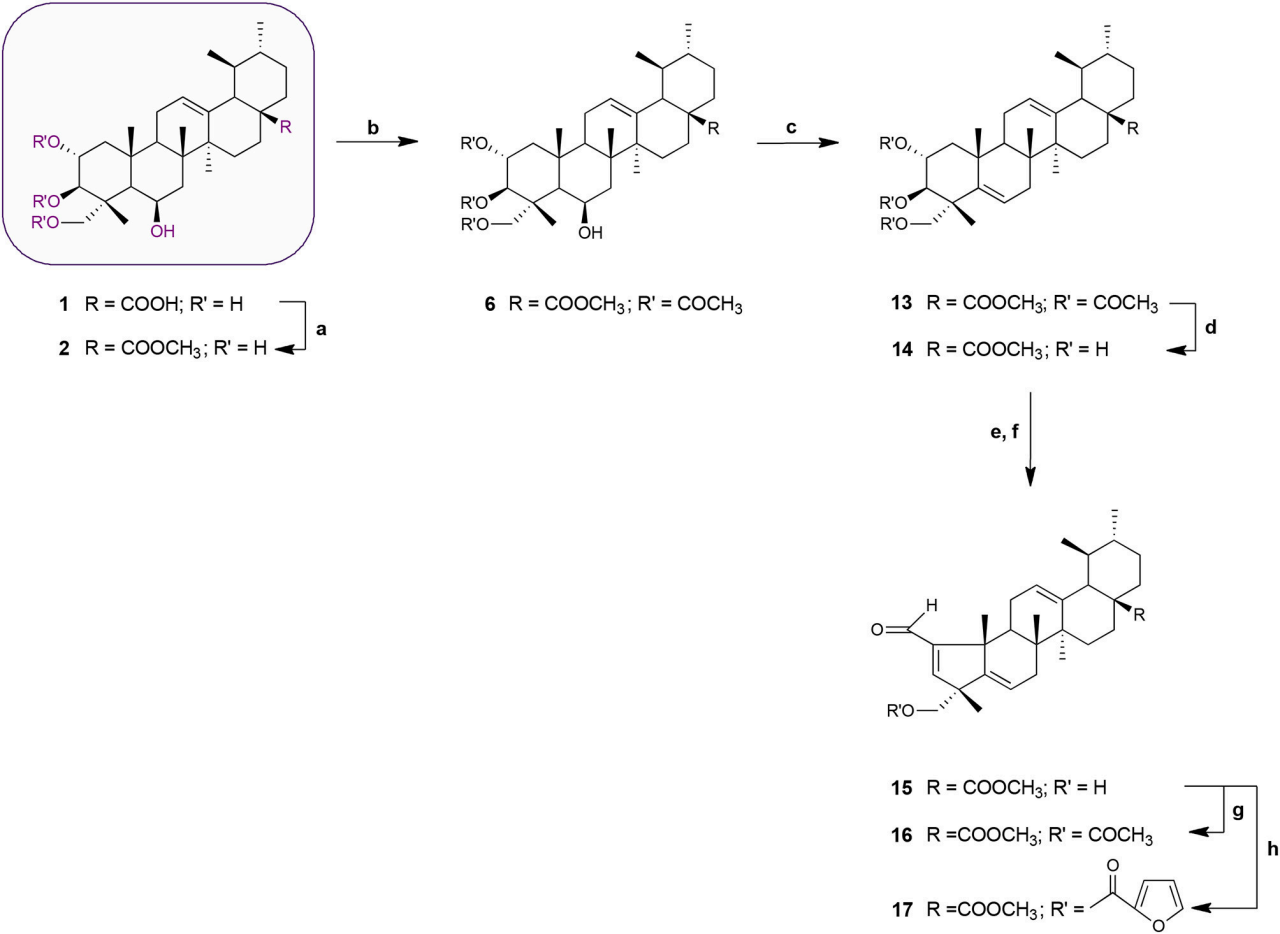
Synthesis of MEA derivatives 13–17. *Reagents and conditions*: (a) CH_3_I, K_2_CO_3_, DMF, r.t., 1 h, 82%; (b) 3 eq. acetic anhydride, DMAP, dry THF, r.t.,1 h 30 min, 62%; (c) thionyl chloride, py, r.t., 1 h 30 min, 75%; (d) KOH, MeOH, 65°C, 2 h, 84%; (e) NaIO_4_, MeOH/H_2_O 20:1, v/v, r.t., 2 h; (f) glacial acetic acid, piperidine, anhydrous MgSO_4_, dry benzene, 60°C, N_2_, 2 h 30 min, 65%; (g) 3 eq. acetic anhydride, DMAP, dry THF, r.t.,1 h 30 min, 62%; (h) 2-furoyl chloride, DMAP, dry benzene, 60°C, N_2_, 1 h 30 min, 64%.

Schemes 1 and 2 outline the synthetic pathway used to obtain 16 MEA analogs (**2-17**). The first MEA derivative was prepared by treatment of **MEA** (**1**) with anhydrous potassium carbonate (K_2_CO_3_) and methyl iodide in DMF to afford the methyl ester **2** that was used as starting material for the following reactions. This chemical modification was designed to increase the lipophilicity of the parent compound and enhance its membrane permeability. Conversion of the 6-membered ring A in **2** into a 5-membered ring with an α,β-unsaturated aldehyde **3** was achieved by treatment of this compound with sodium periodate (NaIO_4_) in methanol/water at room temperature, followed by reaction of the resulting product with catalytic amounts of piperidine and acetic acid in dry benzene at 60°C under a nitrogen atmosphere. The successful preparation of this compound was confirmed by spectral data. The ^1^H NMR showed two singlet signals at 9.71 and 6.61 ppm assigned to the aldehydic proton and to the C-3 olefinic proton, respectively. In addition, an olefinic carbon signal was evident in the ^13^C NMR spectrum at 158.7 ppm (assigned to C-3) along with a carbonyl carbon signal at 190.8 ppm. The characteristic IR band for the C = O stretching vibration of the α,β-unsaturated aldehyde was observed at 1684 cm^−1^.

Acetylation of the three hydroxyl groups of **2** with acetic anhydride in the presence of DMAP in THF at room temperature, afforded intermediate **6**. Conversion of the triacetate **6** into its keto derivative **7** was readily and quantitatively achieved via Jones oxidation. Compound **7** was then deacetylated with potassium hydroxide (KOH) in methanol to afford compound **8**. For the preparation of its dehydrated-counterpart **14**, compound **6** was treated with thionyl chloride and pyridine to give alkene **13**, which was in turn subjected to alkaline deprotection to give the corresponding triol **14**.

The A-nor MEA derivatives **10** and **15** were prepared from compound **8** and **14**, respectively, according to the same procedure as described previously for the preparation of **3**. The NMR spectra of these compounds closely resembled those of compound **3**, with changes focused on signals associated with the B-ring. The most obvious differences were attributed to the presence of an additional quaternary carbon peak assigned to C-6 at 107.3 ppm in **10**, and the presence of an additional olefinic signal at 5.58 ppm which was assigned to the C-6 proton in **15**.

Finally, we investigated the influence of C-23 hydroxyl substitution on the anticancer activity of compounds **3**, **10**, and **15**. Starting with these three compounds, we prepared a panel of C-23 substituted acetyl and furoyl esters. Introduction of these two chemically distinct groups provided new insight about the steric, hydrophobic, and electronic requirements that convey or fine-tune desirable cytotoxic properties such as increased potency, selective cytotoxicity, lipophilicity, and increased cellular uptake of these compounds. Additionally, it has been reported that derivatives of furan substituted at the 2-position display a broad-spectrum of pharmacological properties, including anticancer activity (Dong et al., 2009; Cui et al., 2010; Selvam et al., 2012). As shown in Scheme 1, compound **3** was treated with acetic anhydride in the presence of DMAP in THF at room temperature to afford the respective 23-acetate derivative **4**. The 23-(2-furoyl) ester derivative **5** was obtained by reaction of compound **3** with 2-furoyl chloride in the presence of DMAP and dry benzene at 60°C under a nitrogen atmosphere. The 23-acetyloxy and 23-(2-furoyloxy) MEA analogs **11**, **12**, **16**, and **17** were accessed by analogous reactions. In the ^1^H NMR spectra of **4**, **11**, and **16** the signals due to the acetate protons appeared at 2.06, 2.01, and 1.99 ppm and showed strong HMBC correlations with the oxymethine carbons at 69.8, 67.9, and 69.7 ppm, respectively. The introduction of a furan ring in derivatives **5**, **12**, and **17** was confirmed by the presence of three aromatic peaks at around 7.54–7.58 (H-5′), 7.08–7.17 (H-3′) and 6.46–6.52 (H-4′) ppm.

The synthetic procedures are described in detail in the Materials and Methods section. The structures of all newly synthetized compounds were confirmed by comprehensive evaluation of ^1^H-NMR, ^13^C-NMR, 2D-NMR correlations (HSQC, HMBC, and in some cases COSY and NOESY), infrared (IR) and mass spectrometry (LRMS and HRMS) data.

### Biology

#### NCI-60 anticancer drug screening

Madecassic acid and the novel semi-synthetic derivatives shown in Schemes 1 and 2 were submitted to the National Cancer Institute (NCI) for evaluation of their anticancer activity against 60 human tumor cell lines. Fourteen compounds were selected for initial evaluation according to the NCI protocols (Shoemaker, 2006; Holbeck et al., 2010) and assigned unique NSC codes viz; **1**/NSC 783350, **2**/NSC 787805, **3**/NSC 784255, **4**/NSC 784258, **5**/NSC 783356, **6**/NSC 787221, **9**/NSC 783355, **10**/NSC 785397, **11**/NSC 784261, **12**/NSC 784260, **14**/NSC 787216, **15**/NSC 785391, **16**/NSC 784264, and **17**/NSC 784263.

For the initial screening, compounds were assayed at a single concentration of 10 μM in the full NCI-60 cancer cell line panel. Test compounds were administered to the cell lines and after 48 h the percent growth of treated cells was determined relative to cells without drug treatment, and to the number of cells at time zero. This allows the detection of both growth inhibition (values between 0 and 100) and lethality (values less than 0). Results of the initial single dose (10 μM) testing for all 14 compounds against this panel of 60 human tumor cell lines are presented in the Supplementary Material (Figures S1–S14). Only compounds that showed ≥60% growth inhibition in at least eight tumor cell lines were selected for further dose-response testing and the others were deemed inactive (Malhotra et al., 2014).

Nine of the MEA derivatives (**2**, **3**, **4**, **5**, **6**, **12**, **14**, **16**, and **17**) satisfied the NCI inhibition criteria and were selected for five concentration dose-response testing (0.01, 0.1, 1, 10, 100 μM). Results from the 60-cell screen are displayed in the Supplementary Material as dose-response curves (% growth vs. sample concentration) for each cell line in the nine cancer subpanels (Figures S15A–S23A). The antitumor activity parameters GI_50_ and LC_50_ are calculated from the dose-response curves by linear interpolation. TGI is determined as the x-axis intercept. Mean graphs were constructed by using a vertical line that represents the mean response of all cell lines in the assay and horizontally plotting positive or negative values relative to the mean for each individual cell line. Projections to the right indicate cell lines with susceptibility that exceeds the mean (more sensitive), projections to the left indicate cell lines with lower susceptibility (more resistant) (Doroshow et al., 2012). The mean graphs obtained for each compound are shown in the Supplementary Material (Figures S15B–S23B).

A comparative summary of the mean GI_50_, TGI, and LC_50_ values for the nine compounds tested across the entire NCI-60 cell line panel is shown in Table 1.

**Table 1:** Selected MEA derivatives were assessed in the NCI-60 assay at five different doses, ranging from 10 nM to 100 μM.

**Compounds**	**NSC number^a^**	**Mean GI_50_ (μM)**	**Mean TGI (μM)**	**Mean LC_50_ (μM)**
**2**	787805	12.88	31.62	74.13
**3**	784255	3.98	14.45	50.12
**4**	784258	2.19	6.03	27.54
**5**	783356	1.55	6.31	47.71
**6**	787221	1.70	64.57	95.50
**12**	784260	2.95	42.66	87.10
**14**	787216	16.98	33.88	67.61
**16**	784264	4.27	16.22	56.23
**17**	784263	1.48	8.32	50.12

a *National Service Center number assigned by the Developmental Therapeutics Program to compounds tested in the NCI-60*.

Based on the data obtained from the anticancer screening studies, SAR correlations were determined. According to previous studies,(Tu et al., 2009; Siewert et al., 2013; Goncalves et al., 2016) the presence of a short alkyl ester chain (up to a maximum length of six carbons) at position C-28 has been proved to increase the cytotoxicity of ursane-type triterpenoids. Similar results were obtained in our study, the MEA methyl ester derivative **2** exhibited increased growth inhibition activity compared with the parent compound (**MEA**, **1**), which was deemed inactive in the one-dose 60-cell assay. A marked improvement in the cell growth inhibition was also achieved when acetyl groups were introduced to the C_2_-OH, C_3_-OH and C_23_-OH positions (**6**), resulting in a compound 7.5-fold more active than its precursor **2**.

With regard to functionalization on the B-ring, the presence of a hydroxyl group at C-6 or a C_5_-C_6_ double bound led to compounds with comparable potencies (compare **14** with **2**, Table 1), suggesting that the hydroxyl group at C-6 is not essential for cytostatic activity. Interestingly, the antiproliferative activity of pentameric A ring derivatives containing an α,β-unsaturated carbonyl moiety seems to be dependent on the B-ring functionalization. In fact, compound **3** bearing a hydroxyl group at C-6 was found to be more potent than the hydroxy ether derivative **10** and its dehydrated-counterpart **15**, which were deemed inactive in the one-dose 60-cell assay, and 3-fold more potent than its precursor **2**.

Within the pentameric A-ring C-23 functionalized series, with exception of compound **11**, all other compounds showed promising results in single-dose testing and were selected for further evaluation against the 60 cell panel at five concentration levels. SAR analysis revealed enhanced potencies of these derivatives compared to their parent compound. Notably, a significant cytostatic activity against the diverse cancer cell types was evident for the furoyl-containing compounds **5** and **17** with mean GI_50_ and TGI values in the low micromolar range (**5**: mean GI_50_ = 1.55 μM and TGI = 6.31 μM; **17**: mean GI_50_ = 1.48 μM and TGI = 8.32 μM, Table 1).

**Figure 2:**
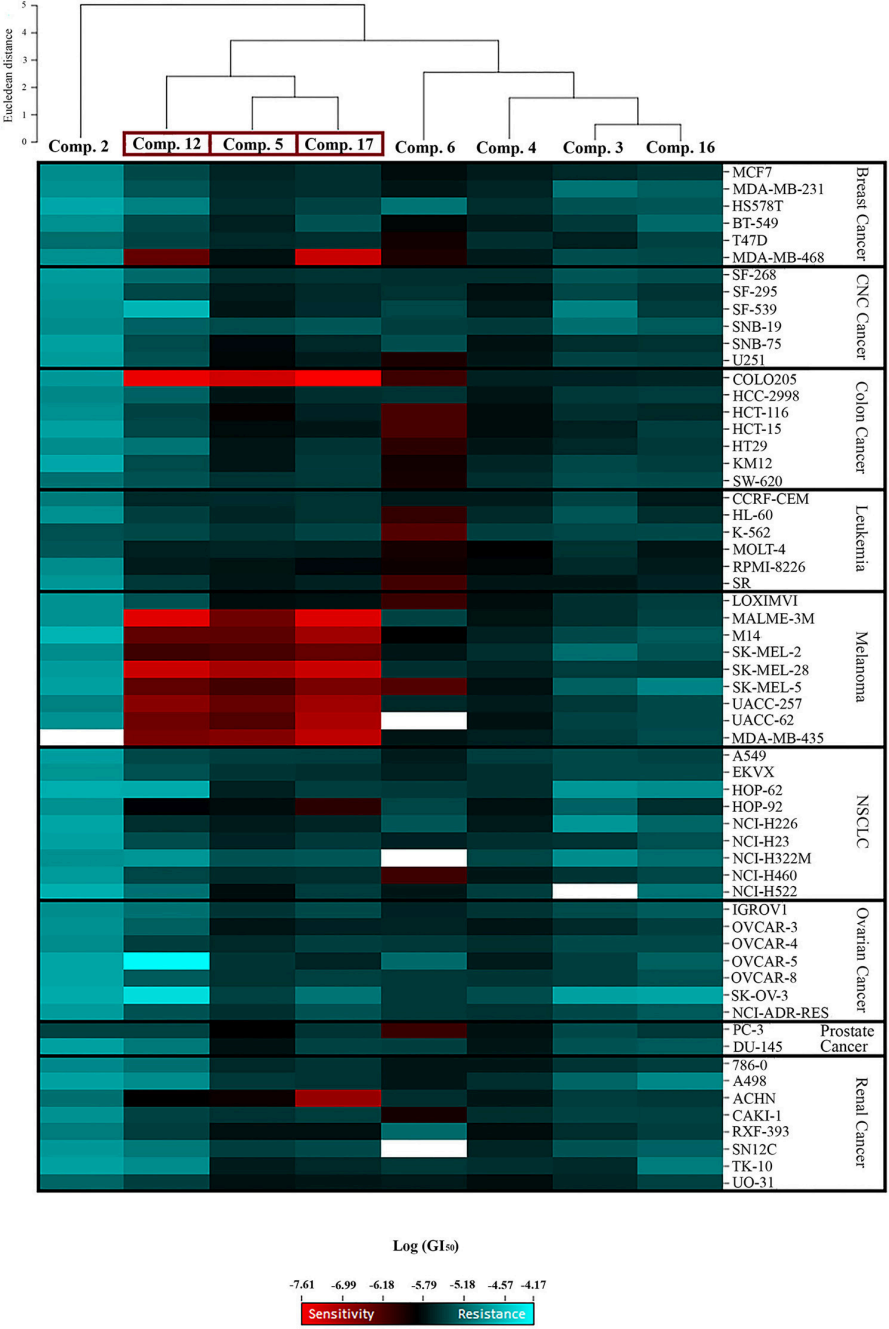
Heat map showing the log (GI_50_) for compounds **2**, **3**, **4**, **5**, **6**, **12**, **16**, and **17** in the NCI-60 screen. The color gradient ranges from light red (higher activity) to light blue (lower activity). Empty cells correlate with no data available for that specific cell line. Hierarchical clustering of GI_50_ activity patterns was done using the Euclidian distance method and the average linkage cluster algorithm.

Significant cytostatic activity against the diverse cancer cell types was evident for these compounds with mean GI_50_ (1.48–16.98 μM) and TGI (6.03–64.57 μM) values in the low micromolar range. While these mean values reflect the overall potency of an agent, they do not reflect cell line specificity or tumor panel-average sensitivity to a given compound. A more meaningful approach is to assess how individual cell lines in the entire 60-cell panel respond to treatment with the test compound, and then compare that response to the pattern of cell line sensitivity or resistance seen with other agents tested in the 60-cell screen. For this purpose, a heat map (Figure 2) that provides an immediate visual summary of the patterns of growth inhibition for each compound evaluated in the NCI-60 panel was generated. The growth inhibitory activity of tested compounds is expressed in terms of log(GI_50_); light red color cells correspond to the highest activity (lower GI_50_ values), whereas light blue color cells represent the lowest activity (higher GI_50_ values), for each compound.

The most striking observation is the level of similarity in the selectivity patterns between the three compounds **5**, **12**, and **17**. These compounds showed significant growth inhibitory activity at nanomolar concentrations in one colon (Colo205) and eight melanoma (Malme-3M, M14, SK-Mel-2, SK-Mel-28, SK-Mel-5, UACC-257, UACC-62, and MDA-MB-435) cell lines. The mean GI_50_'s for these compounds across the eight melanoma cell lines were calculated and the most potent was compound **17** (mean GI_50_ = 94.68 nM), followed by **12** (mean GI_50_ = 165.01 nM), and **5** (mean GI_50_ = 257.78 nM). The selectivity ratio (SR) of these compounds was obtained by dividing the mean GI_50_ of the full 60-cell panel by the mean GI_50_ of the melanoma subpanel. Ratios between 3 and 6 refer to moderate selectivity, ratios greater than 6 indicate high selectivity toward the corresponding subpanel, while compounds not meeting either of these criteria are rated non-selective (Rostom, 2006). In this context, compound **12** proved to be the most selective toward the melanoma cell lines with a selectivity ratio of 12.29. Compound **17** also exhibited high selectivity (SR = 11.38), while compound **5** showed more modest selectivity toward the melanoma subpanel (SR = 4.84). All eight test compounds were hierarchically clustered based on their in vitro activity patterns across all 60 cell lines (Figure 2). The cluster tree indicates a distinct separation of the compounds into two major subgroups. Compounds **5**, **12**, and **17** clustered side by side on a single branch, indicating that they have similar activity patterns, which is consistent with these observations.

**Figure 3:**
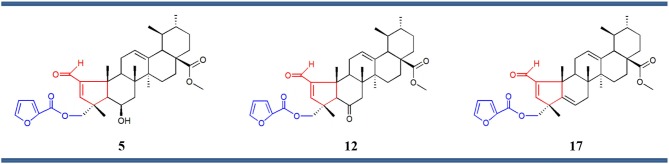
Chemical structures of compounds **5**, **12**, and **17** comprising the same chemical structural feature: a 5-membered A ring containing an α,β-unsaturated carbonyl group (red) substituted at the position C-23 with a 2-furoyl group (blue).

Structure–activity relationship (SAR) analysis showed that these three compounds share distinctive structural features: a 5-membered A ring substituted with an α,β-unsaturated aldehyde group, and a 2-furoyl group appended at position C-23. These moieties appear to be crucial for MEA analogs to produce this selective pattern of growth inhibition. Further, B-ring functionalization also impacts the activity and selectivity of these compounds. Compound **17**, which bears a C5-C6 double bound, displayed the most potent growth inhibitory activities against the eight sensitive melanoma cell lines (Malme-3M, M14, SK-Mel-2, SK-Mel-28, SK-Mel-5, UACC-257, UACC-62, and MDA-MB-435) and the one colon cell line (Colo205), with GI_50_ values ranging from 24.55 to 275.42 nM. Derivative **12** was slightly less potent but it showed the highest selectivity toward the melanoma subpanel. The C-6 hydroxy derivative (**5**) was the least potent and least selective of the three, indicating that chemical modification of the original hydroxyl group at C-6 (B ring) led to new compounds with increased activity and selectivity (Figure 3).

#### CellMiner^TM^analysis

The value of using CellMiner^TM^ to identify a compound's target or mechanism of action has been validated by several notable successes (Reinhold et al., 2012, 2014; Varma et al., 2014). In brief, it correlates the similarity of the mean bar graph cytotoxicity profile in the 60-cell line panel of antitumor agents having a known mechanism of action with that generated by a new test compound with an unknown mechanism of action. In this way it is possible to identify compounds whose cytotoxicity profiles are most similar to those of the “seed” and therefore these agents putatively share the same mechanism of action. The results are quantitated using Pearson's correlation coefficient (*r*) as a measure of similarity: a correlation of 1.0 identifies a perfect match, *r* of −1.0 denotes a perfect mirror image, while *r* of 0 means there is no correlation between the two patterns (Holbeck et al., 2010). Using this approach, we found that the three MEA derivatives highly correlated (*r* ≥ 0.79) with two FDA-approved B-Raf inhibitor drugs, dabrafenib (NSC 763760) and vemurafenib (NSC 761431), that ranked second and third, respectively, among the 20,503 drugs in CellMiner^TM^ (Table 2). These drugs received FDA approval as kinase inhibitors indicated for the treatment of patients with B-Raf^V600E^ mutation-positive metastatic melanoma (Bollag et al., 2012; Robert et al., 2015). Interestingly, CellMiner^TM^ analysis also showed a pattern similarity between our newly synthesized compounds and two other drugs, cobimetinib (NSC 768069) and hypothemycin (NSC 354462). Cobimetinib is a MEK inhibitor, recently approved by the FDA for treatment of patients with B-Raf^V600E^ mutation-positive melanoma in combination with vemurafenib (Garnock-Jones, 2015). Hypothemycin is a resorcylic acid lactone that has been reported to selectively and irreversibly inhibit protein kinases that contain a conserved cysteine residue (Cys^166^) in the ATP-binding site, including the mitogen-activated protein kinase (MEK) and the extracellular signal-regulated kinase (ERK) (Fukazawa et al., 2010). These CellMiner^TM^ results suggested that the new MEA analogs might impact signal transduction through the ERK cascade.

**Table 2:** CellMiner^TM^ results and possible mechanism of action for the compounds **5**, **12**, and **17**.

	**CellMiner**^**TM**^ **results**				
	**NSC^#^^a^**	**Name**	**MOA^b^**	**r value^c^**	**FDA^d^ status**
**Comp. 5**	706829	1,6-bis[4-(4-aminophenoxy)phenyl]diamantine	-	0.89	
	763760	**Dabrafenib**	B-Raf inhibitor	0.83	Approved
	761431	**Vemurafenib**	B-Raf inhibitor	0.81	Approved
	656082	**-**	-	0.80	-
	768069	Cobimetinib	MEK inhibitor	0.67	Clinical trial (phase III)
	354462	Hypothemycin	MEK inhibitor	0.67	Clinical trial (phase I)
	715767	-	-	0.65	-
**Comp. 12**	706829	1,6-bis[4-(4-aminophenoxy)phenyl]diamantine	-	0.88	-
	763760	**Dabrafenib**	B-Raf inhibitor	0.79	Approved
	761431	**Vemurafenib**	B-Raf inhibitor	0.79	Approved
	656082	**-**	-	0.76	-
	354462	Hypothemycin	MEK inhibitor	0.72	Clinical trial (phase I)
	617644	-	-	0.67	-
	170992	-	-	0.67	-
**Comp. 17**	706829	1,6-bis[4-(4-aminophenoxy)phenyl]diamantine	-	0.89	-
	763760	**Dabrafenib**	B-Raf inhibitor	0.86	Approved
	761431	**Vemurafenib**	B-Raf inhibitor	0.82	Approved
	656082	**-**	-	0.80	-
	768069	Cobimetinib	MEK inhibitor	0.71	Clinical trial (phase III)
	354462	Hypothemycin	MEK inhibitor	0.69	Clinical trial (phase I)
	765695	PD 184352	MEK inhibitor	0.67	-

*Compounds with most similar patterns of cytotoxicity (r ≥ 0.65) are reported*.

a *National Service Center number assigned by the Developmental Therapeutics Program to compounds tested in the NCI-60*.

b *Mechanism of action*.

c *Pearson's correlation coefficient*.

d *Food and Drug Administration*.

*FDA-approved drugs are highlighted in bold*.

Comparison of the NCI-60 mean bar graphs of the three top-ranking drugs identified by CellMiner^TM^ (NSC 706829, NSC 763760, and NSC 761431) with the mean graphs of our three selective MEA congeners (**5**, **12**, and **17**) revealed that all of these compounds share similar patterns of growth inhibition for the colon and melanoma cell lines (Figure S24). The most highly correlated agent (*r* = 0.89) in CellMiner^TM^ was a diamantane derivative, 1,6-bis[4-(4-aminophenoxy)phenyl] diamantane (NSC 706829). There are no reports in the literature concerning its mechanism of action and we were unable to obtain this compound from any commercial sources or from the NCI compound repository to do comparative studies with the MEA compounds.

Interestingly, genomic analyses performed in a previous study (Ikediobi et al., 2006) revealed that 10 out of the 60 tumor cell lines comprising the NCI screening panel harbor the V600E mutation in the *b-raf* gene (Table 3). The more potent mean GI_50_ values of the MEA compounds observed with the majority of these cells lines vs. those that lack this mutation, suggests that these molecules preferentially inhibit growth of cells harboring this particular mutation. These observations are consistent with CellMiner^TM^ results. However, the HT29 colon cancer cell line, which also harbors the B-Raf^V600E^ mutation, was not particularly sensitive to treatment with the MEA compounds. HT29 cells have also shown resistance to the B-Raf^V600E^ selective inhibitor PLX4720 as previously reported (Oikonomou et al., 2011; Temraz et al., 2015). In a similar fashion, the melanoma line LOXIMVI was relatively insensitive to vemurafenib and the three MEA analogs, although it does carry the V600E mutation in its *b-raf* gene (Figure S24).

**Table 3:** Cells bearing the B-Raf^V600E^ mutation in the NCI-60 cell lines.

**Cell Line**	**Tissue**
Colo205	Colon
HT29	Colon
LOXIMVI	Melanoma
Malme-3M	Melanoma
M14	Melanoma
SK-Mel-28	Melanoma
SK-Mel-5	Melanoma
UACC-257	Melanoma
UACC-62	Melanoma
MDA-MB-435	Melanoma

Collectively, these results suggest that the novel chemical scaffold and nanomolar antiproliferative activity of these small molecules for the specific B-Raf^V600E^ colon and melanoma cell lines could provide a chemotherapeutic lead for the development of new anticancer drugs. Potent agents that can target B-Raf^V600E^-related signaling could be effective anti-proliferative agents against many cancers that carry this mutation. Although CellMiner^TM^ is a valuable source to predict possible molecular targets and mechanism of actions, it is important to emphasize that these hypotheses need to be verified experimentally.

#### Mechanistic *in vitro* studies of compounds 5, 12, and 17 in B-RafV600E mutation-positive cell lines

The V600E mutation, which involves the substitution at amino acid position 600 from a valine (V) to a glutamic acid (E) within the activation segment of the B-Raf kinase domain, represents the vast majority of all B-Raf mutations in cancer. This alteration results in a ~500-fold increase in kinase activity compared to wild-type B-Raf and allows B-Raf^V600E^ to stimulate the MEK–ERK signaling pathway without any input from upstream effectors or external stimuli (El-Nassan, 2014; Strickler et al., 2017). In contrast to the wild-type protein and various kinase-impaired mutants, B-Raf^V600E^ is not dependent on dimerization and therefore it is able to bypass the inhibitory effects of negative-feedback regulation by ERK, leading to constitutive activation of MEK–ERK signaling and uncontrolled cell growth and survival (Freeman et al., 2013; Rahman et al., 2013; Godoy-Gijón et al., 2017).

The unprecedented clinical efficacy of vemurafenib (NSC 761431) and dabrafenib (NSC 763760) was a resounding proof-of-concept for targeting mutant B-Raf, particularly B-Raf^V600E^, in melanoma and other carcinomas that arise from aberrant B-Raf signaling. Despite the breakthrough impact of these B-Raf inhibitors for the treatment of metastatic melanoma, these agents have important limitations. After impressive initial responses, the majority of patients treated with these agents suffer disease relapse (acquired resistance) within 6–8 months (Tentori et al., 2013; Godoy-Gijón et al., 2017). In addition, many other tumor types containing the B-Raf^V600E^ mutation, such as colorectal cancer, are associated with an unfavorable prognosis and fail to respond to these B-Raf inhibitors (intrinsic resistance) (Sullivan and Flaherty, 2013; Uehling and Harris, 2015). Indeed, patients with metastatic colorectal cancer harboring B-Raf^V600E^ mutations have ~70% higher mortality when compared to tumors possessing wild-type B-Raf (Corcoran et al., 2012; Prahallad et al., 2012; Strickler et al., 2017). Finally, a variety of side effects has also been reported in patients treated with vemurafenib or dabrafenib. One notable toxicity of these drugs is the development of benign skin tumors, including keratoacanthomas and squamous cell carcinomas. These secondary tumors are thought to arise as a consequence of the paradoxical activation of ERK signaling in cells with wild-type B-Raf that harbor Ras mutations (Tentori et al., 2013). Tremendous strides in the mechanisms of resistance and comprehensive understanding of the biology of MAPK signaling provide insight into rational combination regimens and sequences of molecularly targeted therapies. Although the resistance mechanisms identified so far are diverse, most seem to rely directly upon the reactivation of the MEK–ERK signaling and enhanced signaling output through the PI3K/Akt/mTOR pathway (Fedorenko et al., 2011).

**Figure 4:**
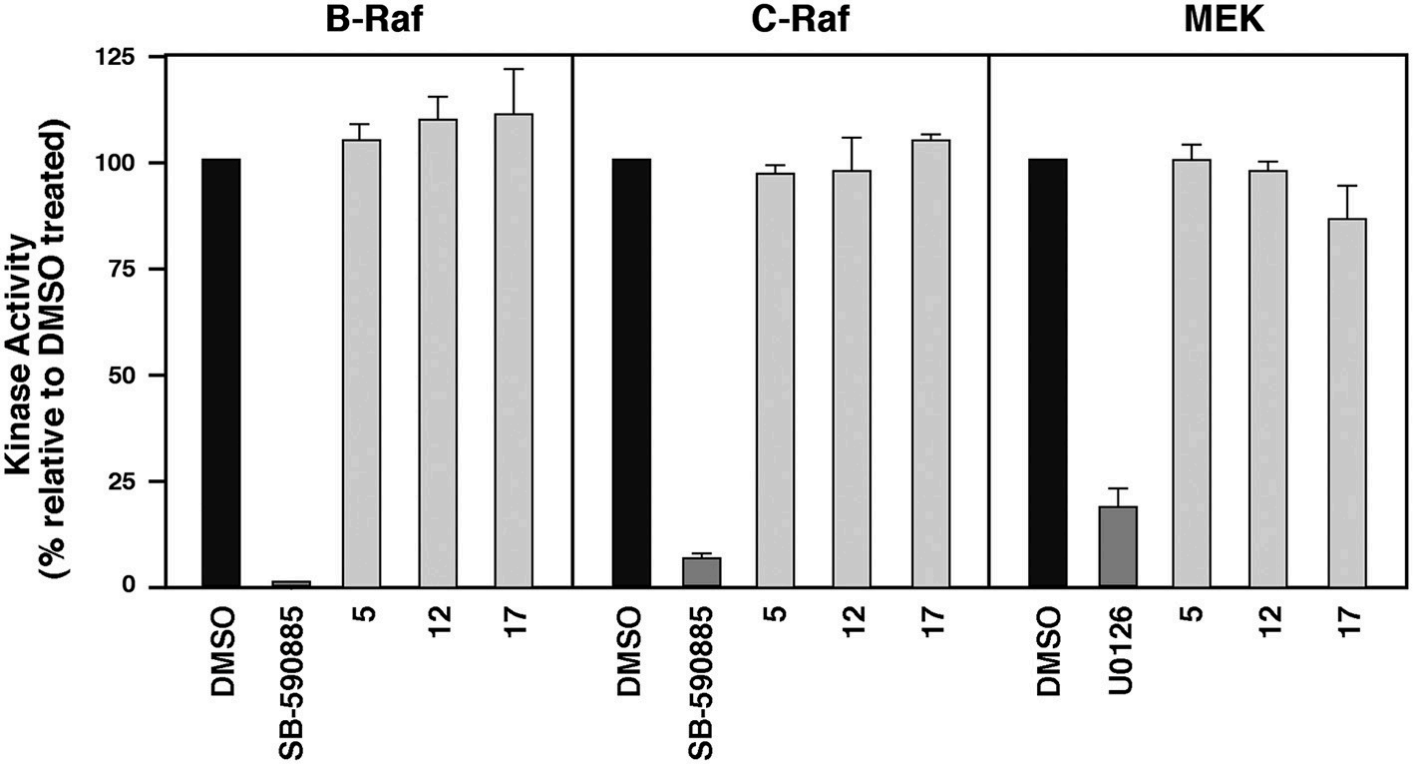
Compounds **5**, **12**, and **17** do not function as ATP-competitive Raf inhibitors. Purified B-Raf, C-Raf and MEK1 kinases were incubated with the indicated compounds for 20 min prior to determining the intrinsic catalytic activity for the respective kinases. The ATP-competitive Raf inhibitor SB-590885 and the allosteric MEK1 inhibitor U0126 were included as controls.

To evaluate whether these MEA compounds function as direct B-Raf^V600E^ inhibitors, we tested whether they inhibited the intrinsic kinase activity of the Rafs. For these experiments, 10 μM of **5**, **12**, or **17** were incubated with purified, kinase-active B-Raf or C-Raf for 20 min prior to measuring Raf catalytic activity using kinase-inactive MEK as an exogenous substrate. In comparison to the known ATP-competitive B-Raf inhibitor SB-590885, which effectively blocked both B-Raf and C-Raf kinase activity, the MEA derivatives had no significant effect on Raf catalytic activity. They were also shown to have no direct effect on the kinase activity of MEK1 (Figure 4).

**Figure 5:**
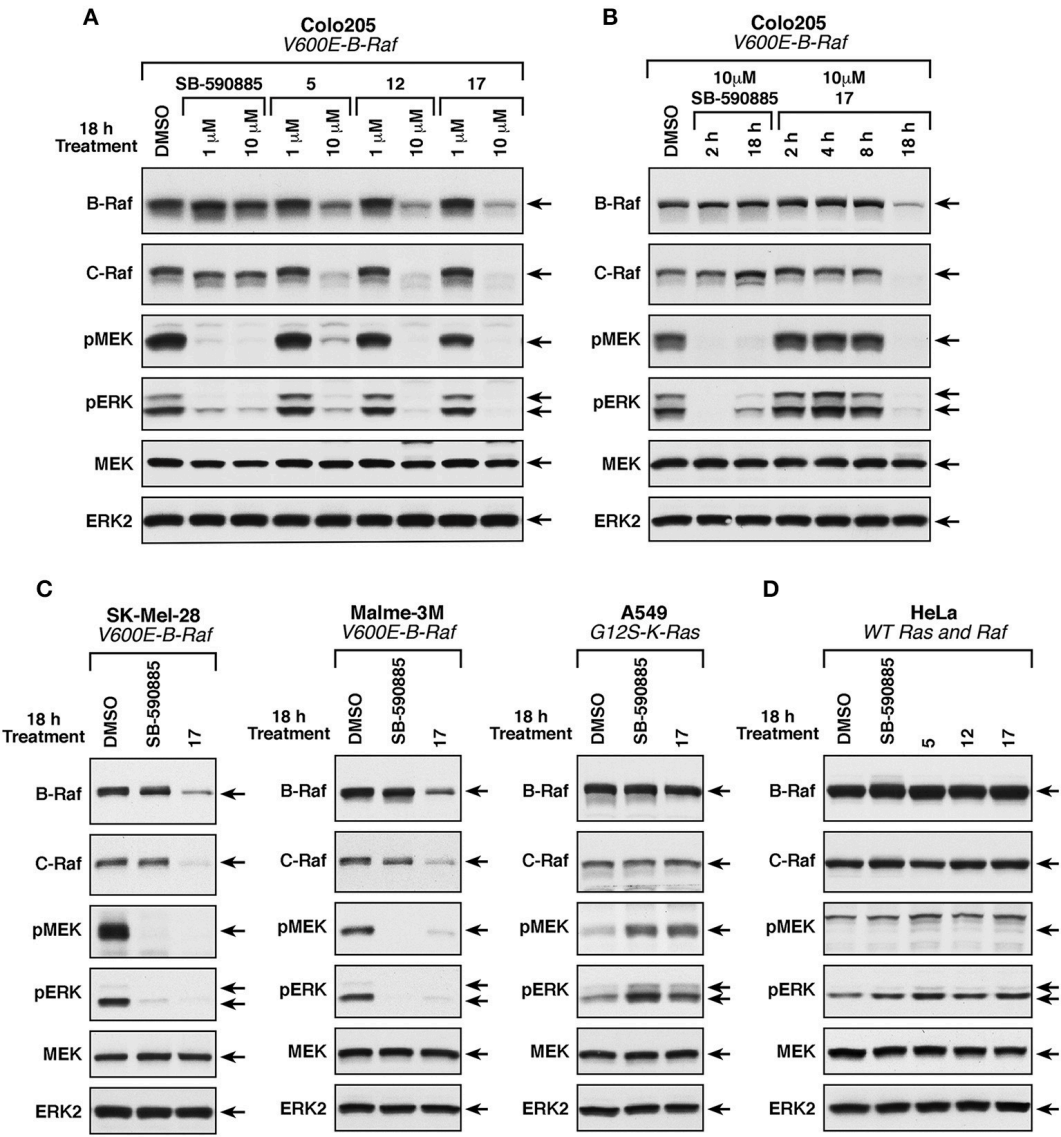
Effect of compounds **5**, **12**, and **17** on ERK cascade signaling and B-Raf and C-Raf protein levels. **(A)** B-Raf^V600E^-mutant Colo205 cells were treated with SB-590885, **5**, **12**, or **17** at the indicated concentrations for 18 h prior to cell lysis. Levels of activated phospho-MEK and phospho-ERK and total levels of endogenous B-Raf, C-Raf, MEK, and ERK were determined by immunoblot analysis. **(B)** B-Raf^V600E^-mutant Colo205 cells were treated with SB-590885 or **17** at the indicated concentrations for 2, 4, 8, and 18 h prior to cell lysis. Protein lysates were analyzed as described in **(A)**. **(C)** B-Raf^V600E^ (SK-Mel-28 and Malme-3M) and K-Ras-mutant (A549) cells were treated with 10 μM of SB-590885 or **17** for 18 h prior to cell lysis. Immunoblot analysis of protein lysates were performed as in **(A). (D)** Ras/Raf^WT^ HeLa cells were treated with 10 μM of SB-590885, **5**, **12**, or **17** for 18 h prior to cell lysis. Lysates were analyzed in a similar fashion (as described in **A**). Control cells were treated with vehicle control (0.01% DMSO).

To examine the effect of these compounds on ERK cascade signaling in intact cells, we used the Colo205 colon cancer line that harbors the B-Raf^V600E^ mutation and showed the highest sensitivity to these small molecules in growth assays. Cells were treated with either 1 or 10 μM of the MEA derivatives, or the B-Raf inhibitor SB-590885 for 18 h, following which the cells were lysed and analyzed for the presence of activated phospho-MEK and phospho-ERK, and for total B-Raf, C-Raf, MEK, and ERK protein levels (Figure 5A). As expected, given the expression of B-Raf^V600E^ in the Colo205 line, ERK cascade activation was detected in control DMSO-treated Colo205 cells and the levels of activated phospho-MEK and phospho-ERK could be dramatically reduced when cells were treated with 1 or 10 μM SB-590885. Treatment with 10 μM, but not 1 μM of **5**, **12**, or **17** also suppressed ERK cascade signaling; however, in contrast to SB-590885, cells treated with 10 μM of the MEA analogs showed a significant reduction in B-Raf and C-Raf protein levels, whereas total MEK and ERK protein levels were unaffected. Compound **17** was found to produce the largest reduction of the Raf protein levels and was selected for additional studies.

To further investigate this observation, Colo205 cells were treated with 10 μM of **17** or SB-590885 for various times before lysis and analysis. As shown in Figure 5B, the Raf inhibitor SB-590885 effectively blocked ERK cascade signaling after 2 h of treatment, whereas no effect on phospho-MEK and phospho-ERK levels was observed in cells treated with **17** for 2, 4, or 8 h. Only after 18 h of **17** treatment a block in ERK signaling cascade was observed, which again coincided with a decrease in B- and C-Raf protein levels (Figure 5B). Treatment with compound **17** for 18 h also reduced Raf protein levels and ERK cascade signaling in SK-Mel-28 and Malme-3M cells, melanoma lines that expresses B-Raf^V600E^ and were more sensitive to the growth inhibitory effect of the three MEA compounds (Figure 5C). In contrast, 18 h of **17** treatment had no significant effect on phospho-MEK, phospho-ERK, or Raf protein levels in A549 cells, which have wild-type B-Raf and are significantly less sensitive to the growth inhibitory effects of these compounds (Figure 5C). The same effect was observed for HeLa cells which also have wild-type B-Raf (Figure 5D).

Taken together, these results indicate that the mechanism of action of these key compounds involves inhibition of the ERK pathway through the reduction of B- and C-Raf protein levels. Nevertheless, our results do not exclude a potential impact of these newly synthesized compounds in other signaling pathways that may modulate ERK to regulate cell growth and in some cases tumorigenesis.

## Conclusion

In the present study, we synthetized a series of novel madecassic acid (MEA) derivatives and screened them for antitumor activity against the NCI-60 cancer cell line panel. Among the tested compounds, **5**, **12**, and **17** showed high similarity in their selectivity patterns with significant growth inhibitory activity at nanomolar concentrations for 80% of the tumor cells lines harboring the B-Raf^V600E^ mutation. Structure-activity analysis revealed that a 5-membered A ring containing an α,β-unsaturated aldehyde substituted at C-23 with a 2-furoyl group seems to be crucial to produce this particular growth inhibition signature. Follow-up analysis revealed that these compounds can effectively inhibit ERK cascade signaling in B-Raf^V600E^-mutation bearing cell lines by reducing Raf protein levels and, consequently, MEK and ERK phosphorylation without any effect on their total protein levels. In particular, **17** produced the largest reduction of Raf protein levels among the tested compounds, which is consistent with the results from the NCI-60 screening that identified **17** as the most potent compound in suppressing tumor growth of colon and melanoma B-Raf^V600E^-mutant cell lines. These encouraging results not only provide new insight into the mechanism of action of the madecassic acid derivatives, but also led to the identification of compound **17** as a potential lead for the development of new anticancer agents.

## Author contributions

AV performed the chemical synthesis under supervision of JS. NMR, IR, LRMS, and HRMS were performed by AV under supervision of KG and JS. CellMiner^TM^ analysis was performed by AV and KG. JM was responsible for the NCI-60 Human Tumor Cell Lines Screening and analysis. DR performed molecular biology and cell-based experiments under supervison of DM. The manuscript was written by AV and DR with the support of DM, KG, JS, and JM. All authors approved the manuscript in its final form for publication.

### Conflict of interest statement

The authors declare that the research was conducted in the absence of any commercial or financial relationships that could be construed as a potential conflict of interest.
