# Supplementary material for: Synthesis and Biological Evaluation of New Madecassic Acid Derivatives Targeting ERK Cascade Signaling
Source: Front Chem. 2018 Sep 28;6:434. doi: 10.3389/fchem.2018.00434 (PMC6172662; doi:10.3389/fchem.2018.00434)
Supplement: Supplementary file 1 [file Image_1.PDF]

*Supplementary Material*

# Synthesis and biological evaluation of new madecassic acid derivatives targeting ERK cascade signaling

**Ana S. C. Valdeira,[1,2] Daniel A. Ritt,[3] Deborah K. Morrison,[3,*] James B. McMahon,[4] Kirk R. Gustafson,[4,*] Jorge A. R. Salvador [1,2,*]**

*** Correspondence:**
Deborah K. Morrison
morrisod@mail.nih.gov
Kirk R. Gustafson
gustafki@mail.nih.gov
Jorge A. R. Salvador
salvador@ci.uc.pt

# Selected spectroscopic data

## 1. NMR data for compound 3

1.1 $^{1}$H-NMR spectrum for compound **3** recorded in $CDCl_3$

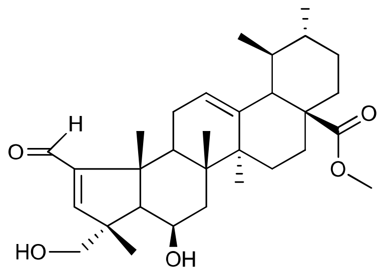


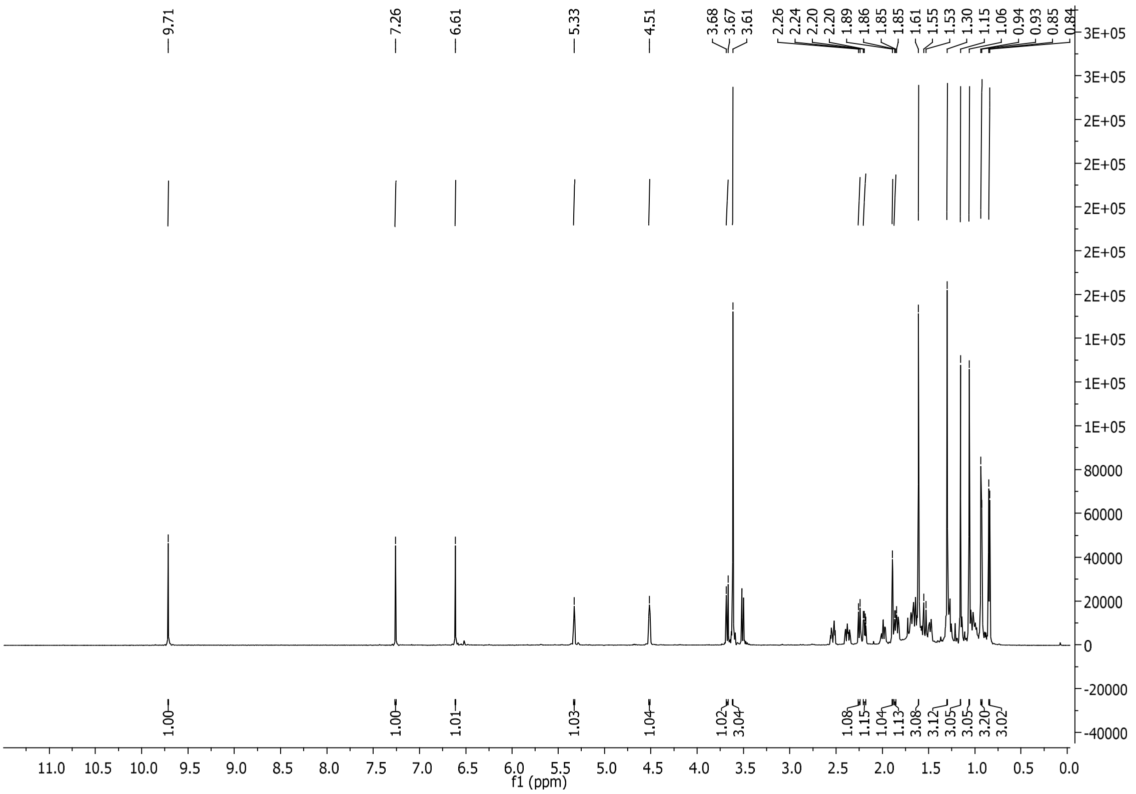

1.2 $^{13}$C-NMR spectrum for compound **3** recorded in $CDCl_3$

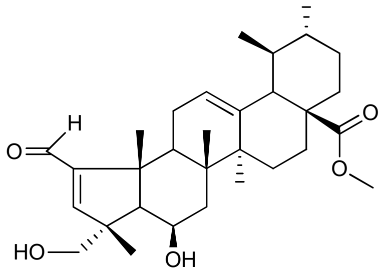

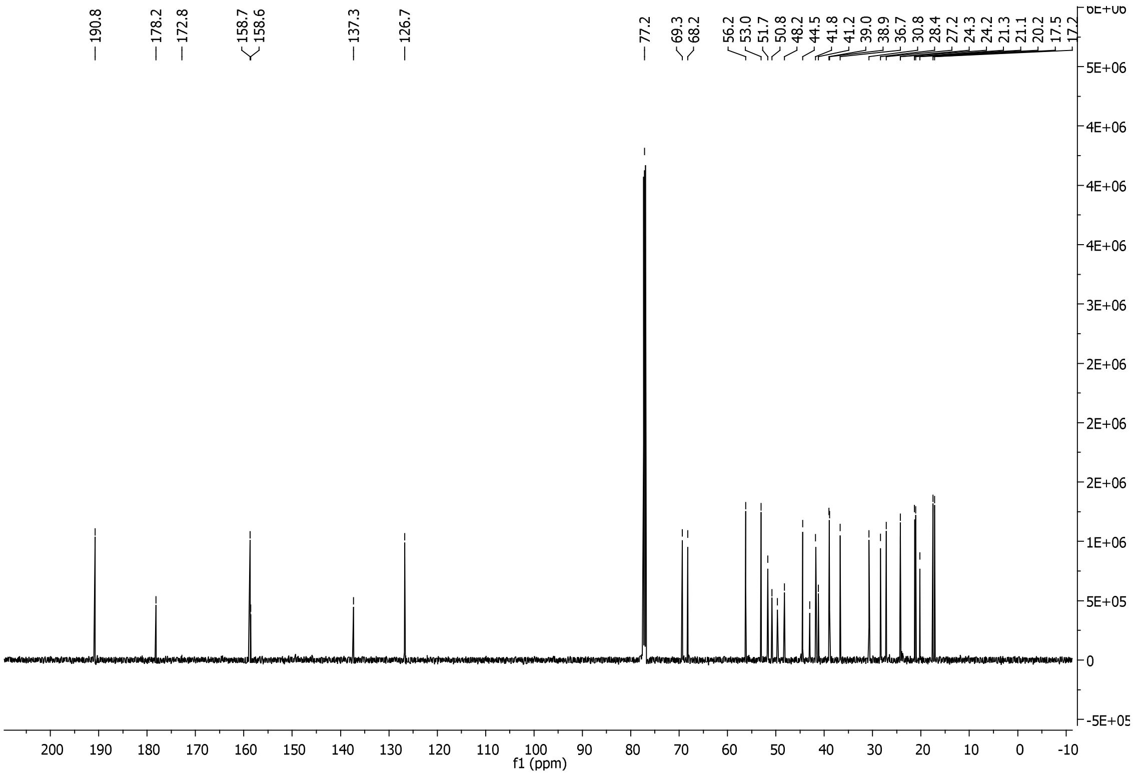

## 2. NMR data for compound 4

### 2.1 $^{1}$H-NMR spectrum for compound **4** recorded in $CDCl_3$

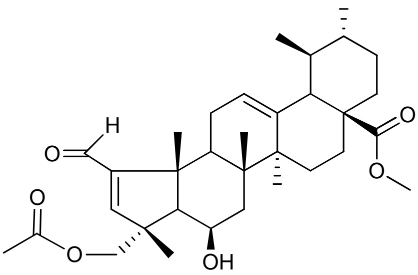


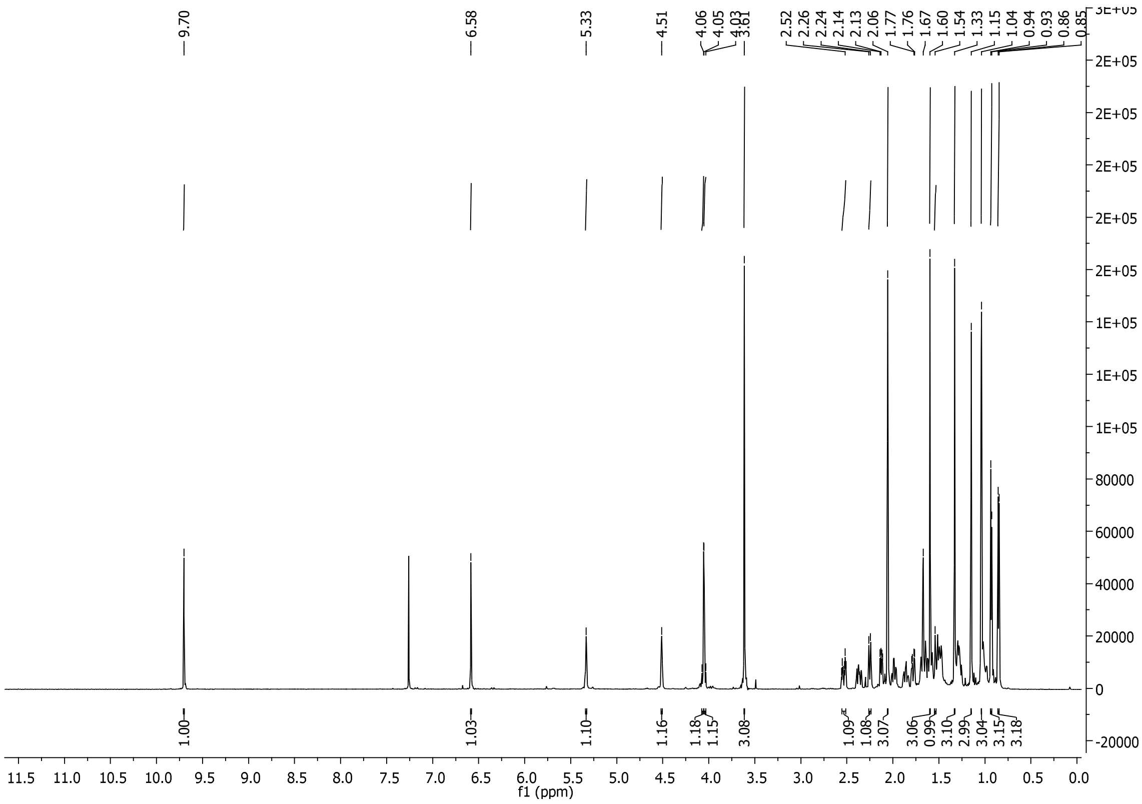

2.2 $^{13}$C-NMR spectrum for compound **4** recorded in $CDCl_3$

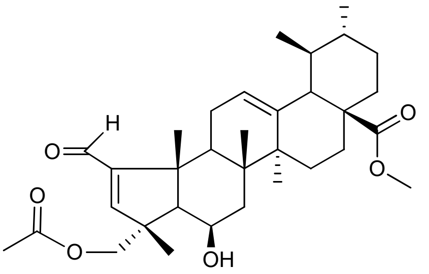

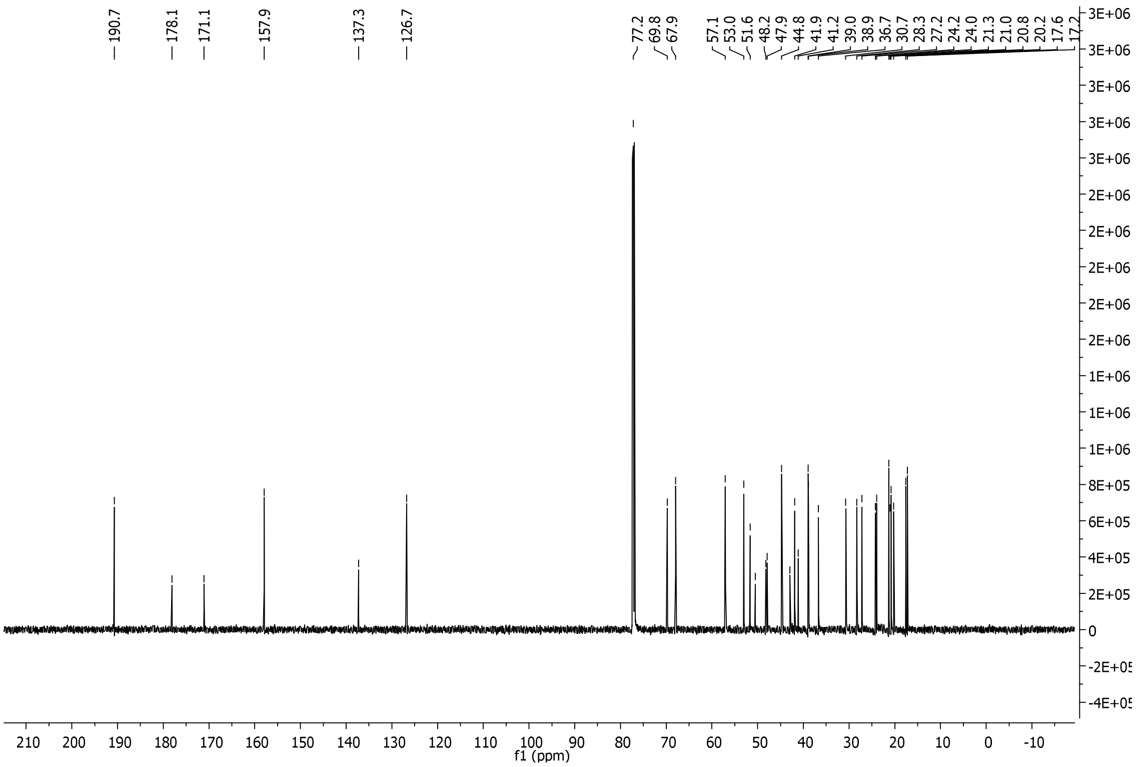

## 3. NMR data for compound 5

3.1 $^1$H-NMR spectrum for compound **5** recorded in $CDCl_3$

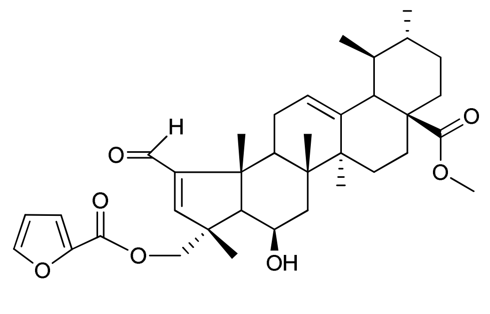

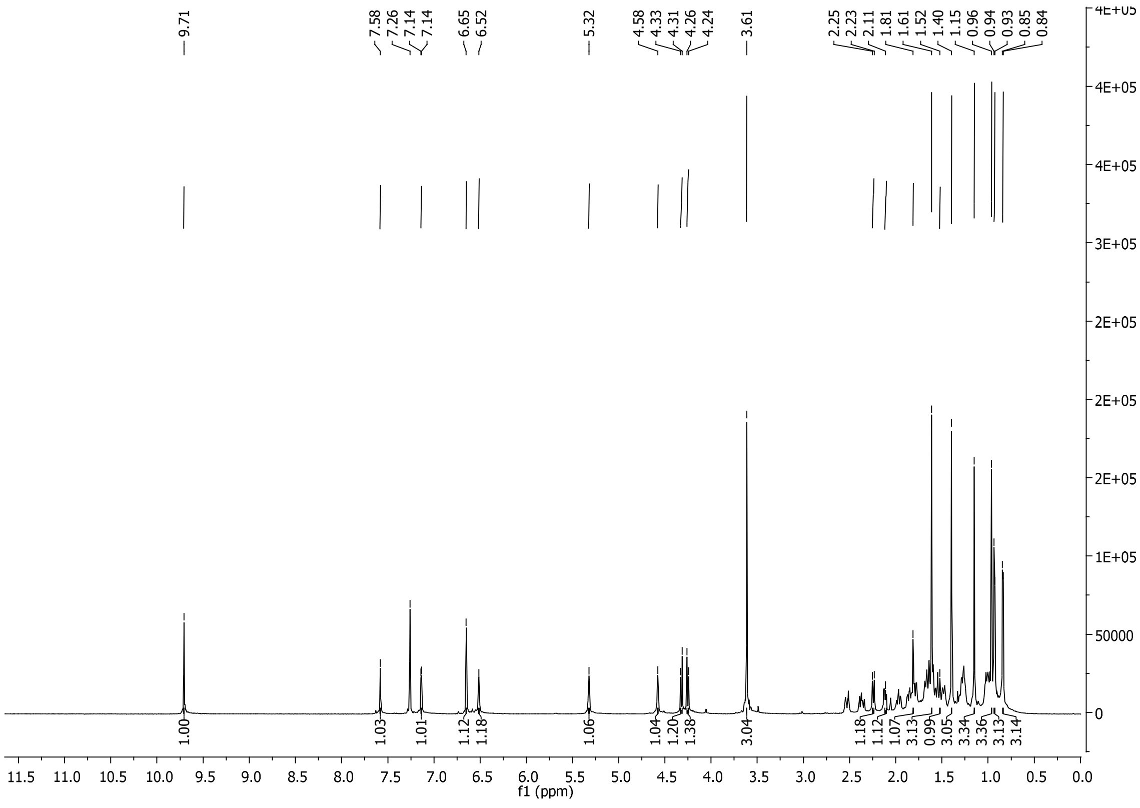

3.2 $^{13}C$-NMR spectrum for compound **5** recorded in $CDCl_3$

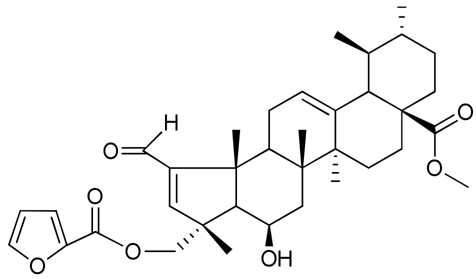


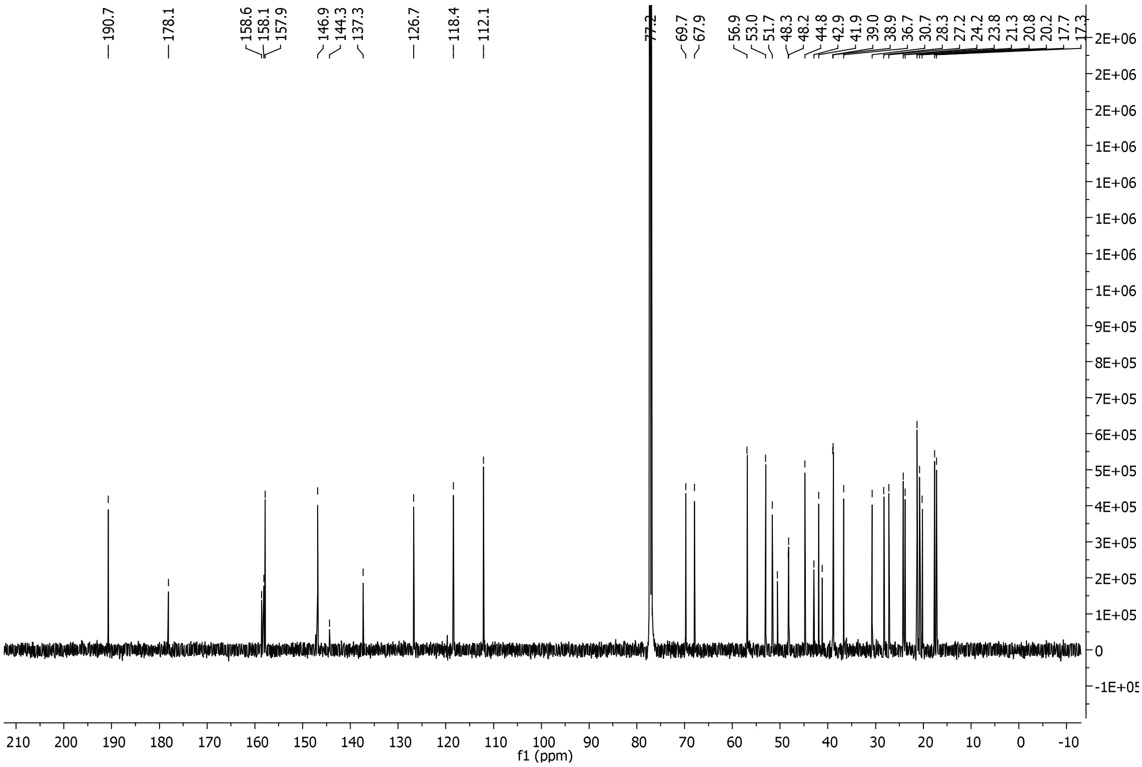

# 4. NMR data for compound 10

4.1 $^{1}$H-NMR spectrum for compound **10** recorded in $C_6D_6$

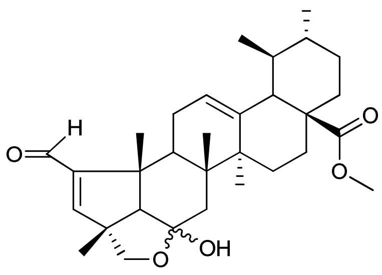

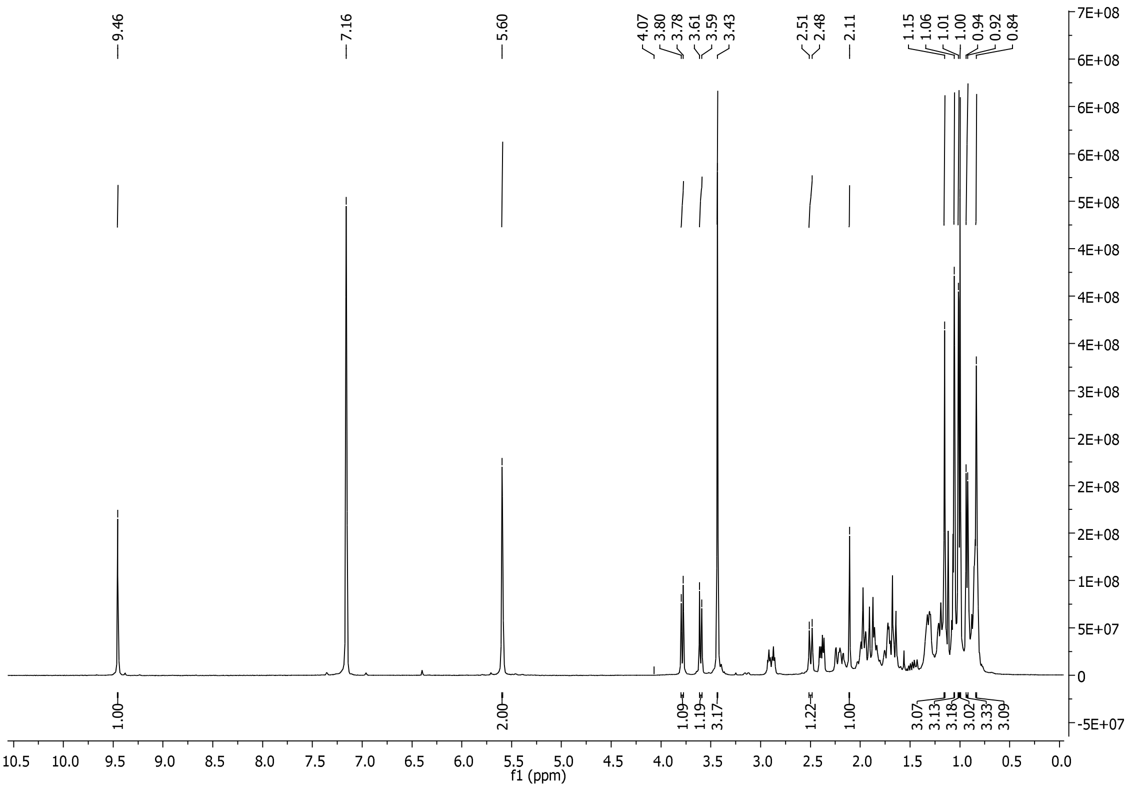

4.2 $^{13}$C-NMR spectrum for compound **10** recorded in $C_6D_6$

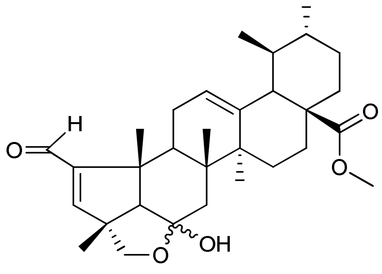


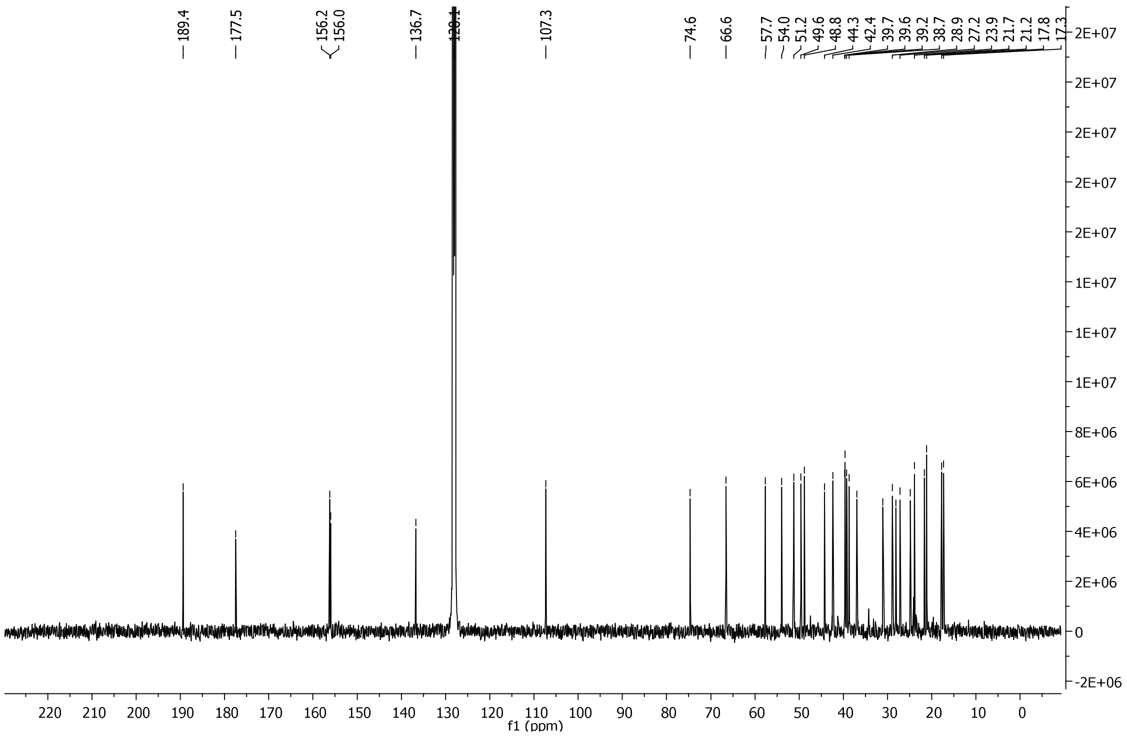

## 5. NMR data for compound 11

5.1 $^1$H-NMR spectrum for compound **11** recorded in $CDCl_3$

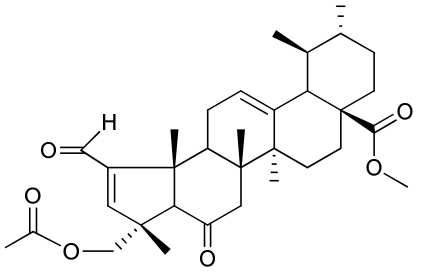

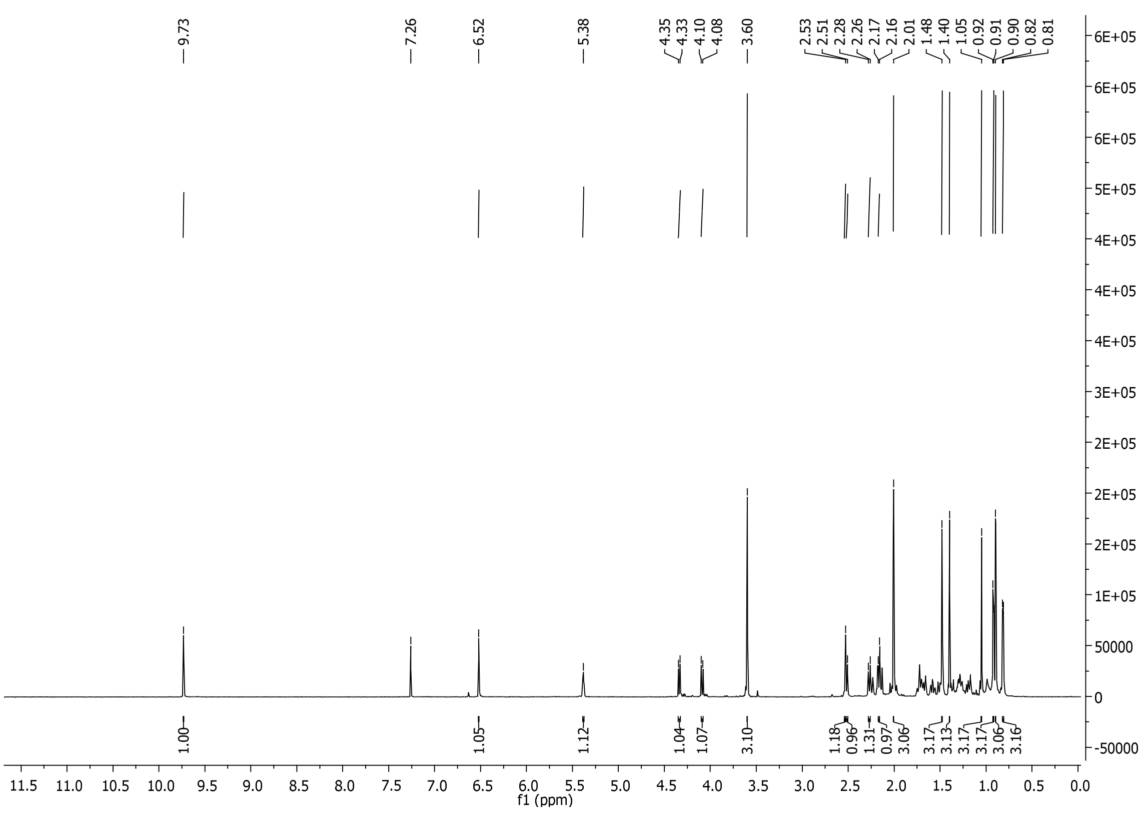

5.2 $^{13}$C-NMR spectrum for compound **11** recorded in $CDCl_3$

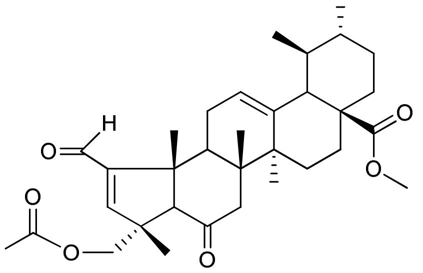


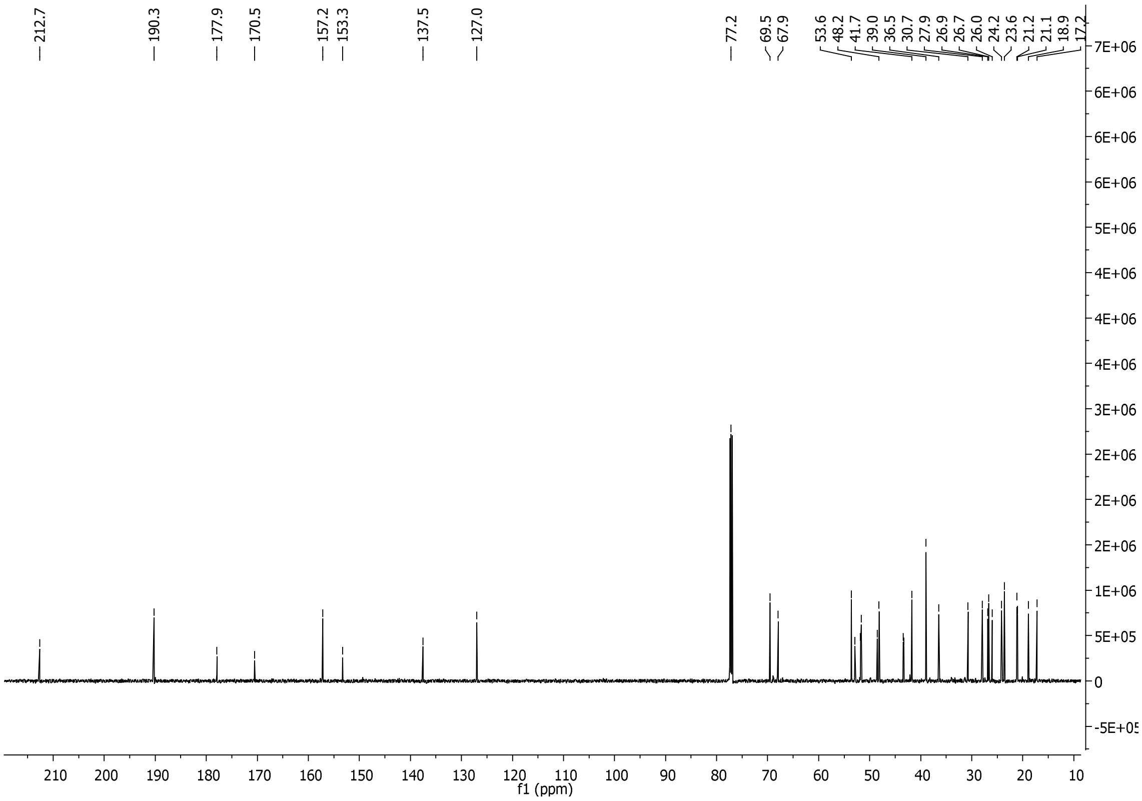

## 6. NMR data for compound 12

6.1 $^{1}$H-NMR spectrum for compound **12** recorded in $CDCl_3$

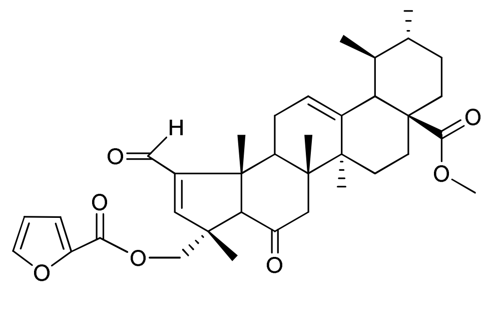


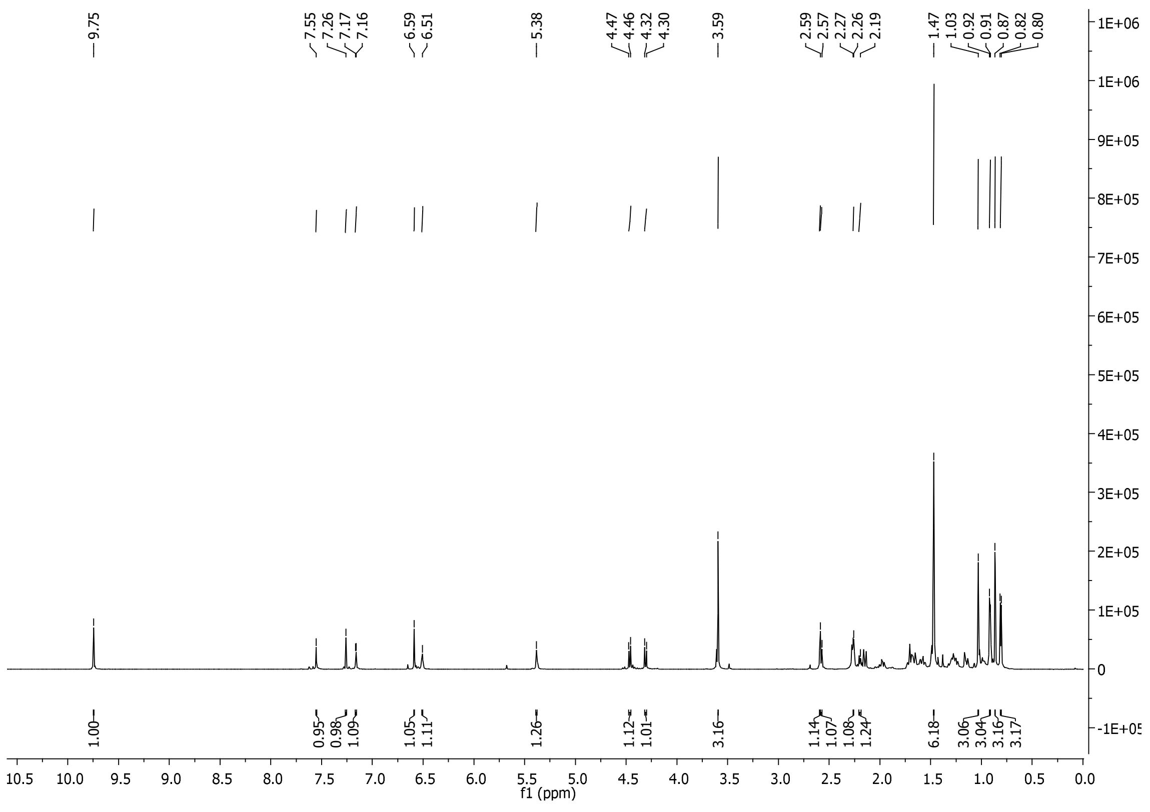

6.2 $^{13}$C-NMR spectrum for compound **12** recorded in $CDCl_3$

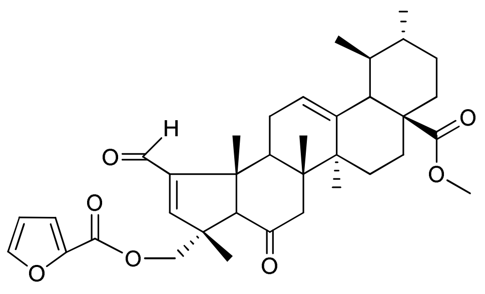

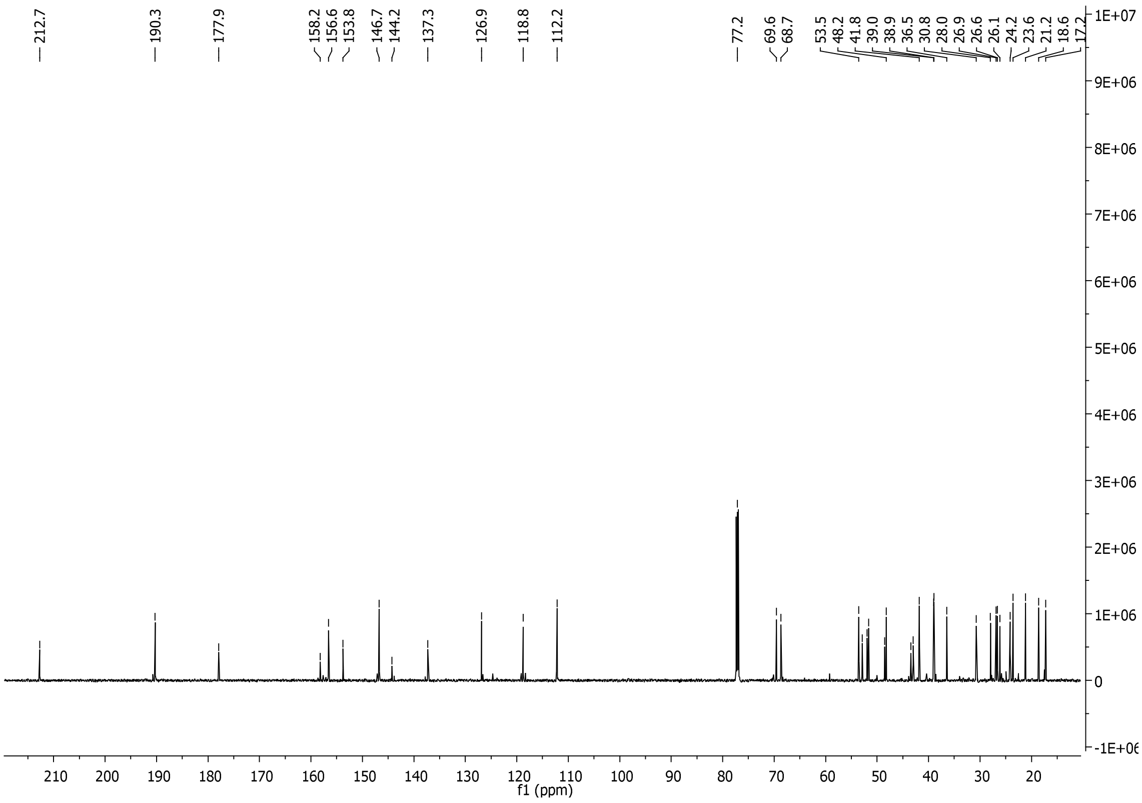

## 7. NMR data for compound 15

### 7.1 $^{1}$H-NMR spectrum for compound **15** recorded in $CDCl_3$

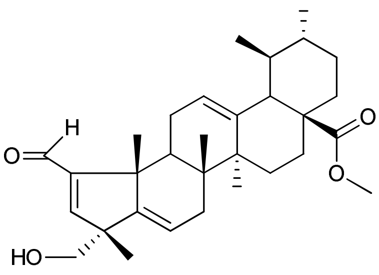


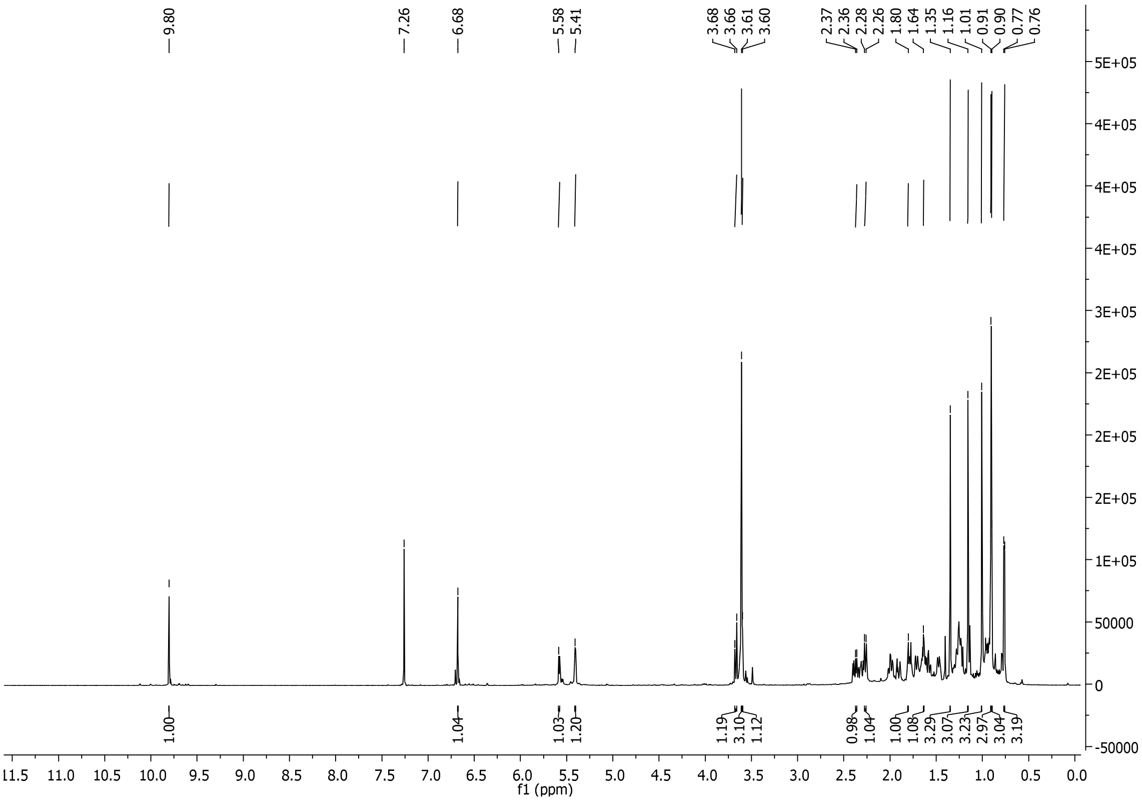

7.2 $^{13}$C-NMR spectrum for compound **15** recorded in $CDCl_3$

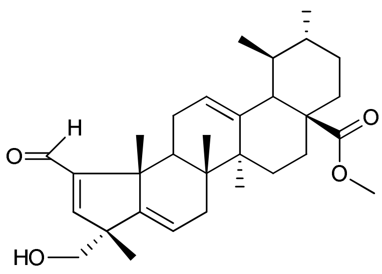


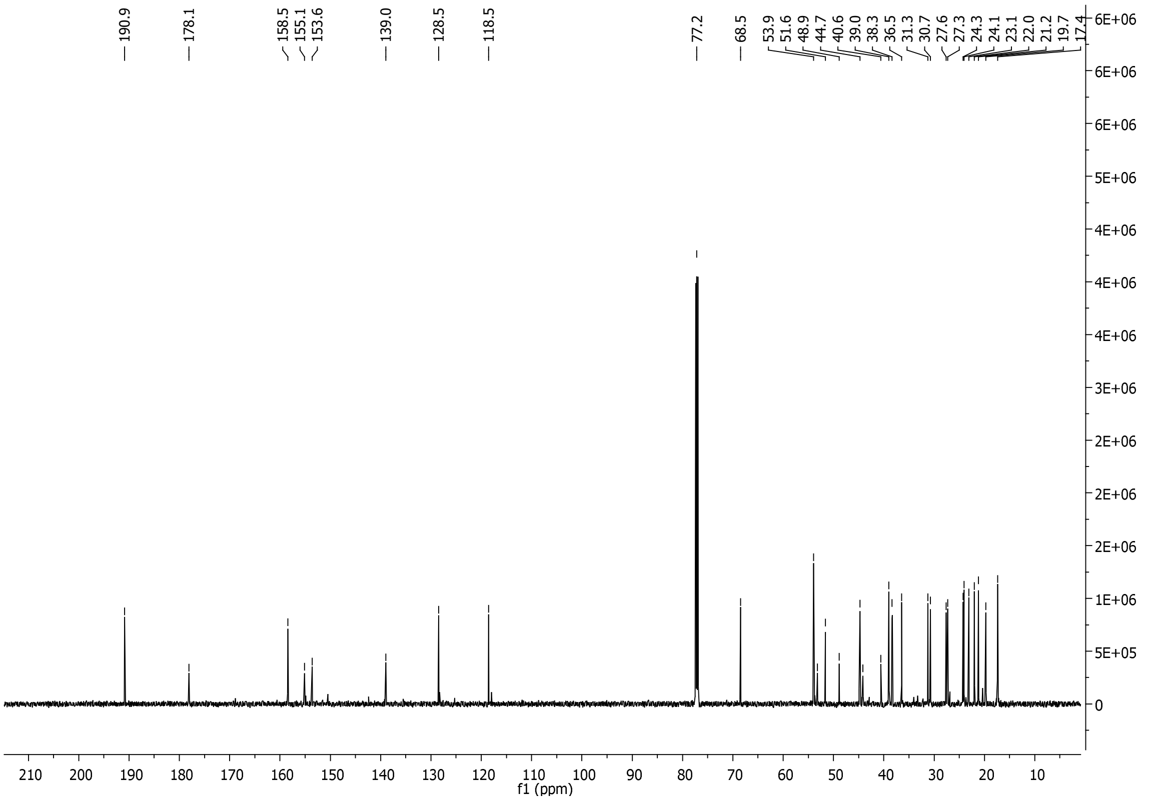

## 8. NMR data for compound 16

### 8.1 $^{1}$H-NMR spectrum for compound **16** recorded in $CDCl_3$

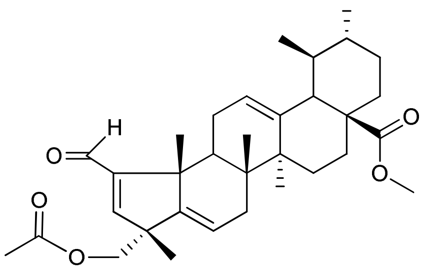

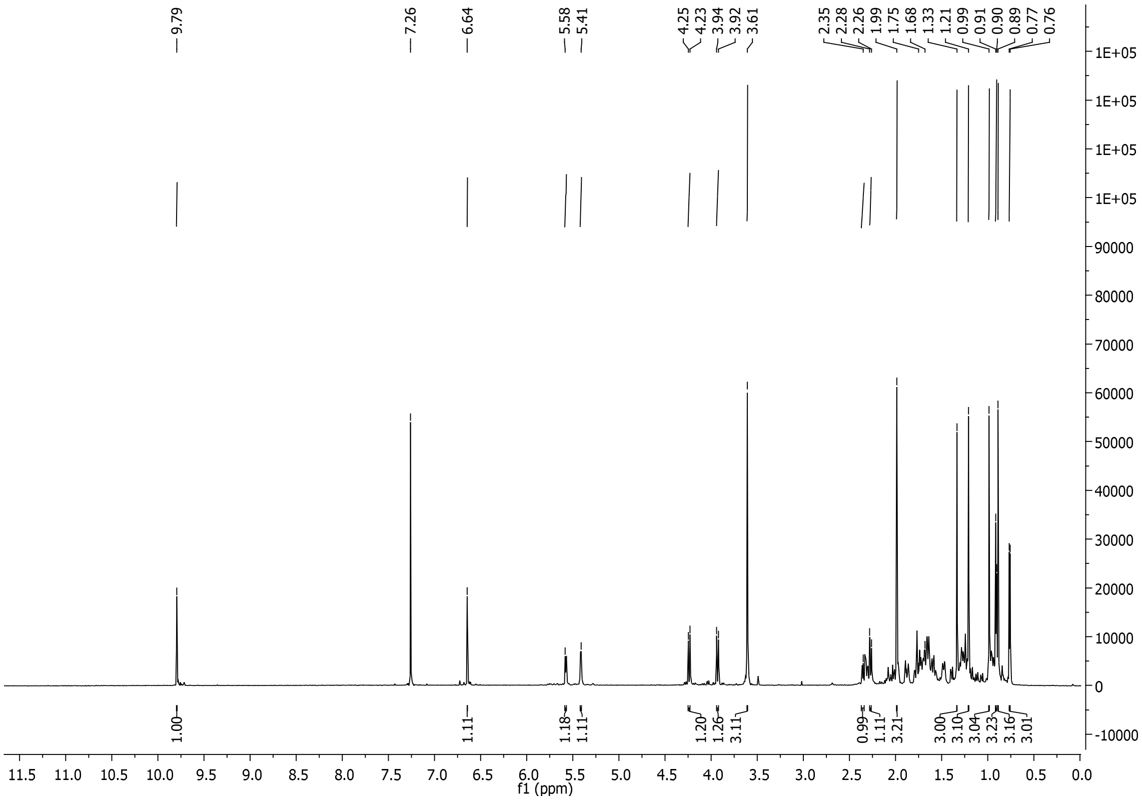

8.2 $^{13}$C-NMR spectrum for compound **16** recorded in $CDCl_3$

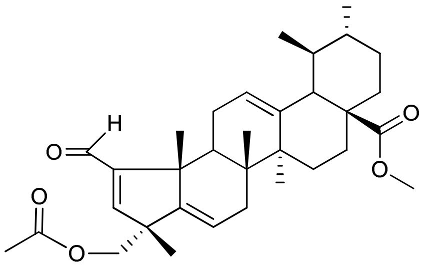

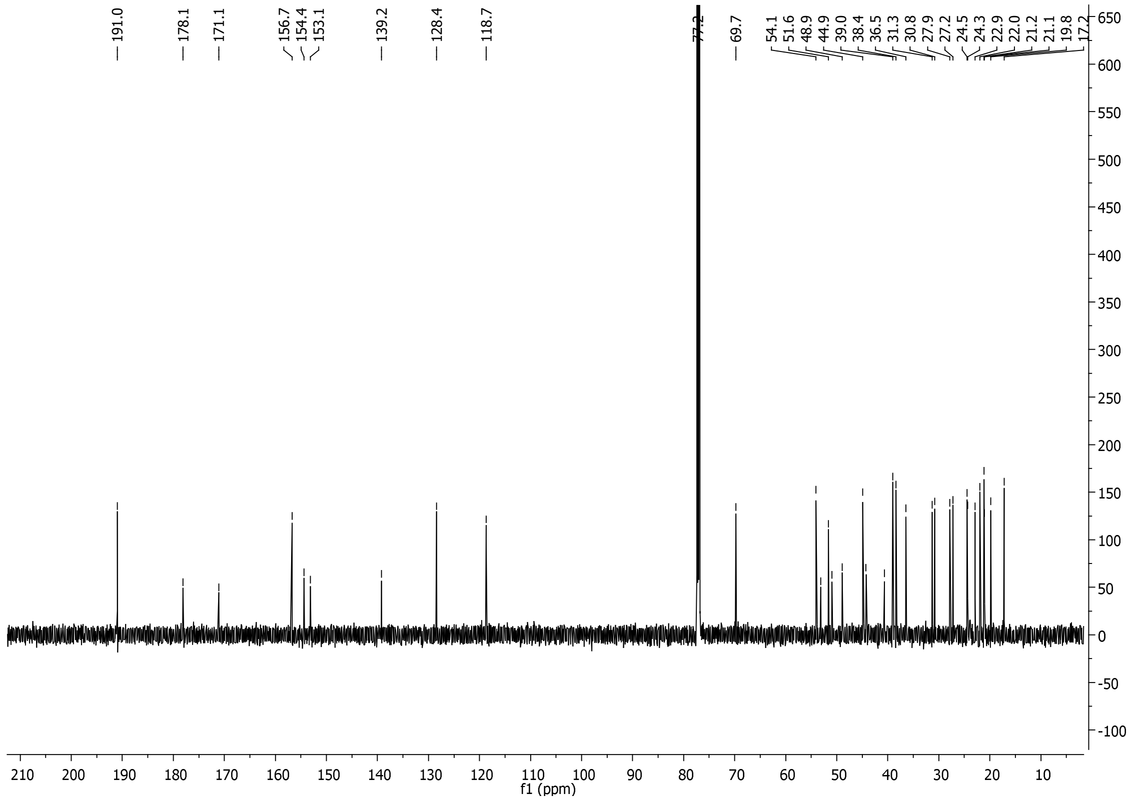

## 9. NMR data for compound 17

9.1 $^1$H-NMR spectrum for compound **17** recorded in $CDCl_3$

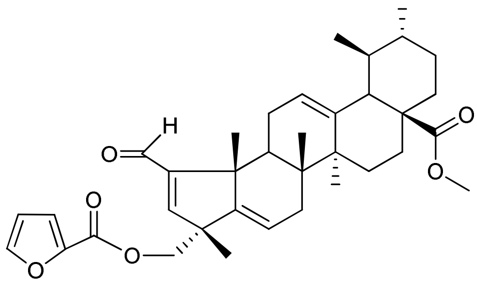


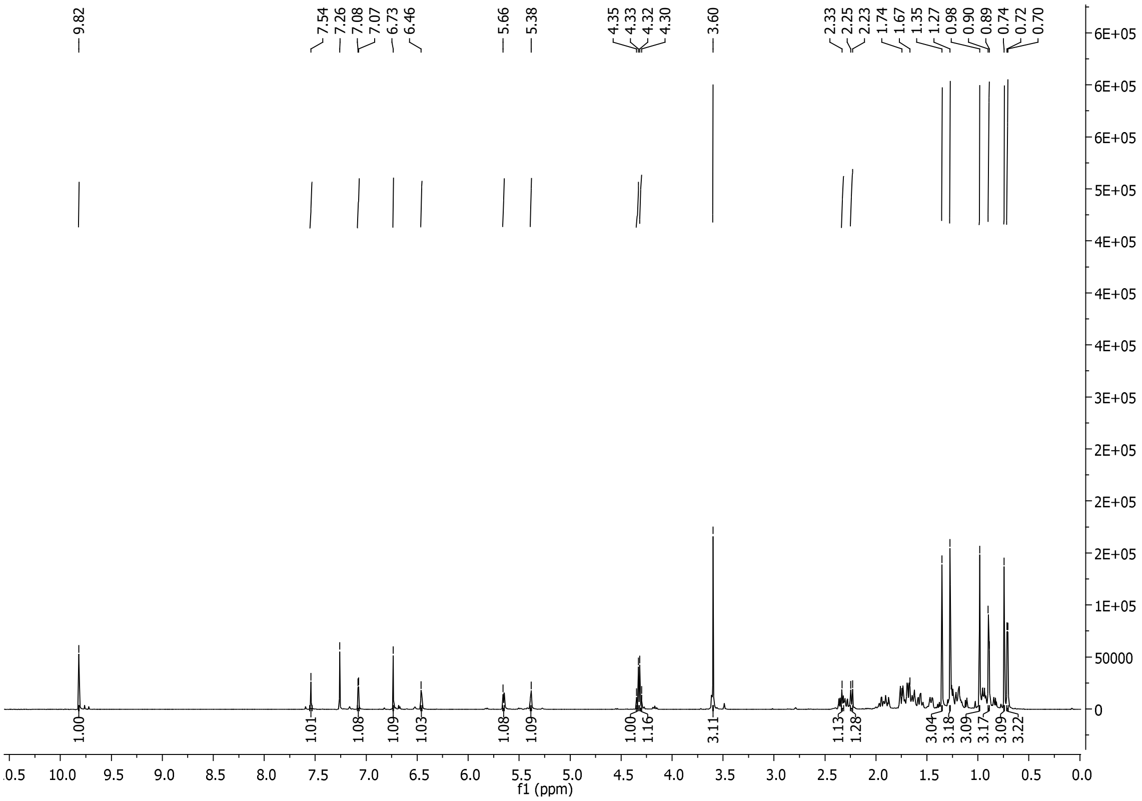

9.2 $^{13}$C-NMR spectrum for compound **17** recorded in $CDCl_3$

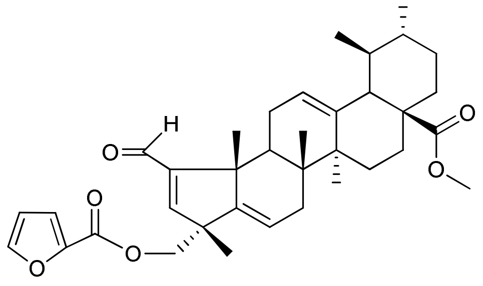

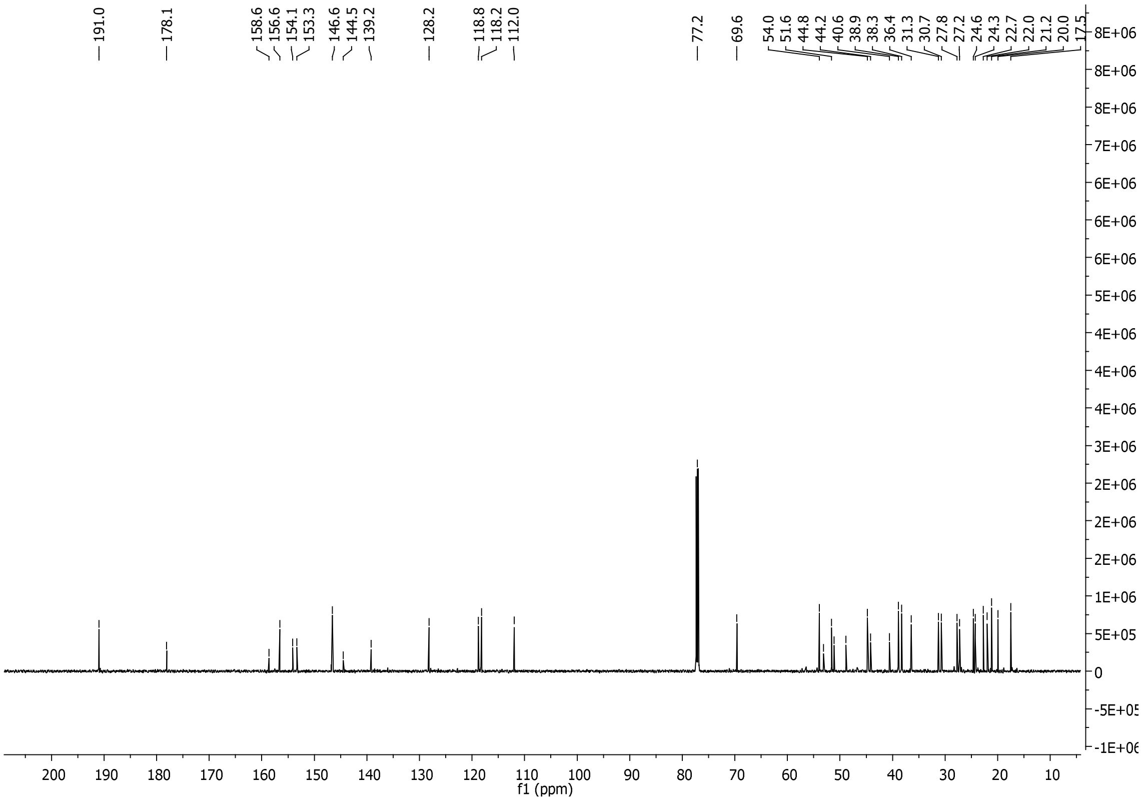

| Developmental Therapeutics Program One Dose Mean Graph | NSC: D-783350 / 1 | Conc: 1.00E-5 Molar | Test Date: Mar 09, 2015 |
|---|---|---|---|
| | Experiment ID: 1503OS44 | | Report Date: Mar 26, 2015 |

| Panel/Cell Line | Growth Percent |
|---|---|
| Leukemia | |
| CCRF-CEM | 94.76 |
| HL-60(TB) | 96.19 |
| K-562 | 97.40 |
| MOLT-4 | 100.02 |
| RPMI-8226 | 94.59 |
| SR | 97.85 |
| Non-Small Cell Lung Cancer | |
| A549/ATCC | 103.46 |
| EKVX | 114.16 |
| HOP-62 | 95.53 |
| HOP-92 | 74.37 |
| NCI-H226 | 96.68 |
| NCI-H23 | 103.66 |
| NCI-H322M | 96.98 |
| NCI-H460 | 105.29 |
| NCI-H522 | 93.55 |
| Colon Cancer | |
| COLO 205 | 104.33 |
| HCC-2998 | 108.29 |
| HCT-116 | 100.18 |
| HCT-15 | 109.67 |
| HT29 | 107.80 |
| KM12 | 104.35 |
| SW-620 | 102.72 |
| CNS Cancer | |
| SF-268 | 105.97 |
| SF-295 | 104.44 |
| SF-539 | 99.57 |
| SNB-19 | 94.65 |
| SNB-75 | 85.18 |
| U251 | 101.23 |
| Melanoma | |
| LOX IMVI | 95.86 |
| MALME-3M | 95.41 |
| M14 | 96.02 |
| MDA-MB-435 | 112.95 |
| SK-MEL-2 | 94.30 |
| SK-MEL-5 | 97.28 |
| Ovarian Cancer | |
| IGROV1 | 99.52 |
| OVCAR-3 | 121.84 |
| OVCAR-4 | 102.06 |
| OVCAR-5 | 97.87 |
| OVCAR-8 | 97.17 |
| NCI/ADR-RES | 106.94 |
| SK-OV-3 | 108.31 |
| Renal Cancer | |
| 786-0 | 109.35 |
| A498 | 82.21 |
| ACHN | 98.54 |
| CAKI-1 | 95.85 |
| RXF 393 | 102.55 |
| SN12C | 91.60 |
| TK-10 | 94.28 |
| UO-31 | 72.65 |
| Prostate Cancer | |
| PC-3 | 81.45 |
| DU-145 | 114.33 |
| Breast Cancer | |
| MCF7 | 96.33 |
| MDA-MB-231/ATCC | 95.36 |
| HS 578T | 92.97 |
| BT-549 | 117.94 |
| T-47D | 98.61 |
| MDA-MB-468 | 91.94 |
| Mean | 99.20 |
| Delta | 26.55 |
| Range | 49.19 |

**Mean Growth Percent - Growth Percent**

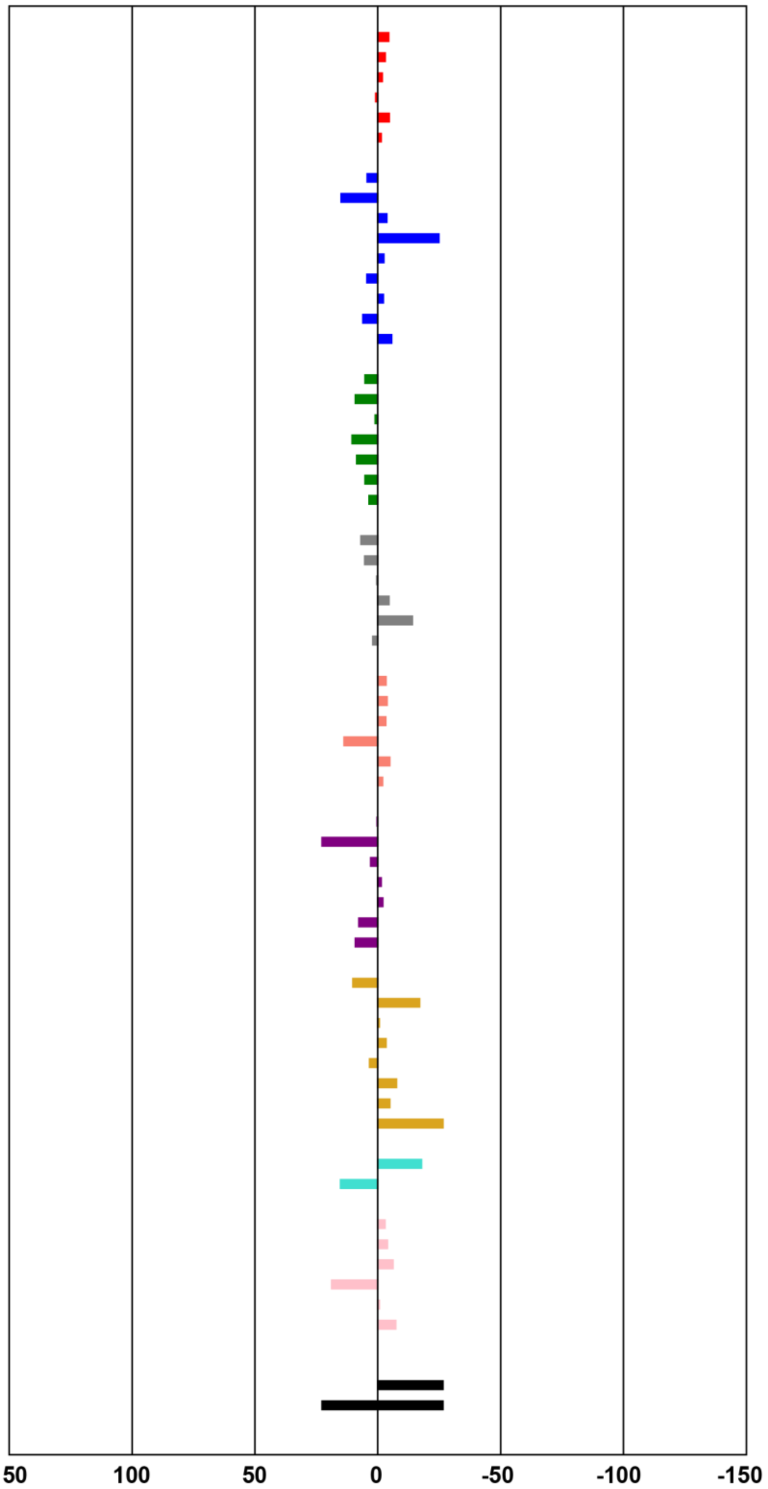


**Figure S1.** Mean growth percentages expressed by compound **1** (NSC 783350) at a single-dose concentration of 10 µM over the NCI-60 cancer cell lines. Zero on the X-axis represents the mean percentage of growth of the tested cell lines. The percentage of growth of each cell line relative to the mean is represented by a horizontal bar extending to the right side indicating more sensitivity or to the left side indicating less sensitivity.

| Developmental Therapeutics Program | NSC: D-787805 / 1 | Conc: 1.00E-5 Molar | Test Date: Nov 09, 2015 |
|---|---|---|---|
| One Dose Mean Graph | Experiment ID: 1511OS03 | | Report Date: Nov 30, 2015 |

| Panel/Cell Line | Growth Percent |
|---|---|
| Leukemia | |
| CCRF-CEM | -43.65 |
| HL-60(TB) | -27.40 |
| K-562 | -31.74 |
| MOLT-4 | -51.87 |
| RPMI-8226 | -20.73 |
| SR | -36.52 |
| Non-Small Cell Lung Cancer | |
| A549/ATCC | -62.29 |
| EKVX | -33.13 |
| HOP-62 | -59.46 |
| HOP-92 | -56.02 |
| NCI-H226 | -42.81 |
| NCI-H23 | -50.00 |
| NCI-H322M | -28.99 |
| NCI-H460 | -72.50 |
| NCI-H522 | -78.88 |
| Colon Cancer | |
| COLO 205 | -87.47 |
| HCC-2998 | -89.21 |
| HCT-116 | -82.26 |
| HCT-15 | -51.43 |
| HT29 | -90.47 |
| KM12 | -74.24 |
| SW-620 | -54.10 |
| CNS Cancer | |
| SF-268 | -30.98 |
| SF-295 | -78.53 |
| SF-539 | -58.45 |
| SNB-19 | -23.37 |
| SNB-75 | -37.72 |
| U251 | -94.64 |
| Melanoma | |
| LOX IMVI | -93.02 |
| MALME-3M | -55.94 |
| M14 | -71.30 |
| MDA-MB-435 | -66.90 |
| SK-MEL-2 | -88.21 |
| SK-MEL-28 | -48.37 |
| SK-MEL-5 | -95.78 |
| UACC-257 | -85.07 |
| UACC-62 | -64.01 |
| Ovarian Cancer | |
| IGROV1 | -19.84 |
| OVCAR-3 | -64.91 |
| OVCAR-4 | -11.01 |
| OVCAR-5 | -21.58 |
| OVCAR-8 | -27.31 |
| NCI/ADR-RES | -22.78 |
| SK-OV-3 | -33.14 |
| Renal Cancer | |
| 786-0 | -65.12 |
| A498 | -80.21 |
| ACHN | -48.31 |
| CAKI-1 | 0.47 |
| RXF 393 | -81.57 |
| TK-10 | -56.90 |
| UO-31 | -52.23 |
| Prostate Cancer | |
| PC-3 | -34.51 |
| DU-145 | -82.50 |
| Breast Cancer | |
| MCF7 | -24.27 |
| MDA-MB-231/ATCC | -66.29 |
| HS 578T | -13.03 |
| BT-549 | -66.09 |
| T-47D | -47.11 |
| MDA-MB-468 | -53.01 |
| Mean | -54.05 |
| Delta | 41.73 |
| Range | 96.25 |

Mean Growth Percent - Growth Percent

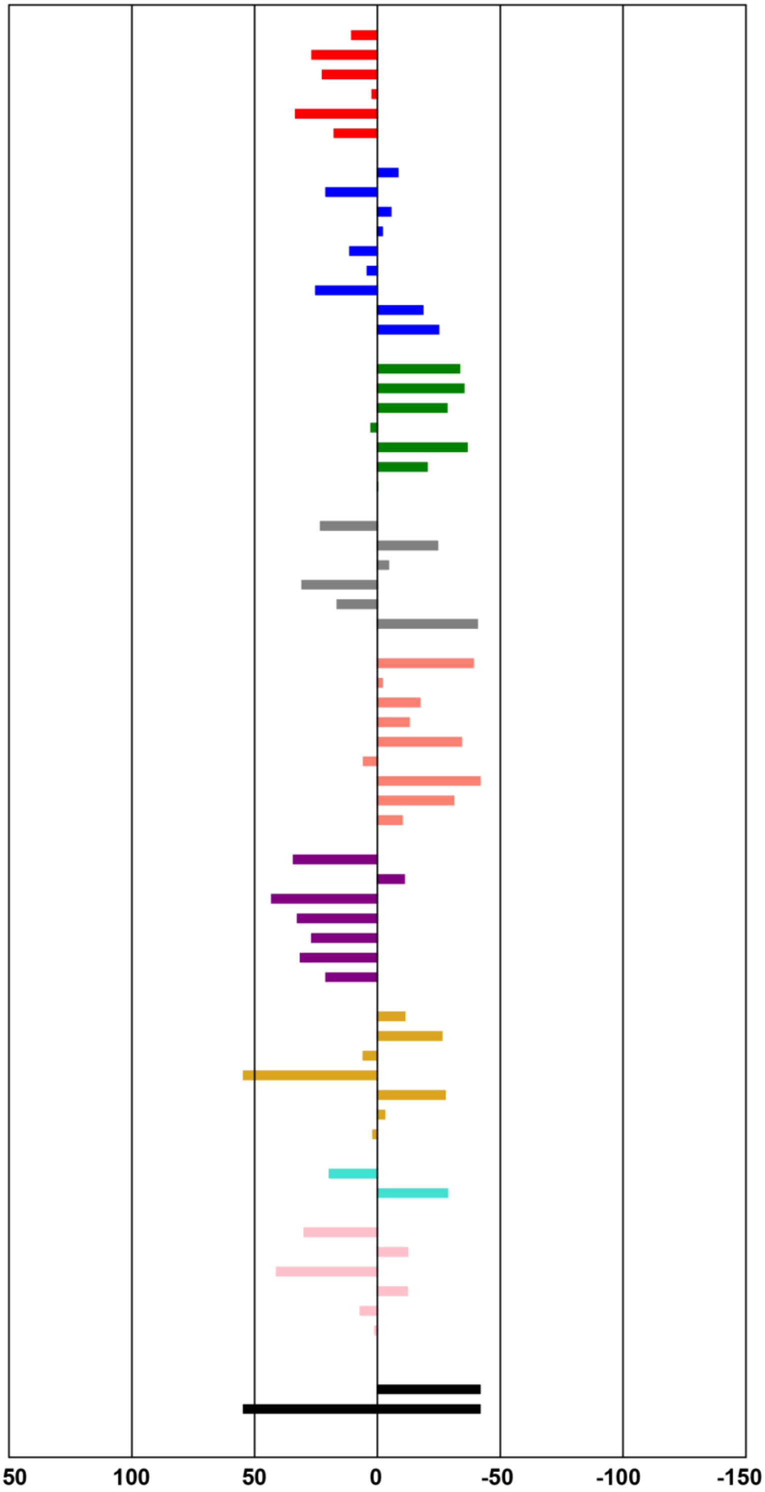


**Figure S2.** Mean growth percentages expressed by compound **2** (NSC 787805) at a single-dose concentration of 10 µM over the NCI-60 cancer cell lines. Zero on the X-axis represents the mean percentage of growth of the tested cell lines. The percentage of growth of each cell line relative to the mean is represented by a horizontal bar extending to the right side indicating more sensitivity or to the left side indicating less sensitivity.

| Developmental Therapeutics Program One Dose Mean Graph | NSC: D-784255 / 1 | Conc: 1.00E-5 Molar | Test Date: Apr 27, 2015 |
|---|---|---|---|
| | Experiment ID: 1504OS80 | | Report Date: May 21, 2015 |

| Panel/Cell Line | Growth Percent |
|---|---|
| Leukemia | |
| CCRF-CEM | 1.38 |
| HL-60(TB) | -14.37 |
| K-562 | 6.68 |
| RPMI-8226 | -10.09 |
| SR | -20.42 |
| Non-Small Cell Lung Cancer | |
| A549/ATCC | -1.72 |
| EKVX | 7.75 |
| HOP-62 | 11.93 |
| HOP-92 | -22.82 |
| NCI-H226 | 3.36 |
| NCI-H23 | -17.06 |
| NCI-H322M | 32.93 |
| NCI-H460 | -34.98 |
| NCI-H522 | -37.54 |
| Colon Cancer | |
| COLO 205 | -27.97 |
| HCC-2998 | -79.17 |
| HCT-116 | -81.36 |
| HCT-15 | -68.09 |
| HT29 | -37.66 |
| KM12 | 1.71 |
| SW-620 | -1.77 |
| CNS Cancer | |
| SF-268 | -0.09 |
| SF-295 | -15.57 |
| SF-539 | -44.03 |
| SNB-19 | 24.39 |
| SNB-75 | 3.20 |
| U251 | -19.01 |
| Melanoma | |
| LOX IMVI | -97.95 |
| MALME-3M | -70.73 |
| M14 | -51.54 |
| MDA-MB-435 | -15.99 |
| SK-MEL-2 | -31.74 |
| SK-MEL-28 | -34.47 |
| SK-MEL-5 | -85.74 |
| UACC-257 | -48.44 |
| UACC-62 | -34.50 |
| Ovarian Cancer | |
| IGROV1 | 3.94 |
| OVCAR-3 | -91.93 |
| OVCAR-4 | -5.06 |
| OVCAR-5 | -19.68 |
| OVCAR-8 | -2.42 |
| NCI/ADR-RES | -10.17 |
| SK-OV-3 | 3.19 |
| Renal Cancer | |
| 786-0 | -30.41 |
| A498 | -5.03 |
| ACHN | -81.64 |
| CAKI-1 | -28.68 |
| RXF 393 | -82.02 |
| SN12C | 1.62 |
| TK-10 | -18.81 |
| UO-31 | -31.55 |
| Prostate Cancer | |
| PC-3 | -0.11 |
| DU-145 | -30.04 |
| Breast Cancer | |
| MCF7 | 3.73 |
| MDA-MB-231/ATCC | -47.56 |
| HS 578T | -15.62 |
| BT-549 | -15.60 |
| T-47D | -19.80 |
| MDA-MB-468 | -12.01 |
| Mean | -24.53 |
| Delta | 73.42 |
| Range | 130.88 |

Mean Growth Percent - Growth Percent

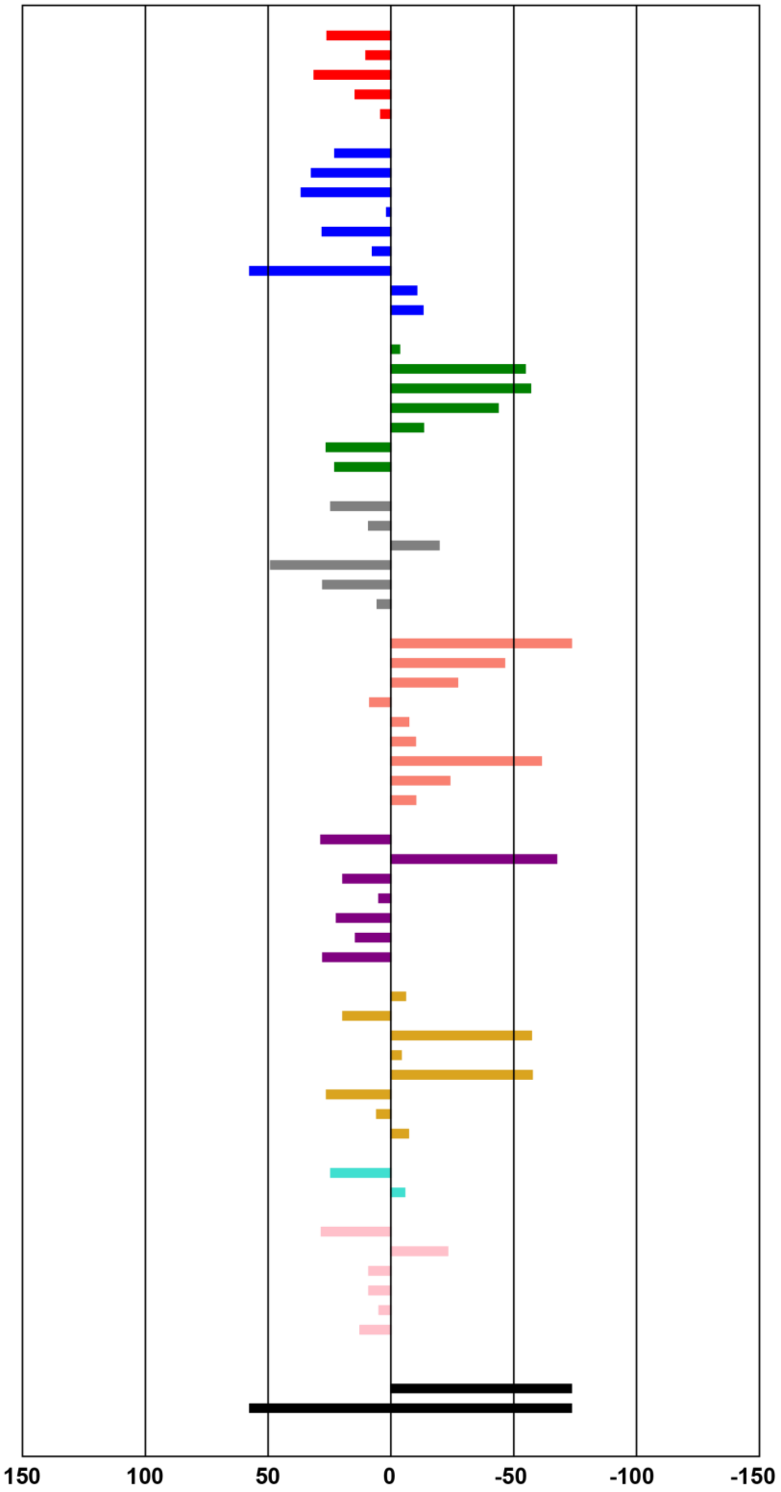


**Figure S3.** Mean growth percentages expressed by compound **3** (NSC 784255) at a single-dose concentration of 10 µM over the NCI-60 cancer cell lines. Zero on the X-axis represents the mean percentage of growth of the tested cell lines. The percentage of growth of each cell line relative to the mean is represented by a horizontal bar extending to the right side indicating more sensitivity or to the left side indicating less sensitivity.

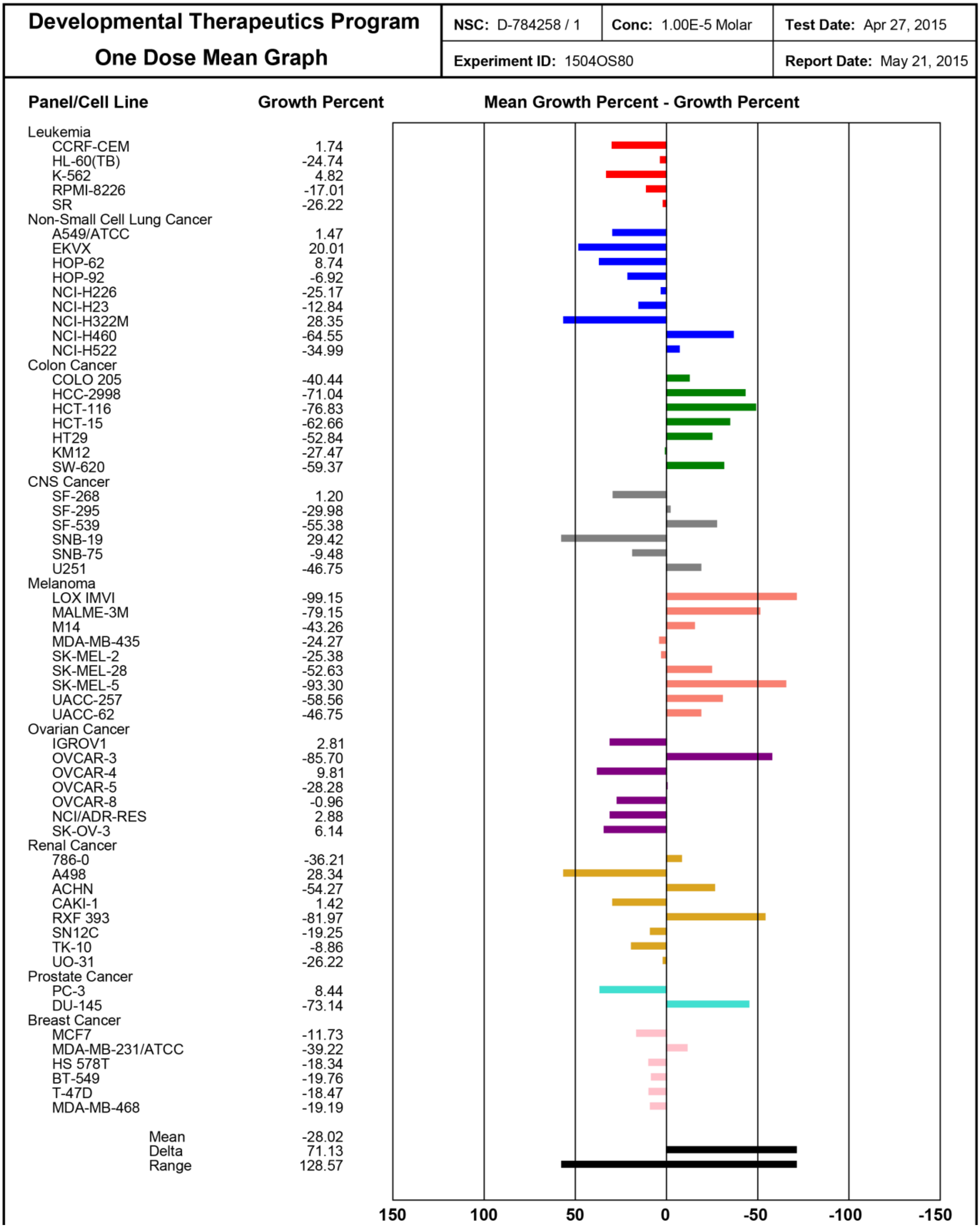


**Figure S4.** Mean growth percentages expressed by compound **4** (NSC 784258) at a single-dose concentration of 10 µM over the NCI-60 cancer cell lines. Zero on the X-axis represents the mean percentage of growth of the tested cell lines. The percentage of growth of each cell line relative to the mean is represented by a horizontal bar extending to the right side indicating more sensitivity or to the left side indicating less sensitivity.

| Developmental Therapeutics Program<br>One Dose Mean Graph | NSC: D-783356 / 1 | Conc: 1.00E-5 Molar | Test Date: Mar 09, 2015 |
|---|---|---|---|
| | Experiment ID: 1503OS44 | | Report Date: Mar 26, 2015 |

| Panel/Cell Line | Growth Percent |
|---|---|
| Leukemia | |
| CCRF-CEM | -24.10 |
| HL-60(TB) | -21.93 |
| K-562 | 2.92 |
| MOLT-4 | -0.74 |
| RPMI-8226 | -26.84 |
| SR | 0.40 |
| Non-Small Cell Lung Cancer | |
| A549/ATCC | 7.92 |
| EKVX | -18.11 |
| HOP-62 | -7.36 |
| HOP-92 | -54.66 |
| NCI-H226 | -41.53 |
| NCI-H23 | -13.52 |
| NCI-H322M | 33.05 |
| NCI-H460 | -59.64 |
| NCI-H522 | -47.89 |
| Colon Cancer | |
| COLO 205 | -78.57 |
| HCC-2998 | -84.84 |
| HCT-116 | -75.78 |
| HCT-15 | -78.29 |
| HT29 | -64.36 |
| KM12 | -56.85 |
| SW-620 | -55.66 |
| CNS Cancer | |
| SF-268 | 2.92 |
| SF-295 | -76.20 |
| SF-539 | -62.58 |
| SNB-19 | 7.80 |
| U251 | -76.77 |
| Melanoma | |
| LOX IMVI | -91.44 |
| MALME-3M | -63.62 |
| M14 | -23.25 |
| MDA-MB-435 | -57.45 |
| SK-MEL-2 | -44.45 |
| SK-MEL-5 | -94.93 |
| Ovarian Cancer | |
| IGROV1 | 5.24 |
| OVCAR-3 | -75.53 |
| OVCAR-4 | -0.26 |
| OVCAR-5 | -20.89 |
| OVCAR-8 | -3.22 |
| NCI/ADR-RES | 0.30 |
| SK-OV-3 | 18.74 |
| Renal Cancer | |
| 786-0 | -22.95 |
| A498 | -2.10 |
| ACHN | -47.70 |
| CAKI-1 | -6.76 |
| RXF 393 | -62.99 |
| SN12C | -19.73 |
| TK-10 | 11.49 |
| UO-31 | -76.26 |
| Prostate Cancer | |
| PC-3 | -8.00 |
| DU-145 | -74.27 |
| Breast Cancer | |
| MCF7 | -19.17 |
| MDA-MB-231/ATCC | -47.39 |
| HS 578T | -9.48 |
| BT-549 | -3.09 |
| T-47D | 13.39 |
| MDA-MB-468 | -12.15 |
| Mean | -32.31 |
| Delta | 62.62 |
| Range | 127.98 |

Mean Growth Percent - Growth Percent

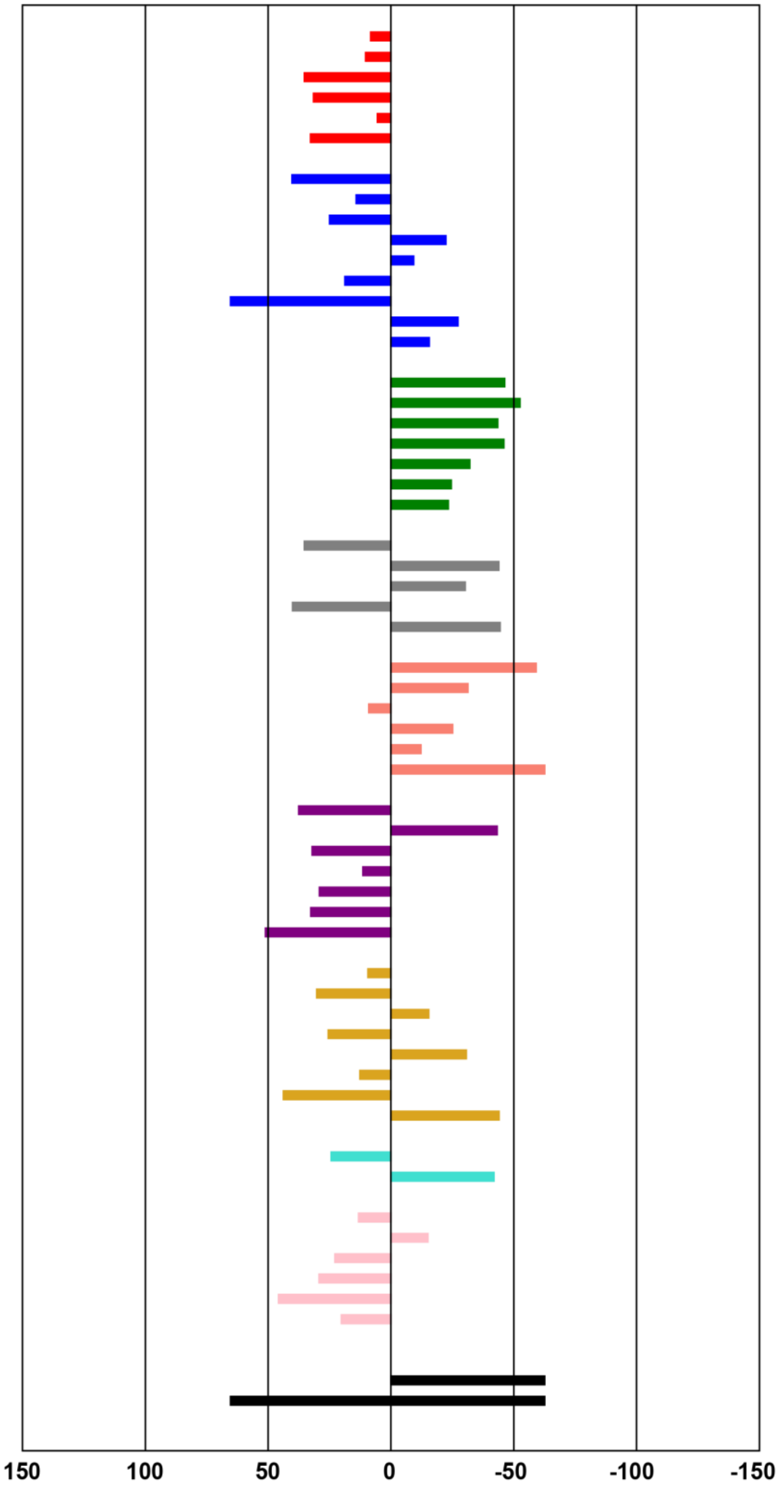


**Figure S5.** Mean growth percentages expressed by compound **5** (NSC 783356) at a single-dose concentration of 10 µM over the NCI-60 cancer cell lines. Zero on the X-axis represents the mean percentage of growth of the tested cell lines. The percentage of growth of each cell line relative to the mean is represented by a horizontal bar extending to the right side indicating more sensitivity or to the left side indicating less sensitivity.

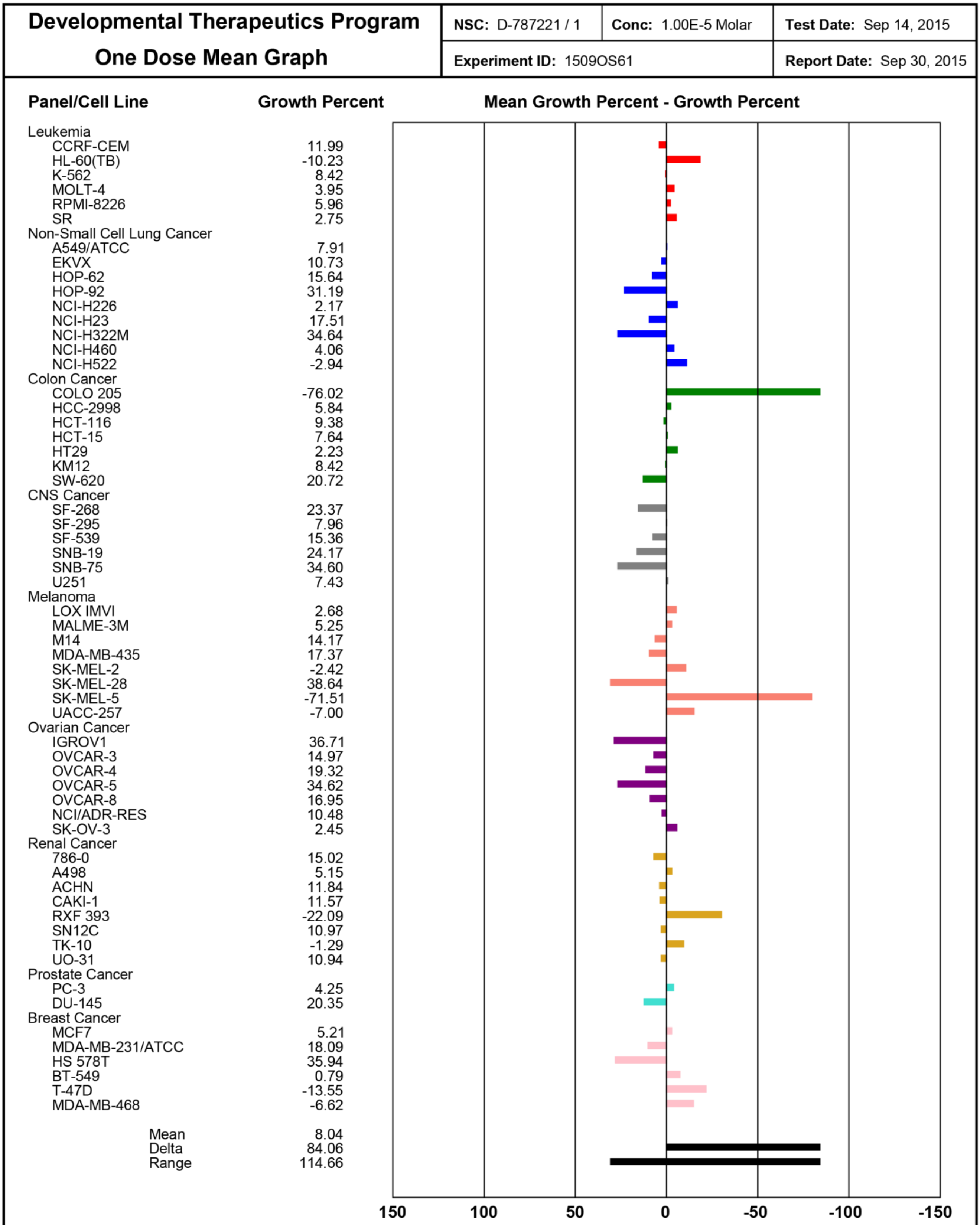


**Figure S6.** Mean growth percentages expressed by compound **6** (NSC 787221) at a single-dose concentration of 10 µM over the NCI-60 cancer cell lines. Zero on the X-axis represents the mean percentage of growth of the tested cell lines. The percentage of growth of each cell line relative to the mean is represented by a horizontal bar extending to the right side indicating more sensitivity or to the left side indicating less sensitivity.

| Developmental Therapeutics Program | NSC: D-783355 / 1 | Conc: 1.00E-5 Molar | Test Date: Mar 09, 2015 |
|---|---|---|---|
| One Dose Mean Graph | Experiment ID: 1503OS44 | | Report Date: Mar 26, 2015 |

| Panel/Cell Line | Growth Percent |
|---|---|
| Leukemia | |
| CCRF-CEM | -7.54 |
| HL-60(TB) | -13.30 |
| K-562 | 3.89 |
| MOLT-4 | -9.93 |
| RPMI-8226 | -19.30 |
| SR | -14.35 |
| Non-Small Cell Lung Cancer | |
| A549/ATCC | 7.39 |
| EKVX | 20.96 |
| HOP-62 | 13.04 |
| HOP-92 | -28.65 |
| NCI-H226 | -21.54 |
| NCI-H23 | 5.09 |
| NCI-H322M | 27.56 |
| NCI-H460 | -13.00 |
| NCI-H522 | -41.89 |
| Colon Cancer | |
| COLO 205 | -94.38 |
| HCC-2998 | 6.06 |
| HCT-116 | -44.39 |
| HCT-15 | 5.82 |
| HT29 | -59.27 |
| KM12 | 6.15 |
| SW-620 | 16.95 |
| CNS Cancer | |
| SF-268 | 34.86 |
| SF-295 | 1.62 |
| SF-539 | 7.45 |
| SNB-19 | 27.11 |
| SNB-75 | 13.27 |
| U251 | 0.22 |
| Melanoma | |
| LOX IMVI | -34.20 |
| MALME-3M | -8.79 |
| M14 | 6.13 |
| MDA-MB-435 | 2.15 |
| SK-MEL-2 | -22.39 |
| SK-MEL-5 | -91.27 |
| Ovarian Cancer | |
| IGROV1 | 14.75 |
| OVCAR-3 | -12.01 |
| OVCAR-4 | 18.55 |
| OVCAR-5 | -10.52 |
| OVCAR-8 | -4.45 |
| NCI/ADR-RES | 10.88 |
| SK-OV-3 | -0.33 |
| Renal Cancer | |
| 786-0 | 2.91 |
| A498 | -0.98 |
| ACHN | 7.86 |
| CAKI-1 | 7.25 |
| RXF 393 | -7.01 |
| SN12C | 11.39 |
| TK-10 | 9.25 |
| UO-31 | 7.68 |
| Prostate Cancer | |
| PC-3 | 5.85 |
| DU-145 | 14.53 |
| Breast Cancer | |
| MCF7 | 6.16 |
| MDA-MB-231/ATCC | -14.86 |
| HS 578T | 16.82 |
| BT-549 | -10.11 |
| T-47D | -4.48 |
| MDA-MB-468 | -0.84 |
| Mean | -4.39 |
| Delta | 89.99 |
| Range | 129.24 |

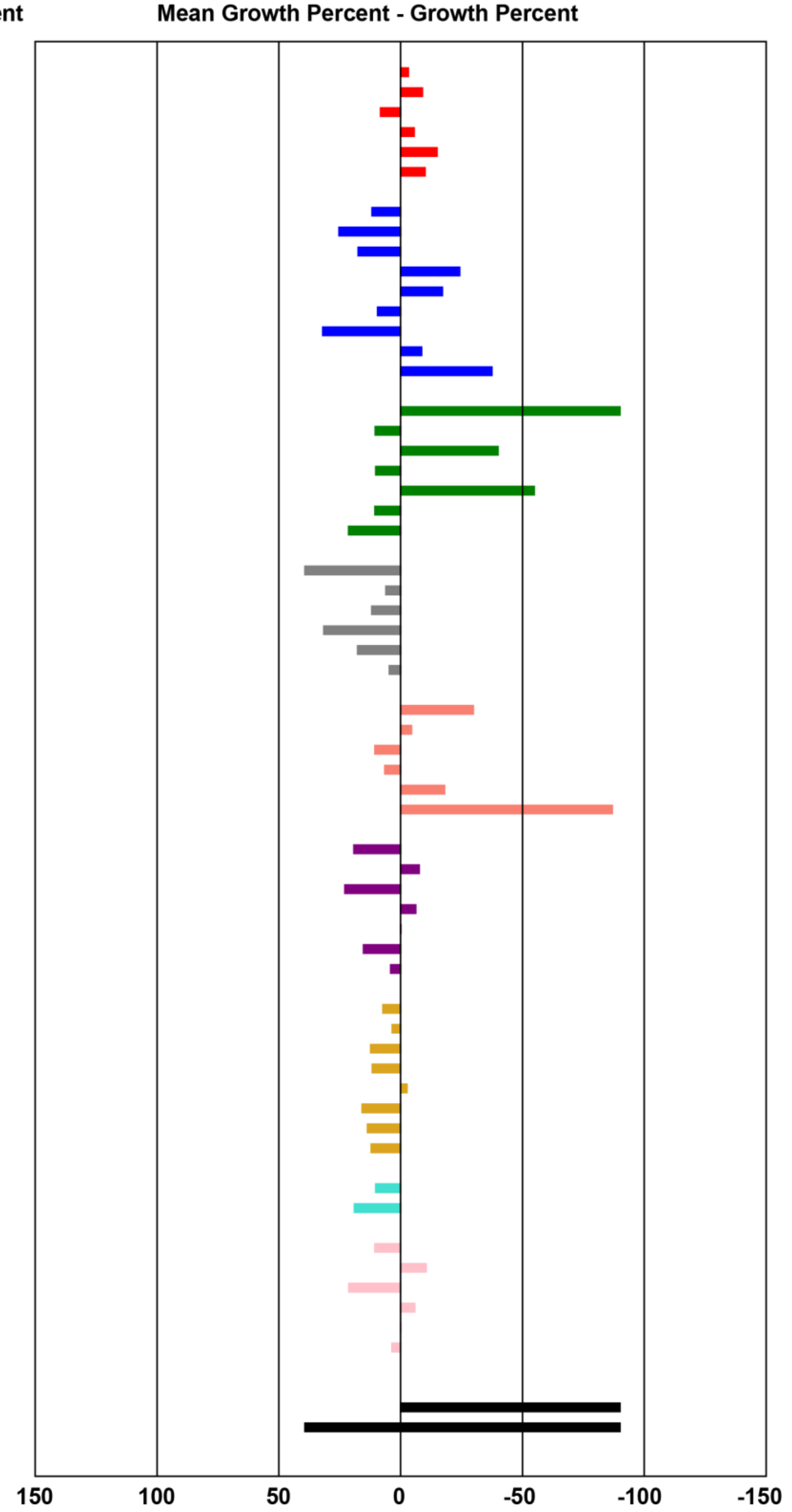


**Figure S7.** Mean growth percentages expressed by compound **9** (NSC 783355) at a single-dose concentration of 10 µM over the NCI-60 cancer cell lines. Zero on the X-axis represents the mean percentage of growth of the tested cell lines. The percentage of growth of each cell line relative to the mean is represented by a horizontal bar extending to the right side indicating more sensitivity or to the left side indicating less sensitivity.

**Developmental Therapeutics Program**

**One Dose Mean Graph**

**NSC:** D-785397 / 1 | **Conc:** 1.00E-5 Molar | **Test Date:** Jul 13, 2015

**Experiment ID:** 1507OS19 | **Report Date:** Jul 31, 2015

| Panel/Cell Line | Growth Percent |
|---|---|
| Leukemia | |
| CCRF-CEM | 65.06 |
| HL-60(TB) | 46.93 |
| K-562 | 26.09 |
| MOLT-4 | 23.52 |
| RPMI-8226 | 31.65 |
| SR | 25.88 |
| Non-Small Cell Lung Cancer | |
| A549/ATCC | 40.60 |
| EKVX | 45.49 |
| HOP-62 | 76.15 |
| HOP-92 | 56.48 |
| NCI-H226 | 68.43 |
| NCI-H23 | 49.03 |
| NCI-H322M | 72.06 |
| NCI-H460 | 33.43 |
| NCI-H522 | 27.14 |
| Colon Cancer | |
| COLO 205 | 44.66 |
| HCC-2998 | 65.91 |
| HCT-116 | 30.26 |
| HCT-15 | 22.91 |
| HT29 | 47.76 |
| KM12 | 35.50 |
| SW-620 | 52.24 |
| CNS Cancer | |
| SF-268 | 51.04 |
| SF-295 | 24.80 |
| SF-539 | 67.76 |
| SNB-19 | 52.36 |
| SNB-75 | 52.49 |
| U251 | 43.88 |
| Melanoma | |
| LOX IMVI | 50.25 |
| MALME-3M | 92.76 |
| M14 | 31.22 |
| MDA-MB-435 | 54.51 |
| SK-MEL-2 | 43.39 |
| SK-MEL-28 | 63.76 |
| SK-MEL-5 | 50.81 |
| UACC-257 | 59.05 |
| UACC-62 | 26.05 |
| Ovarian Cancer | |
| IGROV1 | 65.65 |
| OVCAR-3 | 40.77 |
| OVCAR-4 | 35.17 |
| OVCAR-5 | 83.52 |
| OVCAR-8 | 55.54 |
| NCI/ADR-RES | 38.76 |
| SK-OV-3 | 87.71 |
| Renal Cancer | |
| 786-0 | 61.35 |
| ACHN | 28.06 |
| CAKI-1 | 39.41 |
| SN12C | 46.41 |
| TK-10 | 48.65 |
| UO-31 | 42.67 |
| Prostate Cancer | |
| PC-3 | 23.48 |
| DU-145 | 63.69 |
| Breast Cancer | |
| MCF7 | 43.28 |
| MDA-MB-231/ATCC | 46.16 |
| HS 578T | 59.82 |
| BT-549 | 61.34 |
| T-47D | 28.71 |
| MDA-MB-468 | 35.94 |
| Mean | 48.06 |
| Delta | 25.15 |
| Range | 69.85 |

**Mean Growth Percent - Growth Percent**

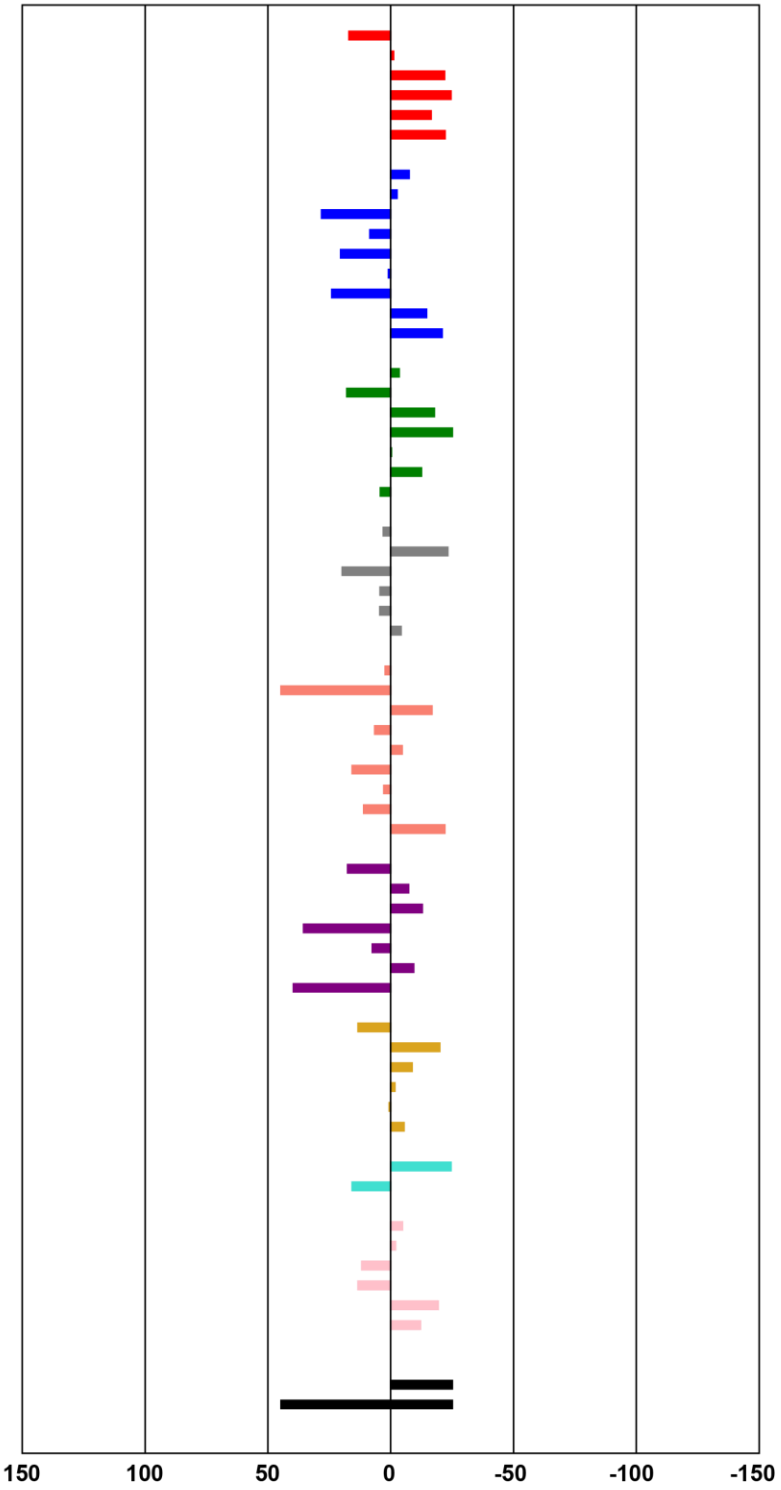


**Figure S8.** Mean growth percentages expressed by compound **10** (NSC 785397) at a single-dose concentration of 10 µM over the NCI-60 cancer cell lines. Zero on the X-axis represents the mean percentage of growth of the tested cell lines. The percentage of growth of each cell line relative to the mean is represented by a horizontal bar extending to the right side indicating more sensitivity or to the left side indicating less sensitivity.

| Developmental Therapeutics Program One Dose Mean Graph | NSC: D-784261 / 1 | Conc: 1.00E-5 Molar | Test Date: Apr 27, 2015 |
|---|---|---|---|
| | Experiment ID: 1504OS80 | | Report Date: May 21, 2015 |

| Panel/Cell Line | Growth Percent |
|---|---|
| Leukemia | |
| CCRF-CEM | 43.90 |
| HL-60(TB) | 29.83 |
| K-562 | 24.30 |
| RPMI-8226 | 26.03 |
| SR | 48.34 |
| Non-Small Cell Lung Cancer | |
| A549/ATCC | 14.24 |
| EKVX | 36.13 |
| HOP-62 | 50.14 |
| HOP-92 | 47.71 |
| NCI-H226 | 38.70 |
| NCI-H23 | 34.19 |
| NCI-H322M | 77.22 |
| NCI-H460 | 18.77 |
| NCI-H522 | 12.95 |
| Colon Cancer | |
| COLO 205 | -9.64 |
| HCC-2998 | 40.02 |
| HCT-116 | 5.18 |
| HCT-15 | 21.46 |
| HT29 | 23.78 |
| KM12 | 28.42 |
| SW-620 | 29.12 |
| CNS Cancer | |
| SF-268 | 42.16 |
| SF-295 | 32.06 |
| SF-539 | 53.71 |
| SNB-19 | 37.40 |
| SNB-75 | 33.47 |
| U251 | 27.06 |
| Melanoma | |
| LOX IMVI | 33.53 |
| MALME-3M | 65.66 |
| M14 | 19.55 |
| MDA-MB-435 | 50.55 |
| SK-MEL-2 | 43.03 |
| SK-MEL-28 | 65.47 |
| SK-MEL-5 | 51.94 |
| UACC-257 | 37.81 |
| UACC-62 | 42.14 |
| Ovarian Cancer | |
| IGROV1 | 46.35 |
| OVCAR-3 | 39.08 |
| OVCAR-4 | 38.12 |
| OVCAR-5 | 68.80 |
| OVCAR-8 | 41.51 |
| NCI/ADR-RES | 33.99 |
| SK-OV-3 | 42.34 |
| Renal Cancer | |
| 786-0 | 37.35 |
| A498 | 60.46 |
| ACHN | 23.48 |
| CAKI-1 | 44.61 |
| RXF 393 | 55.82 |
| SN12C | 43.58 |
| TK-10 | 36.76 |
| UO-31 | 34.06 |
| Prostate Cancer | |
| PC-3 | 13.27 |
| DU-145 | 42.64 |
| Breast Cancer | |
| MCF7 | 22.66 |
| MDA-MB-231/ATCC | 46.79 |
| HS 578T | 65.06 |
| BT-549 | 32.35 |
| T-47D | 39.05 |
| MDA-MB-468 | 33.55 |
| Mean | 37.59 |
| Delta | 47.23 |
| Range | 86.86 |

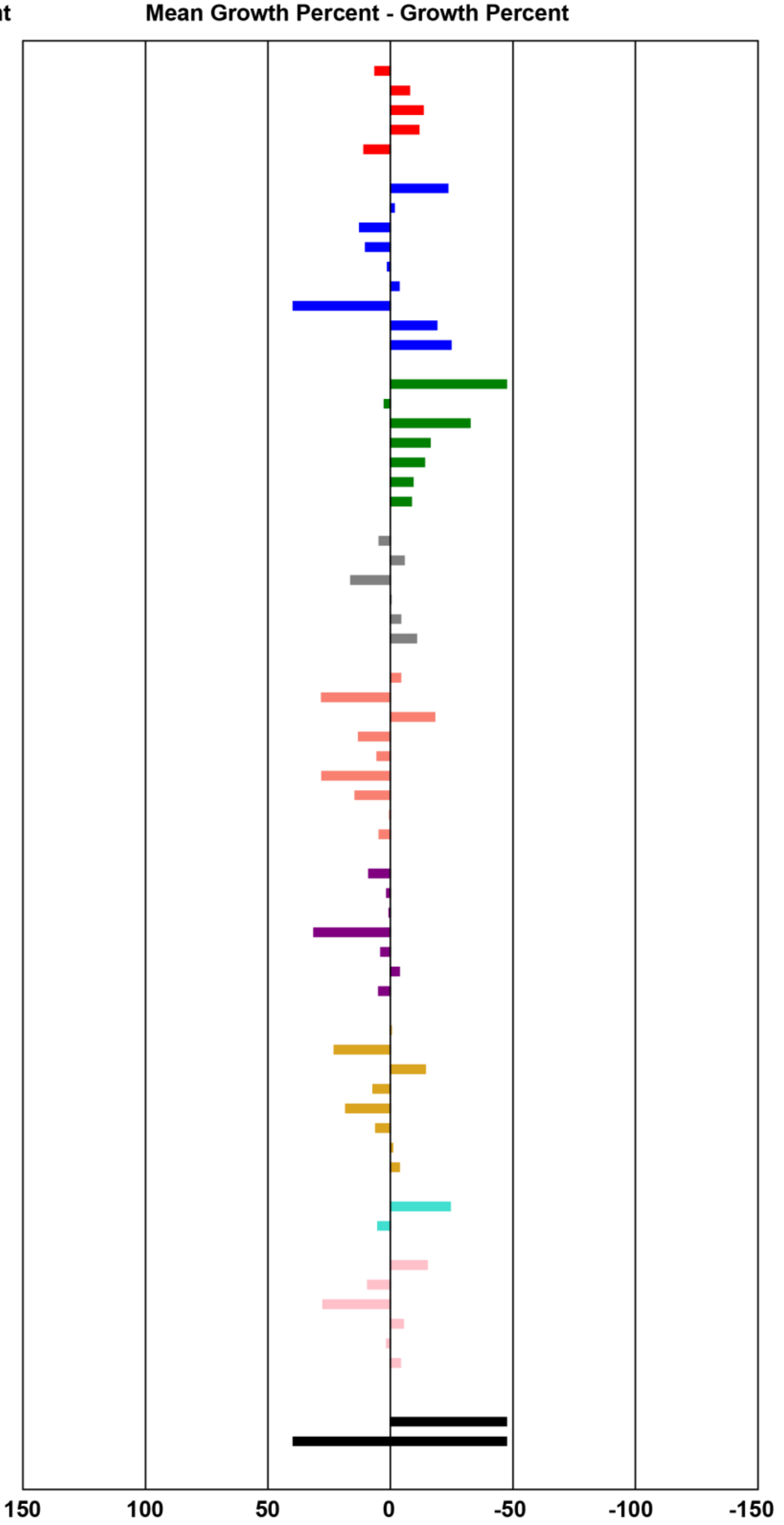


**Figure S9.** Mean growth percentages expressed by compound **11** (NSC 784261) at a single-dose concentration of 10 μM over the NCI-60 cancer cell lines. Zero on the X-axis represents the mean percentage of growth of the tested cell lines. The percentage of growth of each cell line relative to the mean is represented by a horizontal bar extending to the right side indicating more sensitivity or to the left side indicating less sensitivity.

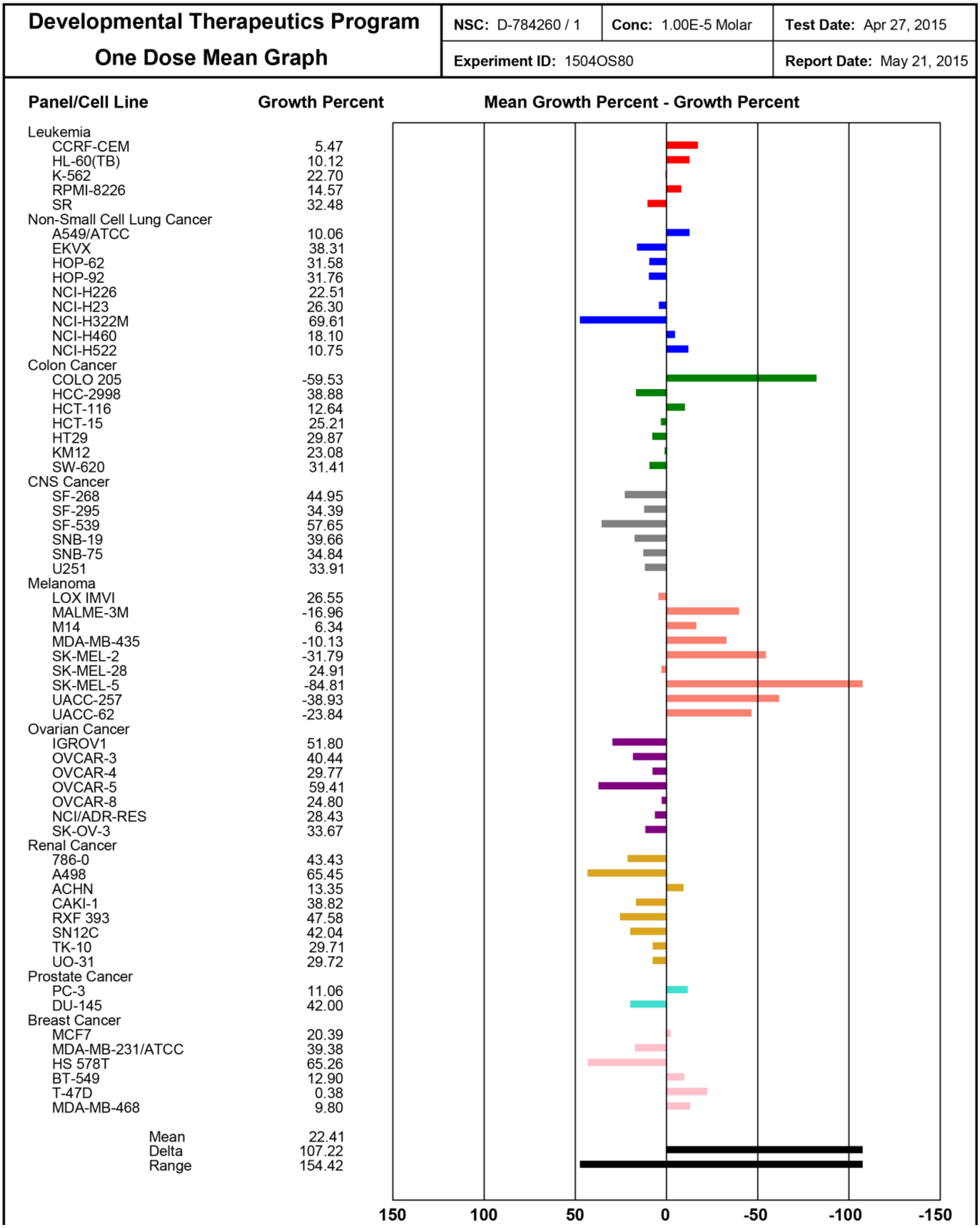


**Figure S10.** Mean growth percentages expressed by compound **12** (NSC 784260) at a single-dose concentration of 10 µM over the NCI-60 cancer cell lines. Zero on the X-axis represents the mean percentage of growth of the tested cell lines. The percentage of growth of each cell line relative to the mean is represented by a horizontal bar extending to the right side indicating more sensitivity or to the left side indicating less sensitivity.

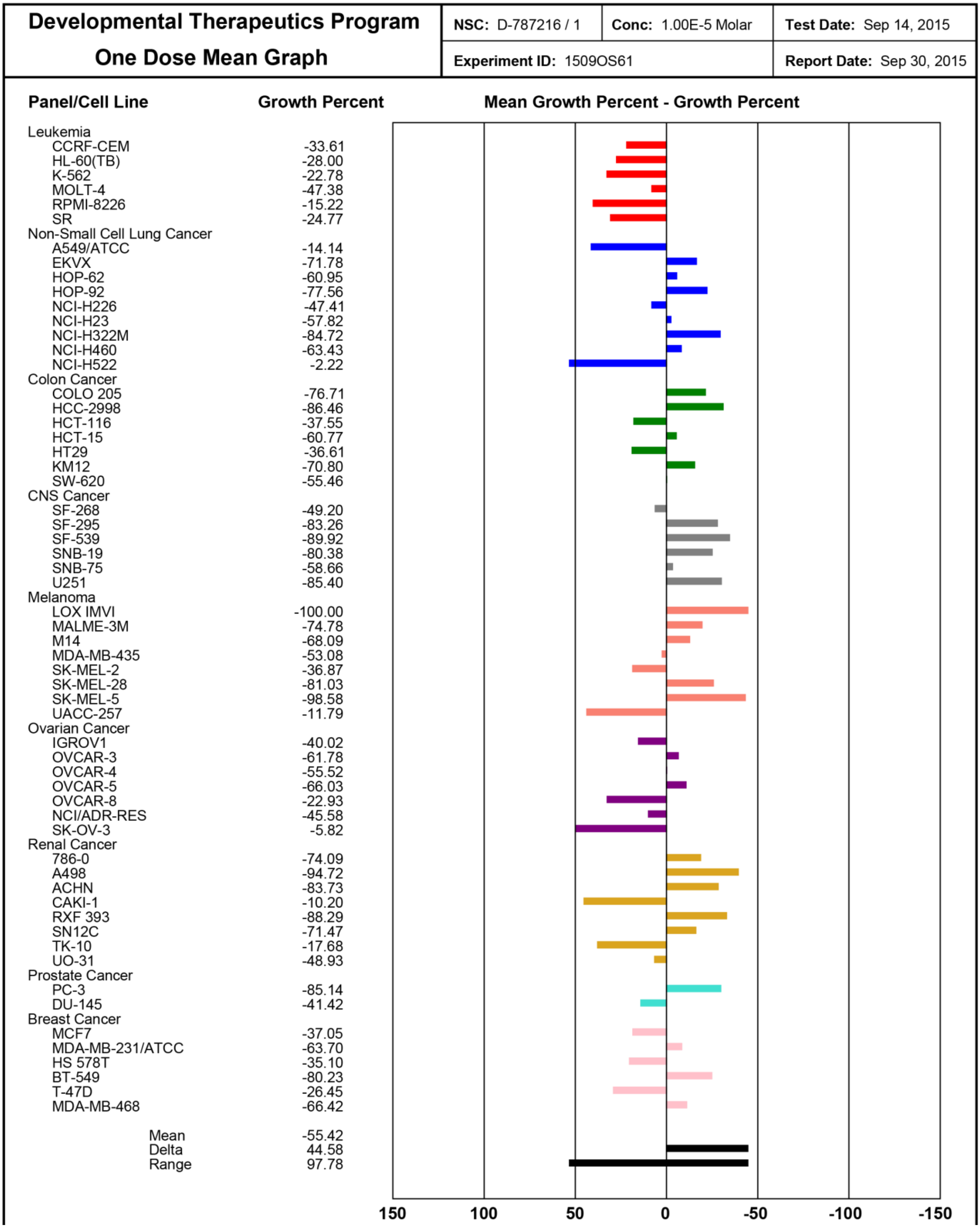


**Figure S11.** Mean growth percentages expressed by compound **14** (NSC 787216) at a single-dose concentration of 10 µM over the NCI-60 cancer cell lines. Zero on the X-axis represents the mean percentage of growth of the tested cell lines. The percentage of growth of each cell line relative to the mean is represented by a horizontal bar extending to the right side indicating more sensitivity or to the left side indicating less sensitivity.

| Developmental Therapeutics Program One Dose Mean Graph | NSC: D-785391 / 1 | Conc: 1.00E-5 Molar | Test Date: Jul 13, 2015 |
|---|---|---|---|
| | Experiment ID: 1507OS19 | | Report Date: Jul 31, 2015 |

| Panel/Cell Line | Growth Percent |
|---|---|
| Leukemia | |
| CCRF-CEM | 18.72 |
| HL-60(TB) | 16.06 |
| K-562 | 16.42 |
| MOLT-4 | 8.61 |
| RPMI-8226 | 12.84 |
| SR | 15.50 |
| Non-Small Cell Lung Cancer | |
| A549/ATCC | 29.11 |
| EKVX | 34.38 |
| HOP-62 | 83.78 |
| HOP-92 | 38.04 |
| NCI-H226 | 58.50 |
| NCI-H23 | 31.69 |
| NCI-H322M | 61.27 |
| NCI-H460 | 17.47 |
| NCI-H522 | 28.11 |
| Colon Cancer | |
| HCC-2998 | 16.11 |
| HCT-116 | 6.65 |
| HCT-15 | 9.41 |
| HT29 | 14.49 |
| KM12 | 21.65 |
| SW-620 | 27.92 |
| CNS Cancer | |
| SF-268 | 48.32 |
| SF-295 | 24.89 |
| SF-539 | 29.85 |
| SNB-19 | 39.45 |
| SNB-75 | 62.61 |
| U251 | 23.44 |
| Melanoma | |
| LOX IMVI | 26.06 |
| MALME-3M | 60.13 |
| M14 | 28.53 |
| MDA-MB-435 | 39.56 |
| SK-MEL-2 | 52.36 |
| SK-MEL-28 | 26.95 |
| SK-MEL-5 | 35.25 |
| UACC-257 | 33.71 |
| UACC-62 | 42.86 |
| Ovarian Cancer | |
| IGROV1 | 45.43 |
| OVCAR-3 | 24.25 |
| OVCAR-4 | 31.73 |
| OVCAR-5 | 62.92 |
| OVCAR-8 | 32.16 |
| NCI/ADR-RES | 31.94 |
| SK-OV-3 | 76.34 |
| Renal Cancer | |
| 786-0 | 45.23 |
| ACHN | 17.13 |
| CAKI-1 | 42.76 |
| SN12C | 37.58 |
| TK-10 | 54.42 |
| UO-31 | 27.76 |
| Prostate Cancer | |
| PC-3 | 16.18 |
| DU-145 | 39.95 |
| Breast Cancer | |
| MCF7 | 18.13 |
| MDA-MB-231/ATCC | 53.18 |
| HS 578T | 47.53 |
| BT-549 | 27.72 |
| T-47D | 20.75 |
| MDA-MB-468 | 19.98 |
| Mean | 33.57 |
| Delta | 26.92 |
| Range | 77.13 |

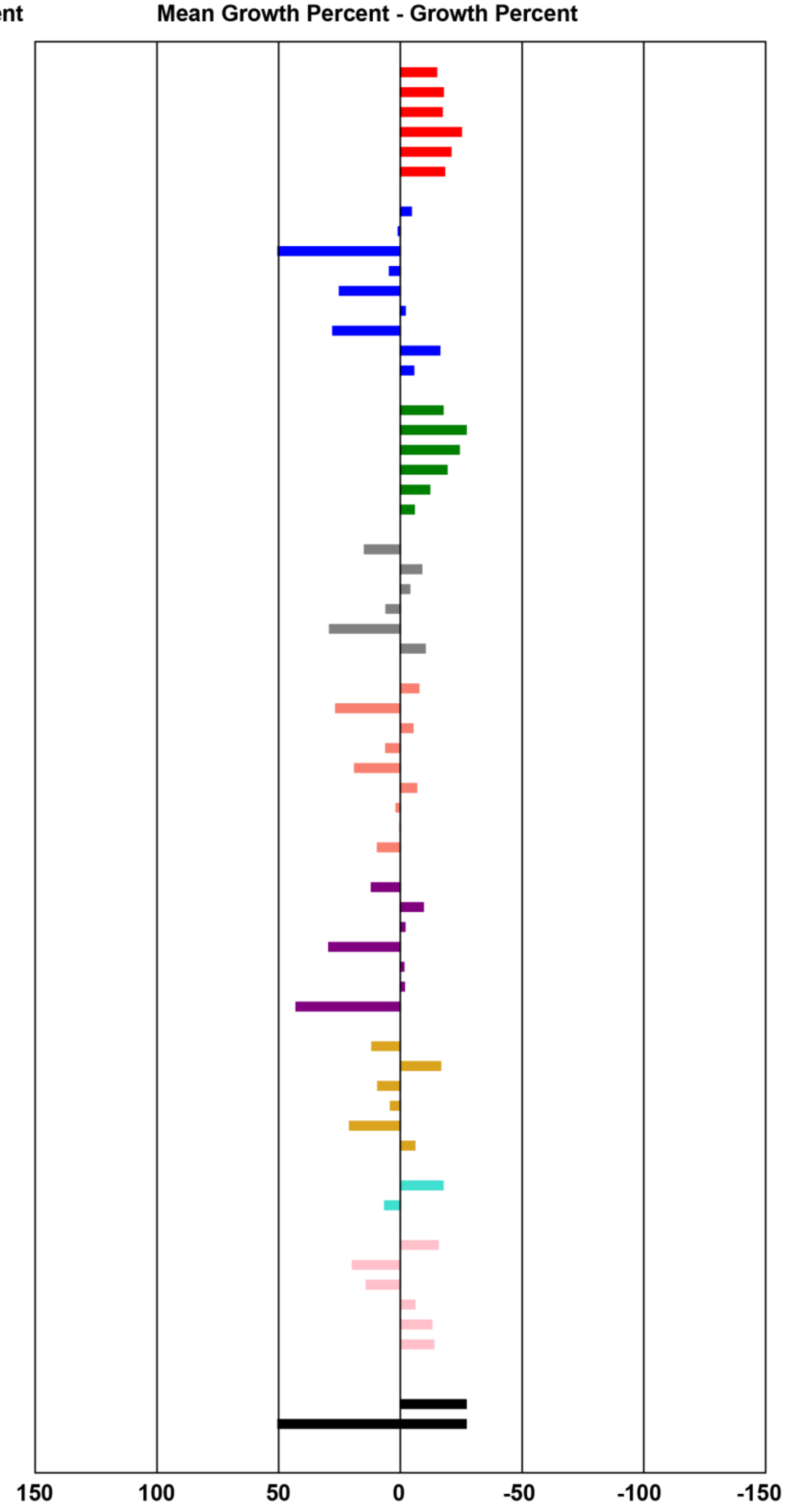


**Figure S12.** Mean growth percentages expressed by compound **15** (NSC 785391) at a single-dose concentration of 10 µM over the NCI-60 cancer cell lines. Zero on the X-axis represents the mean percentage of growth of the tested cell lines. The percentage of growth of each cell line relative to the mean is represented by a horizontal bar extending to the right side indicating more sensitivity or to the left side indicating less sensitivity.

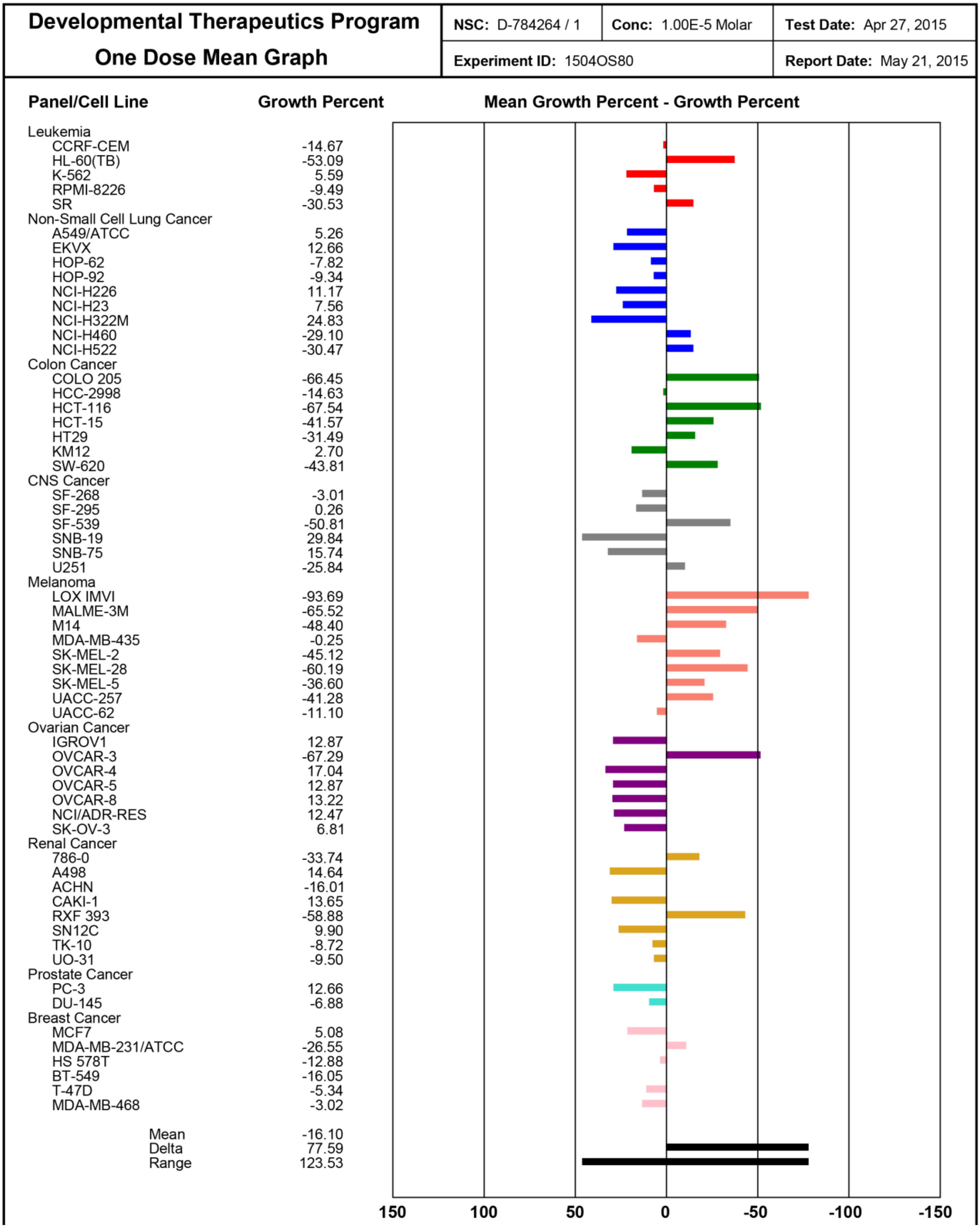


**Figure S13.** Mean growth percentages expressed by compound **16** (NSC 784264) at a single-dose concentration of 10 µM over the NCI-60 cancer cell lines. Zero on the X-axis represents the mean percentage of growth of the tested cell lines. The percentage of growth of each cell line relative to the mean is represented by a horizontal bar extending to the right side indicating more sensitivity or to the left side indicating less sensitivity.

| Developmental Therapeutics Program One Dose Mean Graph | NSC: D-784263 / 1 | Conc: 1.00E-5 Molar | Test Date: Apr 27, 2015 |
|---|---|---|---|
| | Experiment ID: 1504OS80 | | Report Date: May 21, 2015 |

| Panel/Cell Line | Growth Percent |
|---|---|
| Leukemia | |
| CCRF-CEM | 4.27 |
| HL-60(TB) | -9.45 |
| K-562 | 11.09 |
| RPMI-8226 | 4.82 |
| SR | -8.74 |
| Non-Small Cell Lung Cancer | |
| A549/ATCC | 8.76 |
| EKVX | 11.00 |
| HOP-62 | 7.71 |
| HOP-92 | -15.51 |
| NCI-H226 | 19.14 |
| NCI-H23 | 8.23 |
| NCI-H322M | 39.60 |
| NCI-H460 | 2.11 |
| NCI-H522 | -6.14 |
| Colon Cancer | |
| COLO 205 | -55.75 |
| HCC-2998 | -7.04 |
| HCT-116 | -71.31 |
| HCT-15 | -31.85 |
| HT29 | -0.47 |
| KM12 | 8.54 |
| SW-620 | -7.67 |
| CNS Cancer | |
| SF-268 | -10.88 |
| SF-295 | 11.74 |
| SF-539 | -15.34 |
| SNB-19 | 36.49 |
| SNB-75 | 19.98 |
| U251 | -4.29 |
| Melanoma | |
| LOX IMVI | -86.27 |
| MALME-3M | -43.49 |
| M14 | -33.93 |
| MDA-MB-435 | -22.27 |
| SK-MEL-2 | -42.09 |
| SK-MEL-28 | -45.49 |
| SK-MEL-5 | -87.13 |
| UACC-257 | -34.57 |
| UACC-62 | -11.11 |
| Ovarian Cancer | |
| IGROV1 | 26.54 |
| OVCAR-3 | -92.62 |
| OVCAR-4 | 18.62 |
| OVCAR-5 | 8.46 |
| OVCAR-8 | 19.60 |
| NCI/ADR-RES | 23.71 |
| SK-OV-3 | 27.85 |
| Renal Cancer | |
| 786-0 | -8.17 |
| A498 | 22.23 |
| ACHN | -33.92 |
| CAKI-1 | 14.32 |
| RXF 393 | -56.30 |
| SN12C | 17.06 |
| TK-10 | 6.65 |
| UO-31 | -55.33 |
| Prostate Cancer | |
| PC-3 | 14.06 |
| DU-145 | 10.68 |
| Breast Cancer | |
| MCF7 | 11.44 |
| MDA-MB-231/ATCC | -8.21 |
| HS 578T | 3.14 |
| BT-549 | 5.64 |
| T-47D | -8.10 |
| MDA-MB-468 | 5.01 |
| Mean | -8.22 |
| Delta | 84.40 |
| Range | 132.22 |

Mean Growth Percent - Growth Percent

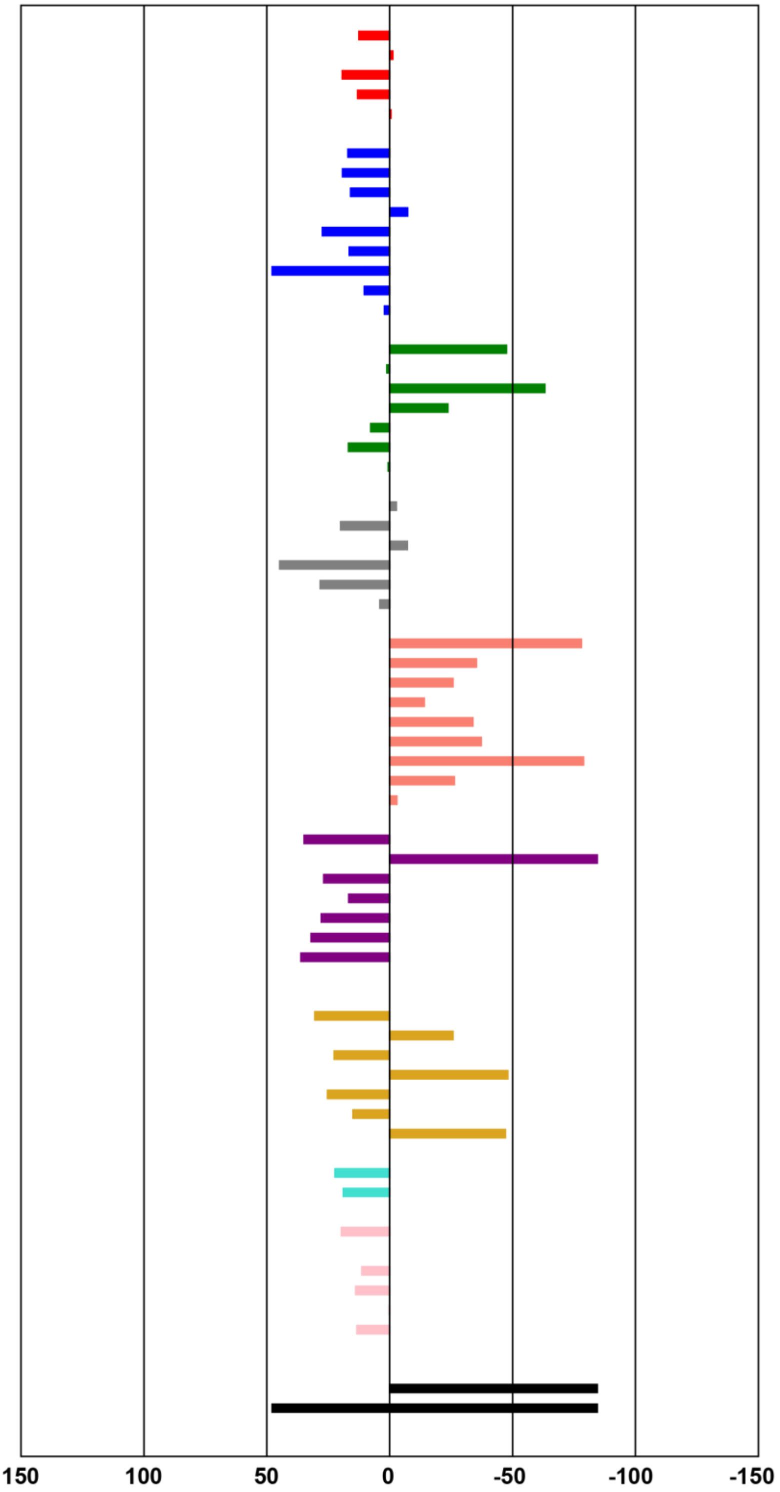


**Figure S14.** Mean growth percentages expressed by compound **17** (NSC 784263) at a single-dose concentration of 10 µM over the NCI-60 cancer cell lines. Zero on the X-axis represents the mean percentage of growth of the tested cell lines. The percentage of growth of each cell line relative to the mean is represented by a horizontal bar extending to the right side indicating more sensitivity or to the left side indicating less sensitivity.

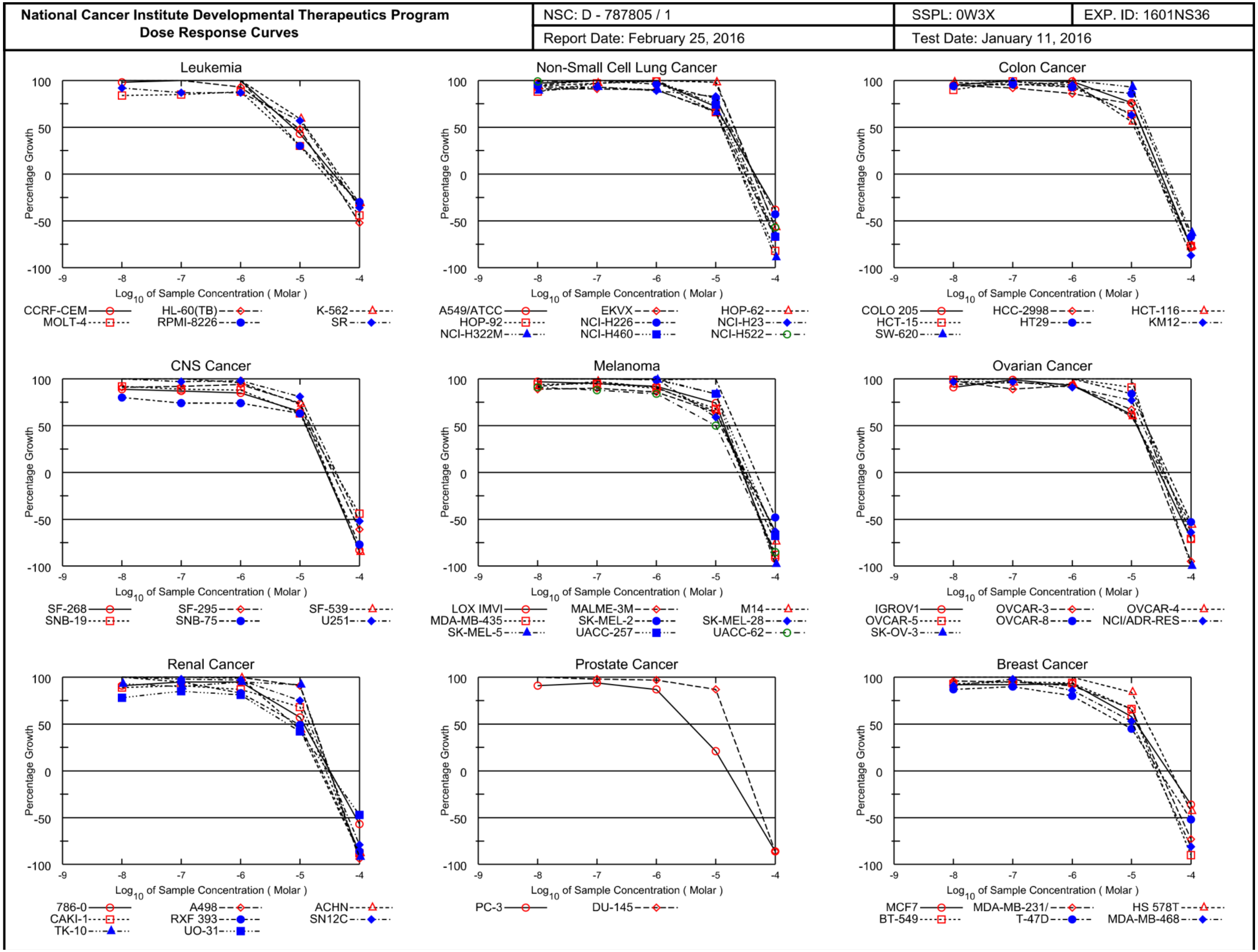


**Figure S15A.** Cytostatic and cytotoxic effects of compound **2** (NSC 787805) on the NCI-60 panel of cancer cell lines at different doses, ranging from 10 nM to 100 µM. Results are displayed as dose-response curves (% growth *versus* sample concentration) for each cell line in the nine cancer subpanels.

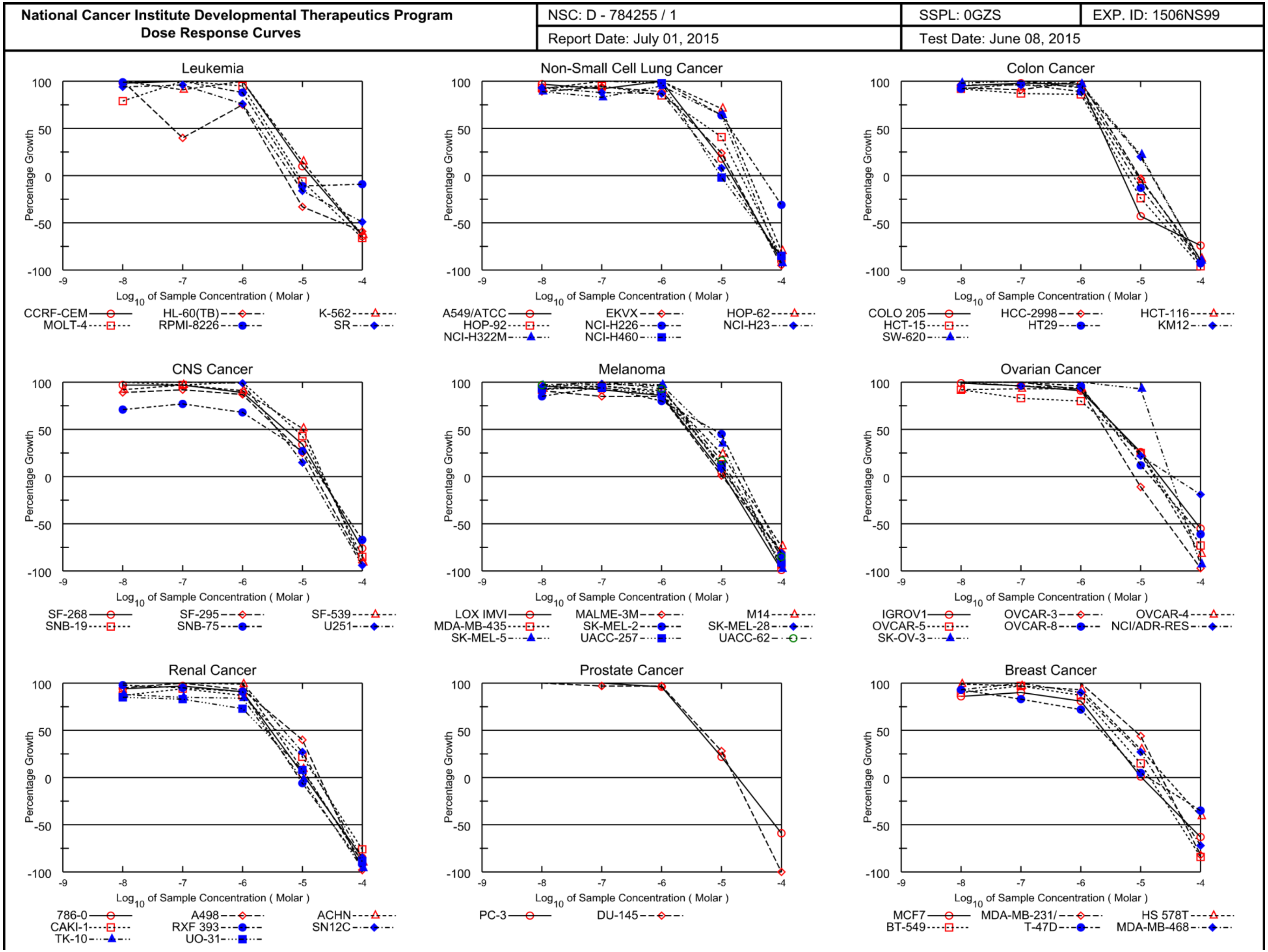


**Figure S16A.** Cytostatic and cytotoxic effects of compound **3** (NSC 784255) on the NCI-60 panel of cancer cell lines at different doses, ranging from 10 nM to 100 µM. Results are displayed as dose-response curves (% growth *versus* sample concentration) for each cell line in the nine cancer subpanels.

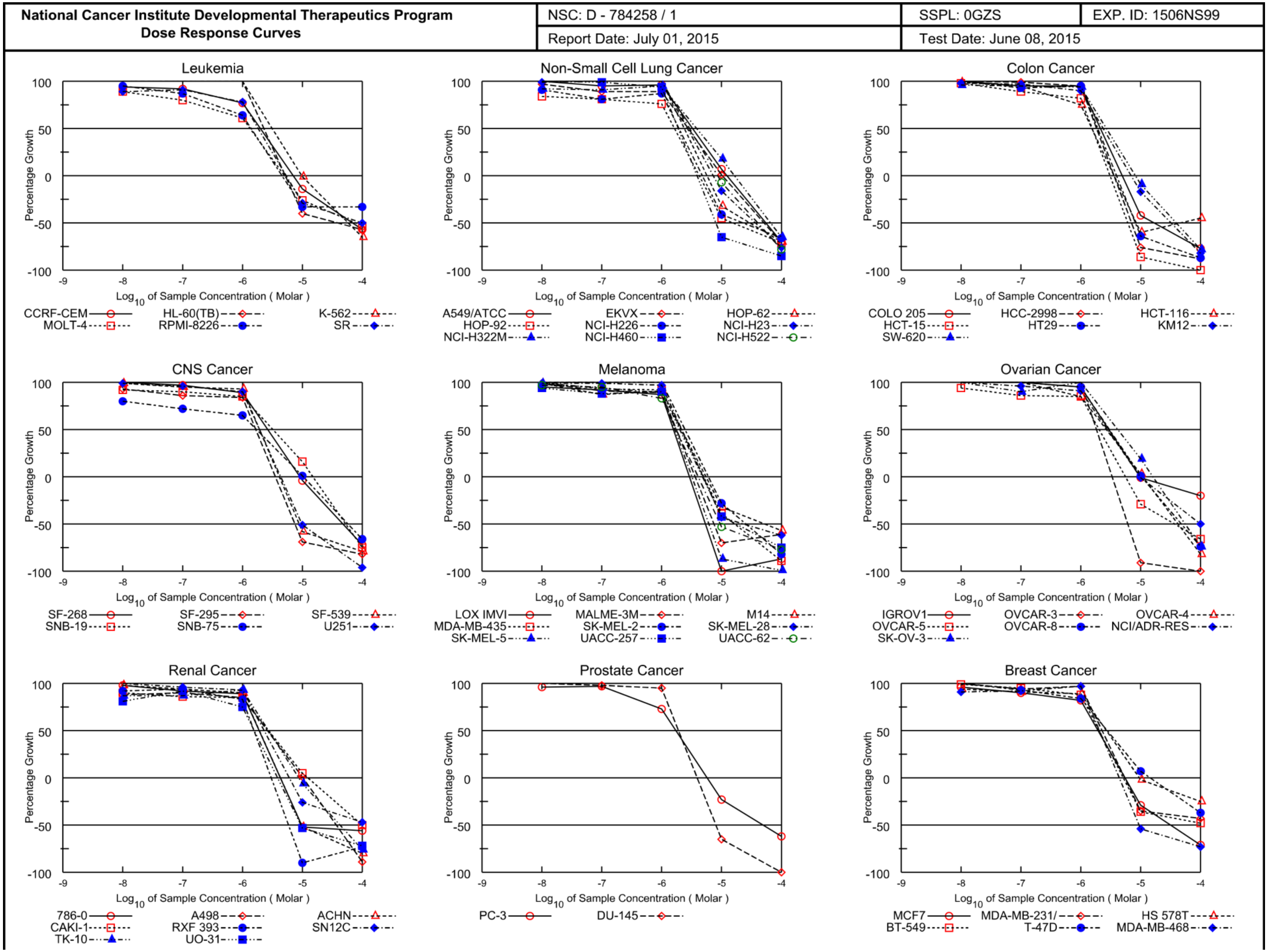


**Figure S17A.** Cytostatic and cytotoxic effects of compound **4** (NSC 784258) on the NCI-60 panel of cancer cell lines at different doses, ranging from 10 nM to 100 µM. Results are displayed as dose-response curves (% growth *versus* sample concentration) for each cell line in the nine cancer subpanels.

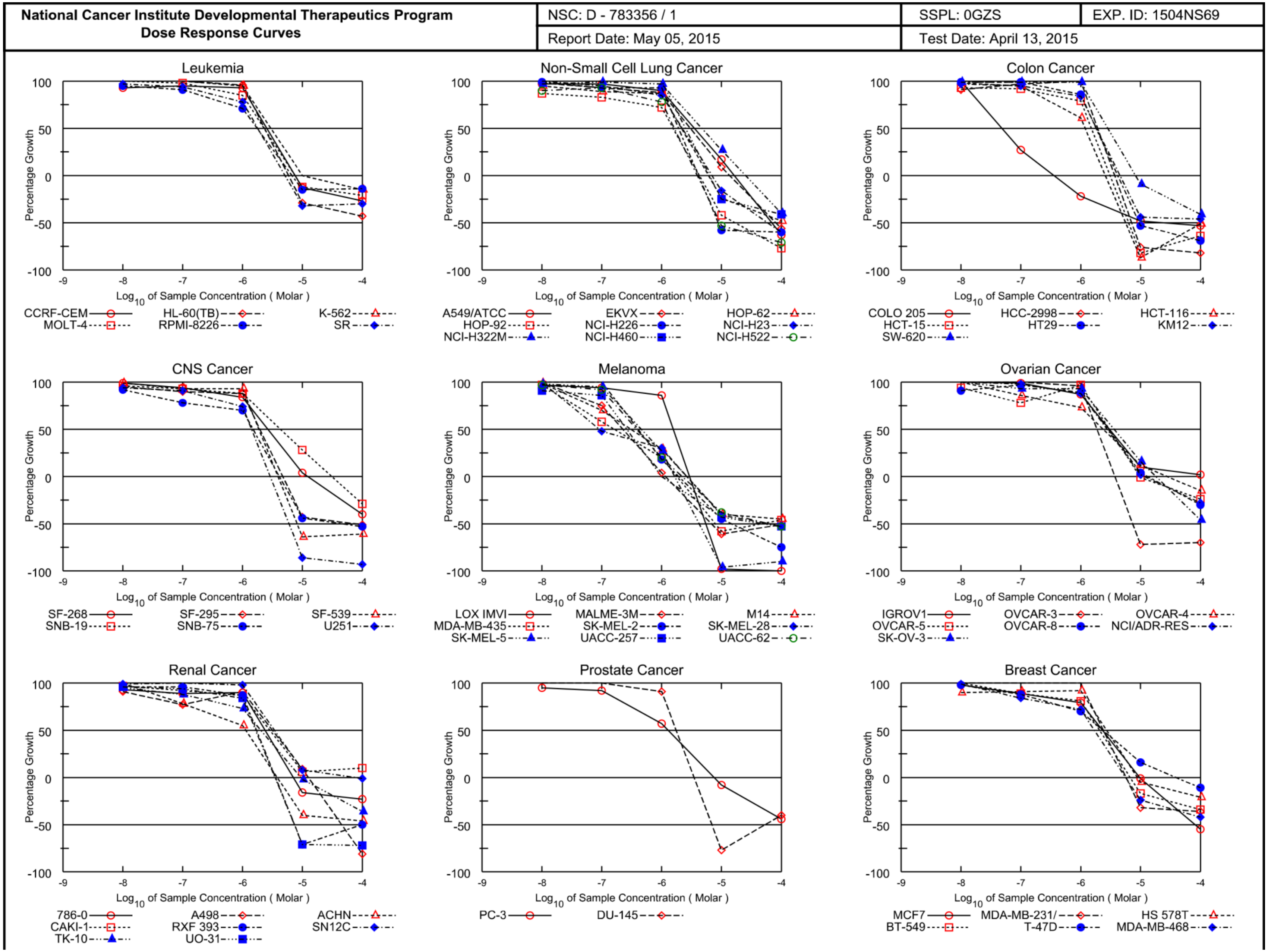


**Figure S18A.** Cytostatic and cytotoxic effects of compound **5** (NSC 783356) on the NCI-60 panel of cancer cell lines at different doses, ranging from 10 nM to 100 μM. Results are displayed as dose-response curves (% growth *versus* sample concentration) for each cell line in the nine cancer subpanels.

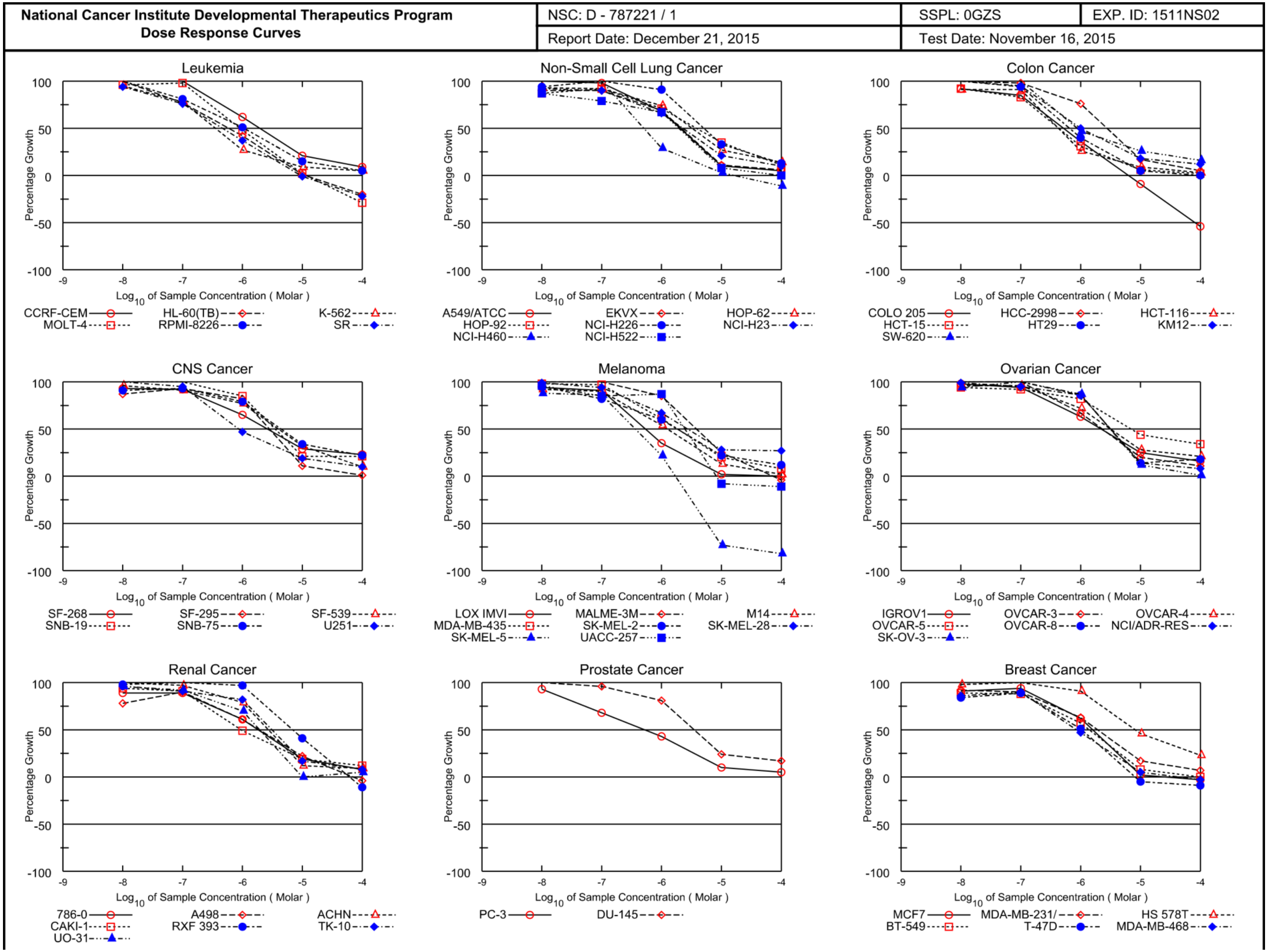


**Figure S19A.** Cytostatic and cytotoxic effects of compound **6** (NSC 787221) on the NCI-60 panel of cancer cell lines at different doses, ranging from 10 nM to 100 µM. Results are displayed as dose-response curves (% growth *versus* sample concentration) for each cell line in the nine cancer subpanels.

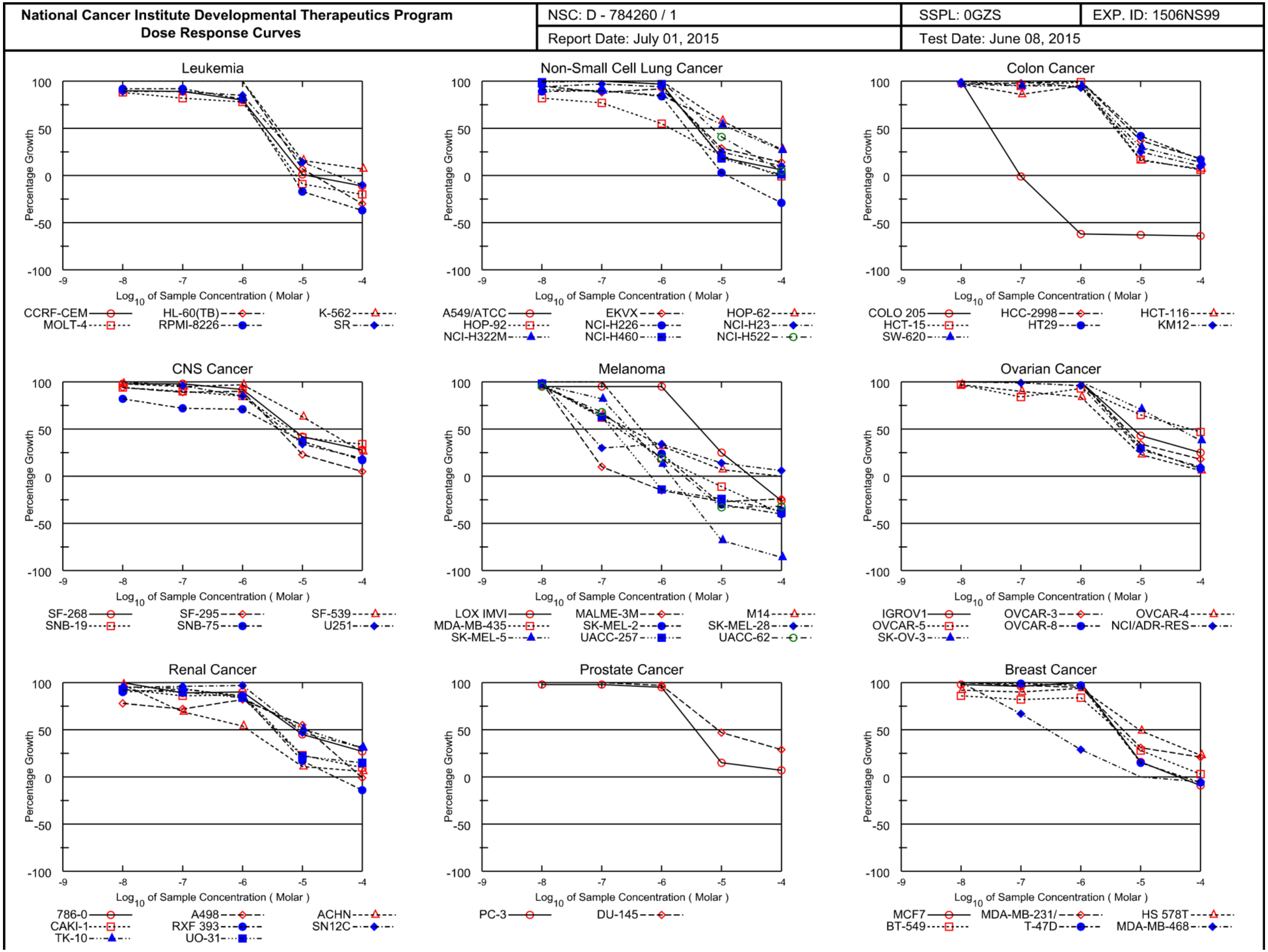


**Figure S20A.**Cytostatic and cytotoxic effects of compound **12** (NSC 784260) on the NCI-60 panel of cancer cell lines at different doses, ranging from 10 nM to 100 µM. Results are displayed as dose-response curves (% growth *versus* sample concentration) for each cell line in the nine cancer subpanels.

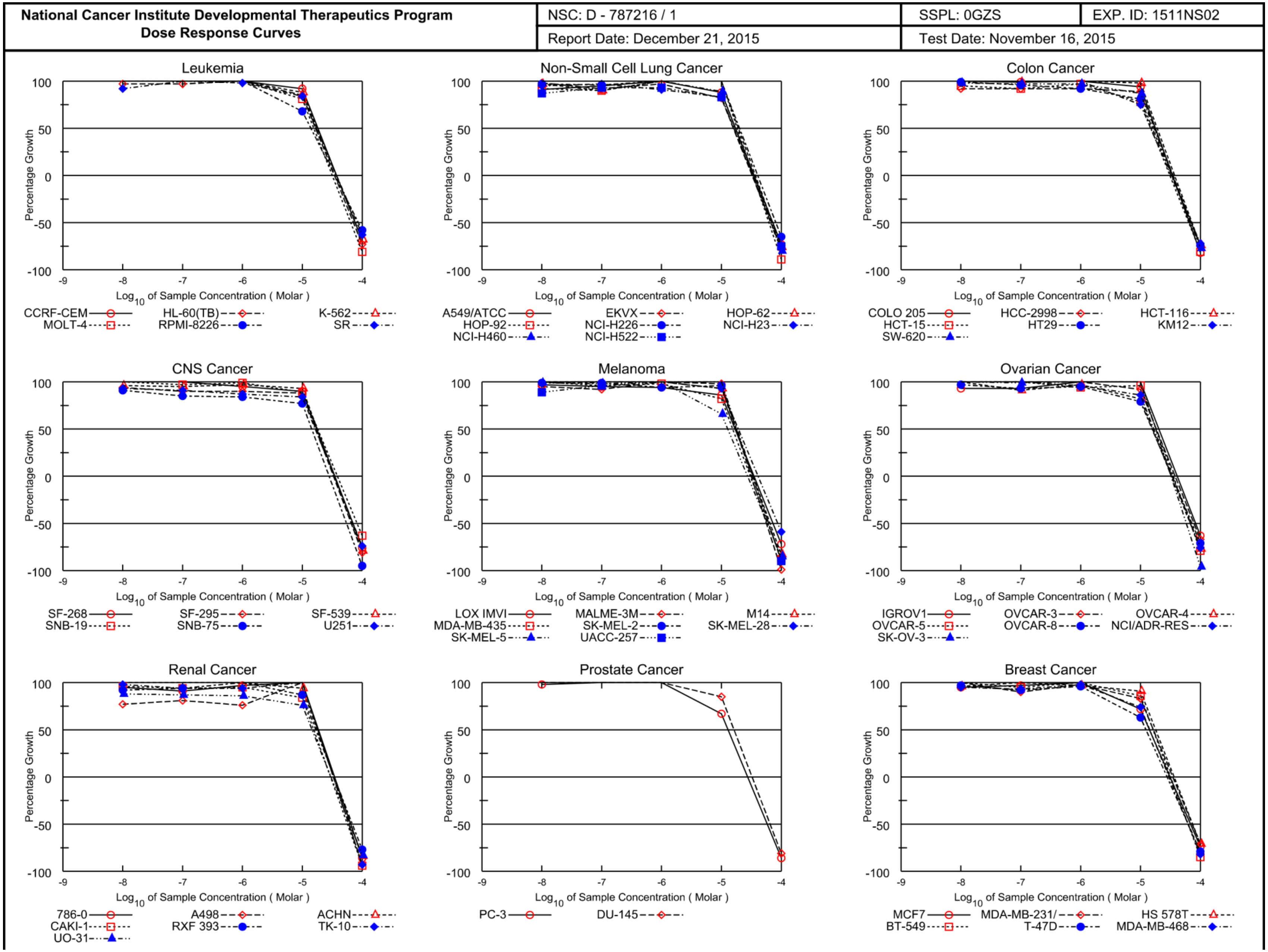


**Figure S21A.** Cytostatic and cytotoxic effects of compound **14** (NSC 787216) on the NCI-60 panel of cancer cell lines at different doses, ranging from 10 nM to 100 µM. Results are displayed as dose-response curves (% growth *versus* sample concentration) for each cell line in the nine cancer subpanels.

| National Cancer Institute Developmental Therapeutics Program Dose Response Curves | NSC: D - 784264 / 1 | SSPL: 0GZS | EXP. ID: 1506NS99 |
|---|---|---|---|
| | Report Date: July 01, 2015 | Test Date: June 08, 2015 | |

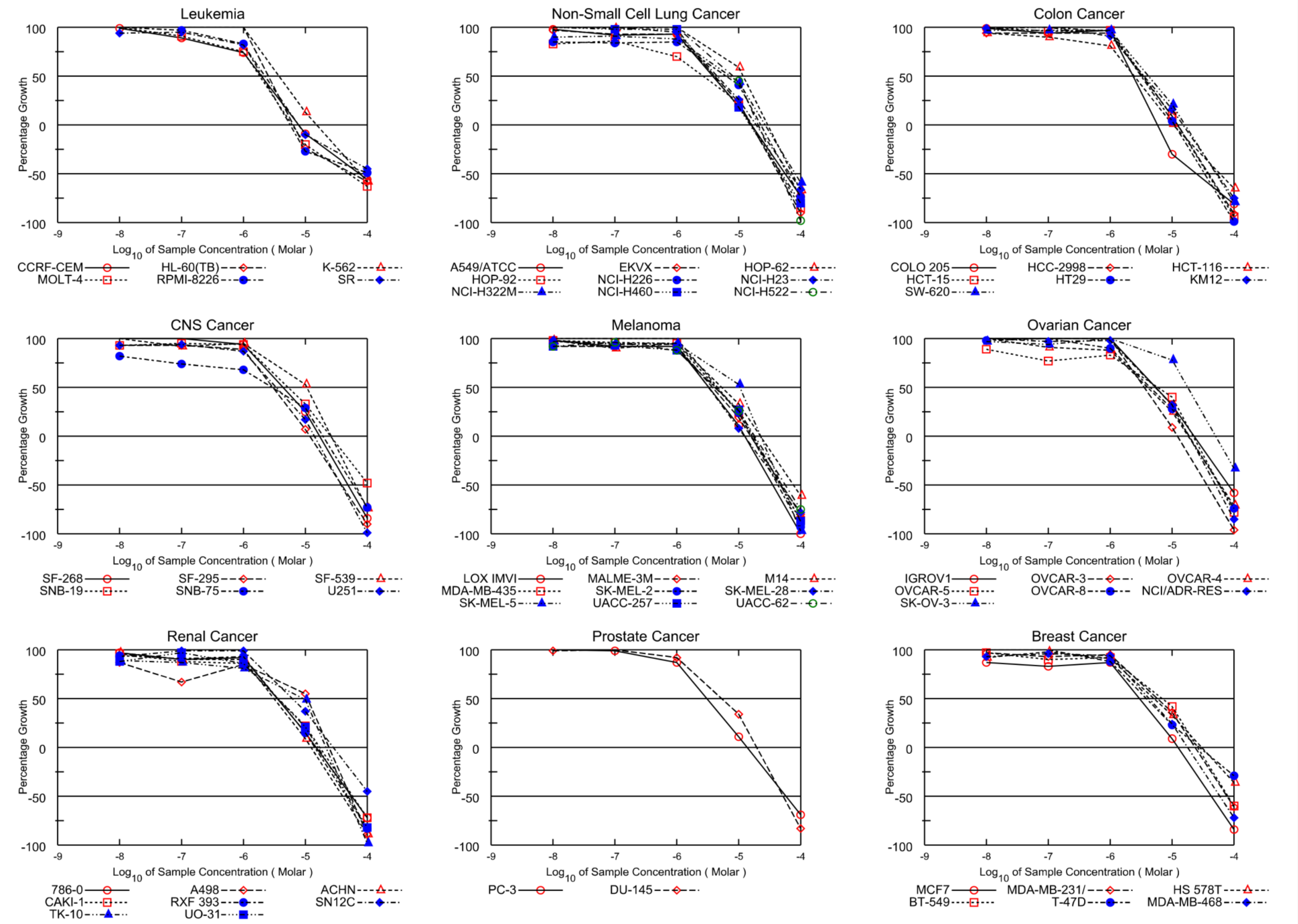


**Figure S22A.** Cytostatic and cytotoxic effects of compound **16** (NSC 784264) on the NCI-60 panel of cancer cell lines at different doses, ranging from 10 nM to 100 µM. Results are displayed as dose-response curves (% growth *versus* sample concentration) for each cell line in the nine cancer subpanels.

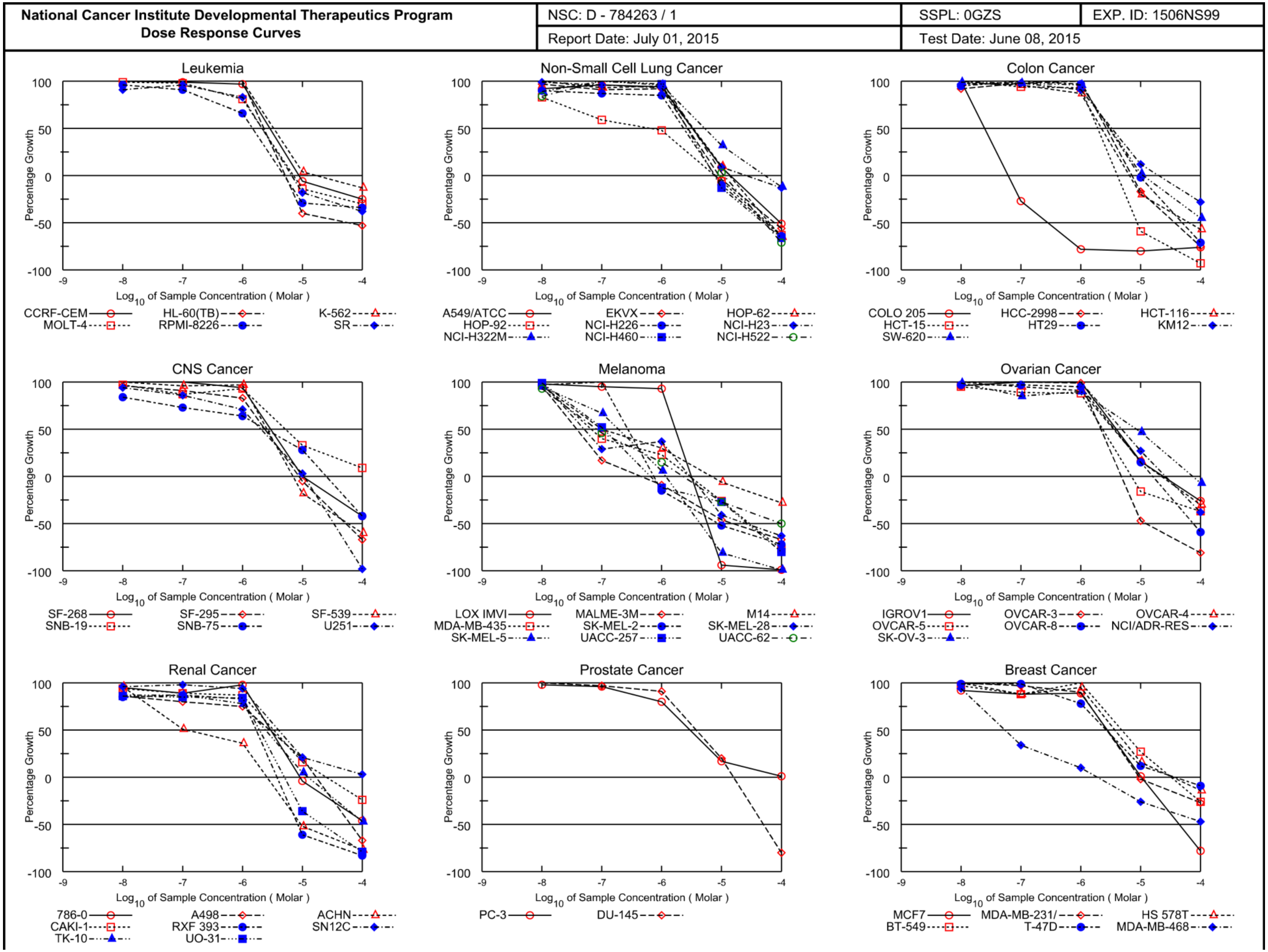


**Figure S23A.** Cytostatic and cytotoxic effects of compound **17** (NSC 784263) on the NCI-60 panel of cancer cell lines at different doses, ranging from 10 nM to 100 µM. Results are displayed as dose-response curves (% growth *versus* sample concentration) for each cell line in the nine cancer subpanels.

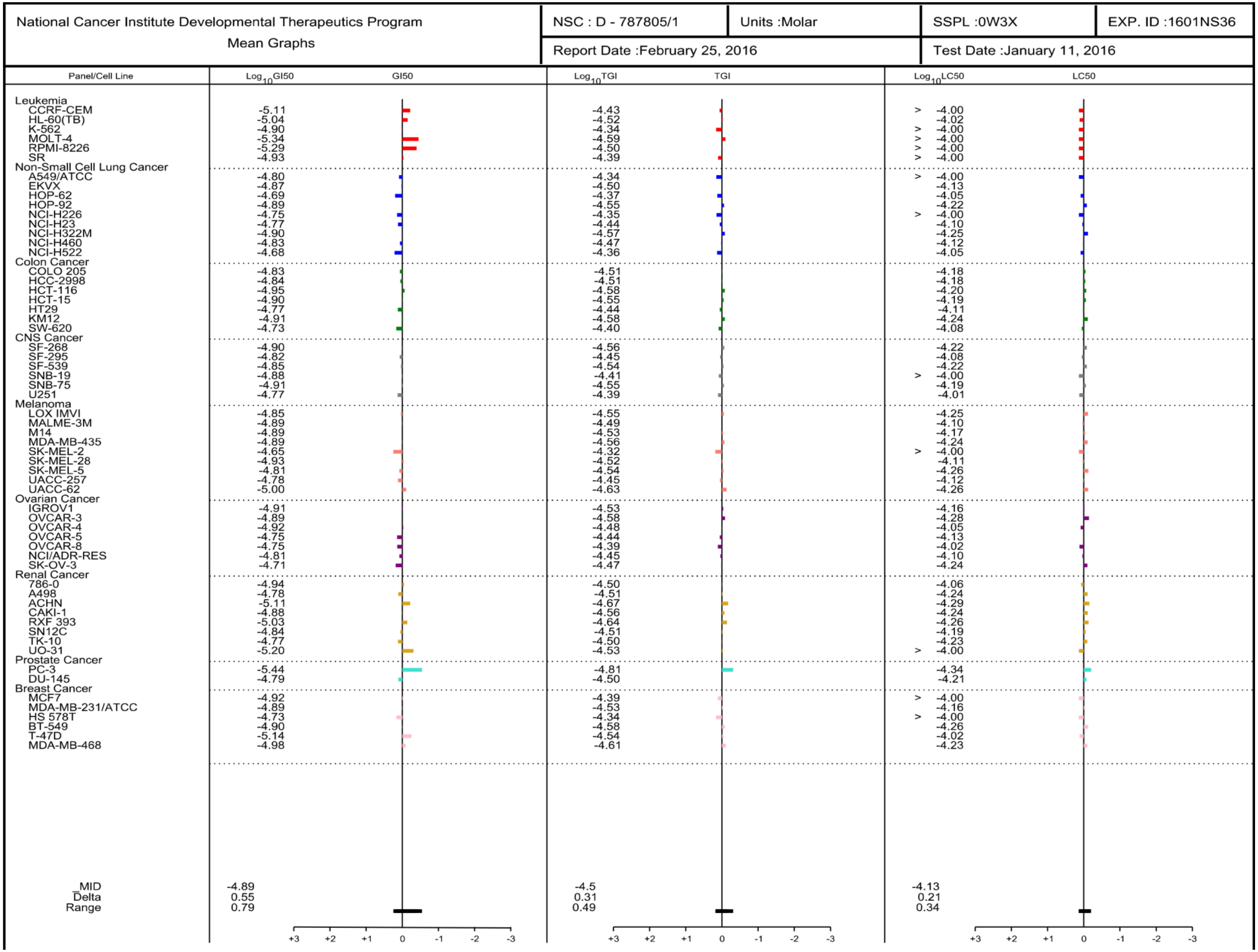


**Figure S15B.** $GI_{50}$ (50% Growth Inhibition), TGI (Total Growth Inhibition) and $LC_{50}$ (50% Lethal Concentration) mean graphs obtained for compound **2** (NSC 787805) tested at five concentrations (0.01, 0.1, 1, 10, 100 μM) against the NCI-60 human cancer cell lines.

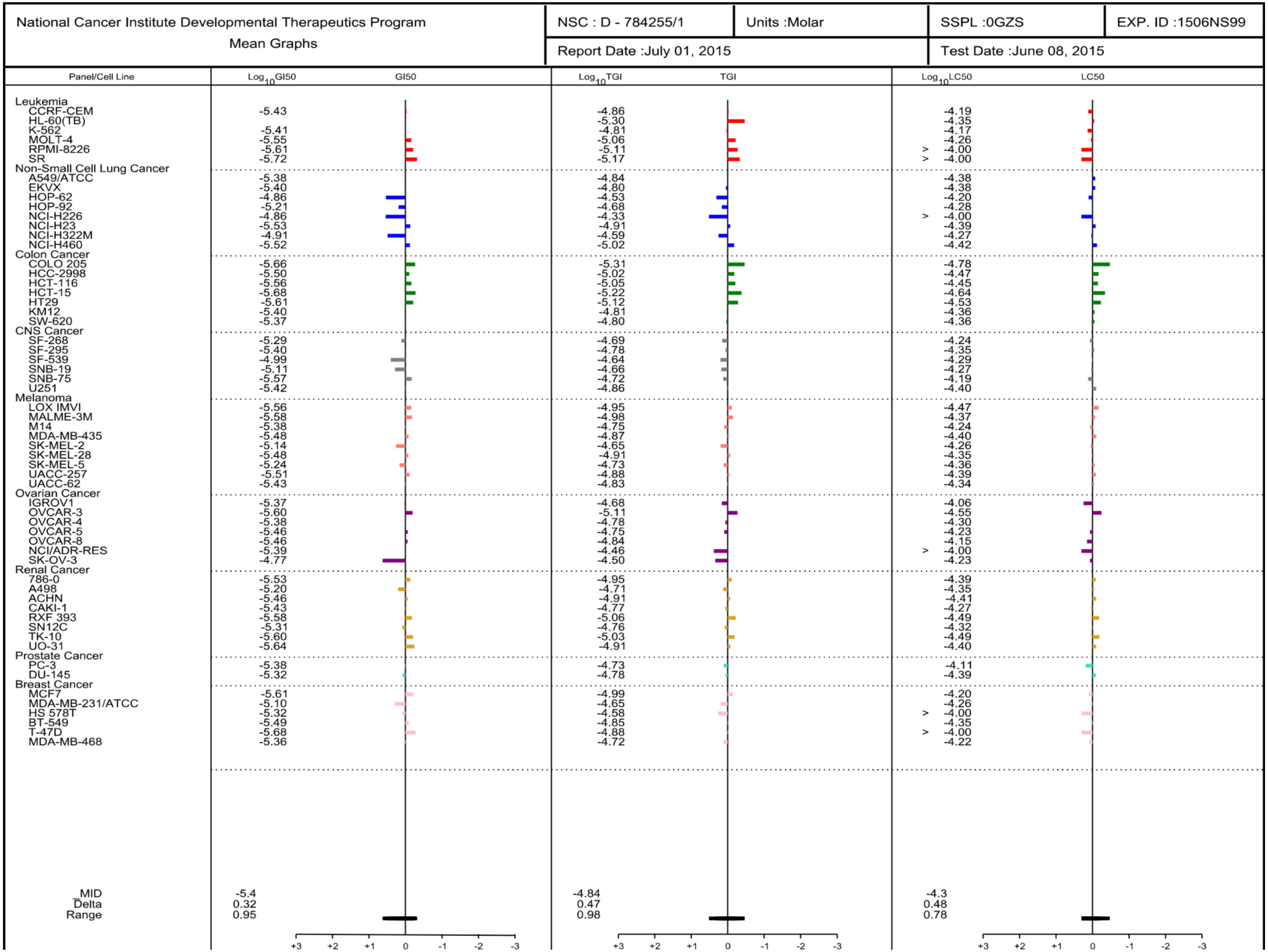

National Cancer Institute Developmental Therapeutics Program
Mean Graphs

NSC : D - 784255/1 | Units :Molar | SSPL :0GZS | EXP. ID :1506NS99
Report Date :July 01, 2015 | Test Date :June 08, 2015

| Panel/Cell Line | $Log_{10}$GI50 | $Log_{10}$TGI | $Log_{10}$LC50 |
|---|---|---|---|
| **Leukemia** | | | |
| CCRF-CEM | -5.43 | -4.86 | -4.19 |
| HL-60(TB) | | -5.30 | -4.35 |
| K-562 | -5.41 | -4.81 | -4.17 |
| MOLT-4 | -5.55 | -5.06 | -4.26 |
| RPMI-8226 | -5.61 | -5.11 | > -4.00 |
| SR | -5.72 | -5.17 | > -4.00 |
| **Non-Small Cell Lung Cancer** | | | |
| A549/ATCC | -5.38 | -4.84 | -4.38 |
| EKVX | -5.40 | -4.80 | -4.38 |
| HOP-62 | -4.86 | -4.53 | -4.20 |
| HOP-92 | -5.21 | -4.68 | -4.28 |
| NCI-H226 | -4.86 | -4.33 | > -4.00 |
| NCI-H23 | -5.53 | -4.91 | -4.39 |
| NCI-H322M | -4.91 | -4.59 | -4.27 |
| NCI-H460 | -5.52 | -5.02 | -4.42 |
| **Colon Cancer** | | | |
| COLO 205 | -5.66 | -5.31 | -4.78 |
| HCC-2998 | -5.50 | -5.02 | -4.47 |
| HCT-116 | -5.56 | -5.05 | -4.45 |
| HCT-15 | -5.68 | -5.22 | -4.64 |
| HT29 | -5.61 | -5.12 | -4.53 |
| KM12 | -5.40 | -4.81 | -4.36 |
| SW-620 | -5.37 | -4.80 | -4.36 |
| **CNS Cancer** | | | |
| SF-268 | -5.29 | -4.69 | -4.24 |
| SF-295 | -5.40 | -4.78 | -4.35 |
| SF-539 | -4.99 | -4.64 | -4.29 |
| SNB-19 | -5.11 | -4.66 | -4.27 |
| SNB-75 | -5.57 | -4.72 | -4.19 |
| U251 | -5.42 | -4.86 | -4.40 |
| **Melanoma** | | | |
| LOX IMVI | -5.56 | -4.95 | -4.47 |
| MALME-3M | -5.58 | -4.98 | -4.37 |
| M14 | -5.38 | -4.75 | -4.24 |
| MDA-MB-435 | -5.48 | -4.87 | -4.40 |
| SK-MEL-2 | -5.14 | -4.65 | -4.26 |
| SK-MEL-28 | -5.48 | -4.91 | -4.35 |
| SK-MEL-5 | -5.24 | -4.73 | -4.36 |
| UACC-257 | -5.51 | -4.88 | -4.39 |
| UACC-62 | -5.43 | -4.83 | -4.34 |
| **Ovarian Cancer** | | | |
| IGROV1 | -5.37 | -4.68 | -4.06 |
| OVCAR-3 | -5.60 | -5.11 | -4.55 |
| OVCAR-4 | -5.38 | -4.78 | -4.30 |
| OVCAR-5 | -5.46 | -4.75 | -4.23 |
| OVCAR-8 | -5.46 | -4.84 | -4.15 |
| NCI/ADR-RES | -5.39 | -4.46 | > -4.00 |
| SK-OV-3 | -4.77 | -4.50 | -4.23 |
| **Renal Cancer** | | | |
| 786-0 | -5.53 | -4.95 | -4.39 |
| A498 | -5.20 | -4.71 | -4.35 |
| ACHN | -5.46 | -4.91 | -4.41 |
| CAKI-1 | -5.43 | -4.77 | -4.27 |
| RXF 393 | -5.58 | -5.06 | -4.49 |
| SN12C | -5.31 | -4.76 | -4.32 |
| TK-10 | -5.60 | -5.03 | -4.49 |
| UO-31 | -5.64 | -4.91 | -4.40 |
| **Prostate Cancer** | | | |
| PC-3 | -5.38 | -4.73 | -4.11 |
| DU-145 | -5.32 | -4.78 | -4.39 |
| **Breast Cancer** | | | |
| MCF7 | -5.61 | -4.99 | -4.20 |
| MDA-MB-231/ATCC | -5.10 | -4.65 | -4.26 |
| HS 578T | -5.32 | -4.58 | > -4.00 |
| BT-549 | -5.49 | -4.85 | -4.35 |
| T-47D | -5.68 | -4.88 | > -4.00 |
| MDA-MB-468 | -5.36 | -4.72 | -4.22 |
| MID | -5.4 | -4.84 | -4.3 |
| Delta | 0.32 | 0.47 | 0.48 |
| Range | 0.95 | 0.98 | 0.78 |

**Figure S16B.** $GI_{50}$ (50% Growth Inhibition), TGI (Total Growth Inhibition) and $LC_{50}$ (50% Lethal Concentration) mean graphs obtained for compound **3** (NSC 784255) tested at five concentrations (0.01, 0.1, 1, 10, 100 μM) against the NCI-60 human cancer cell lines.

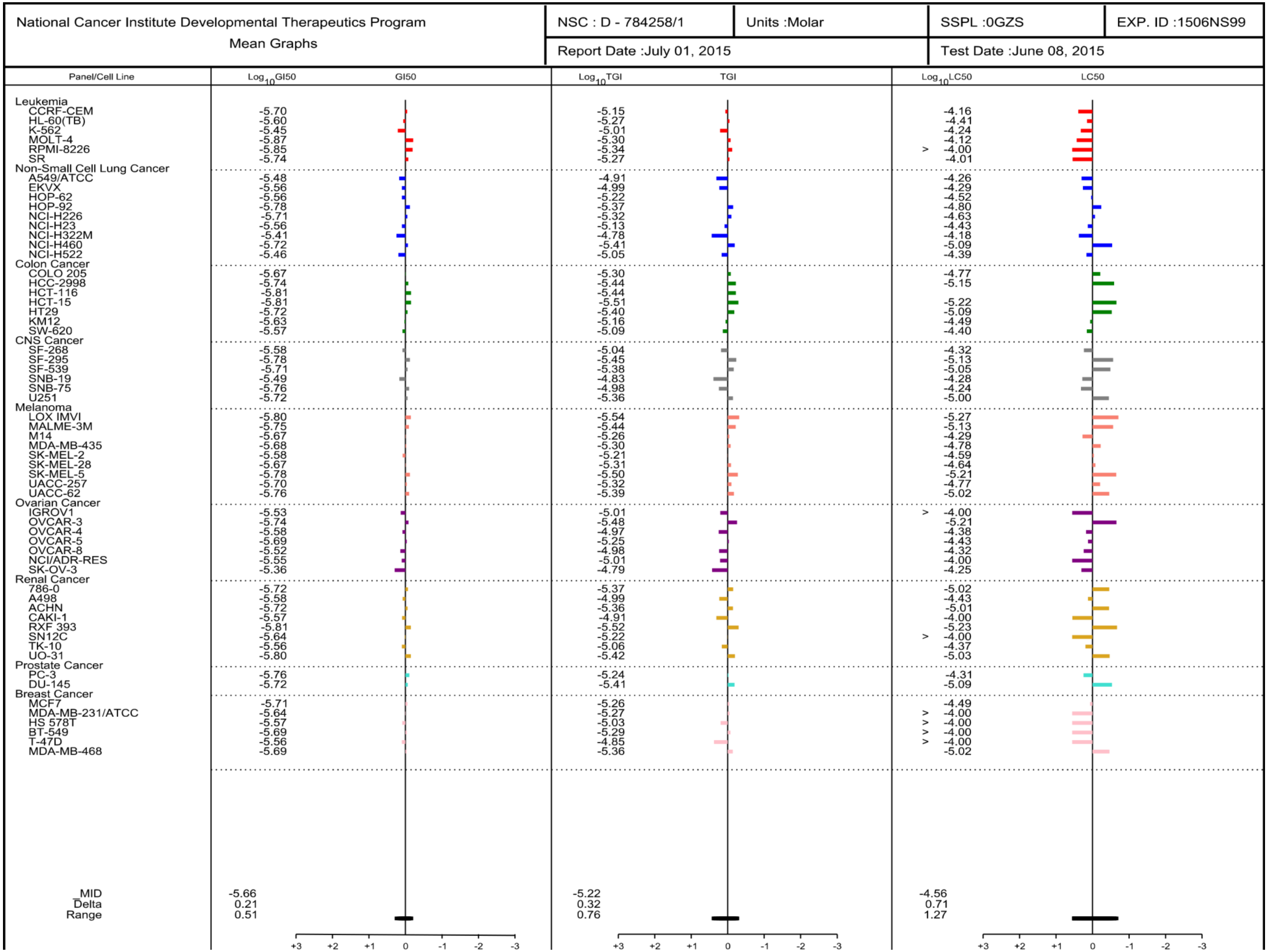


**Figure S17B.** $GI_{50}$ (50% Growth Inhibition), TGI (Total Growth Inhibition) and $LC_{50}$ (50% Lethal Concentration) mean graphs obtained for compound **4** (NSC 784258) tested at five concentrations (0.01, 0.1, 1, 10, 100 µM) against the NCI-60 human cancer cell lines.

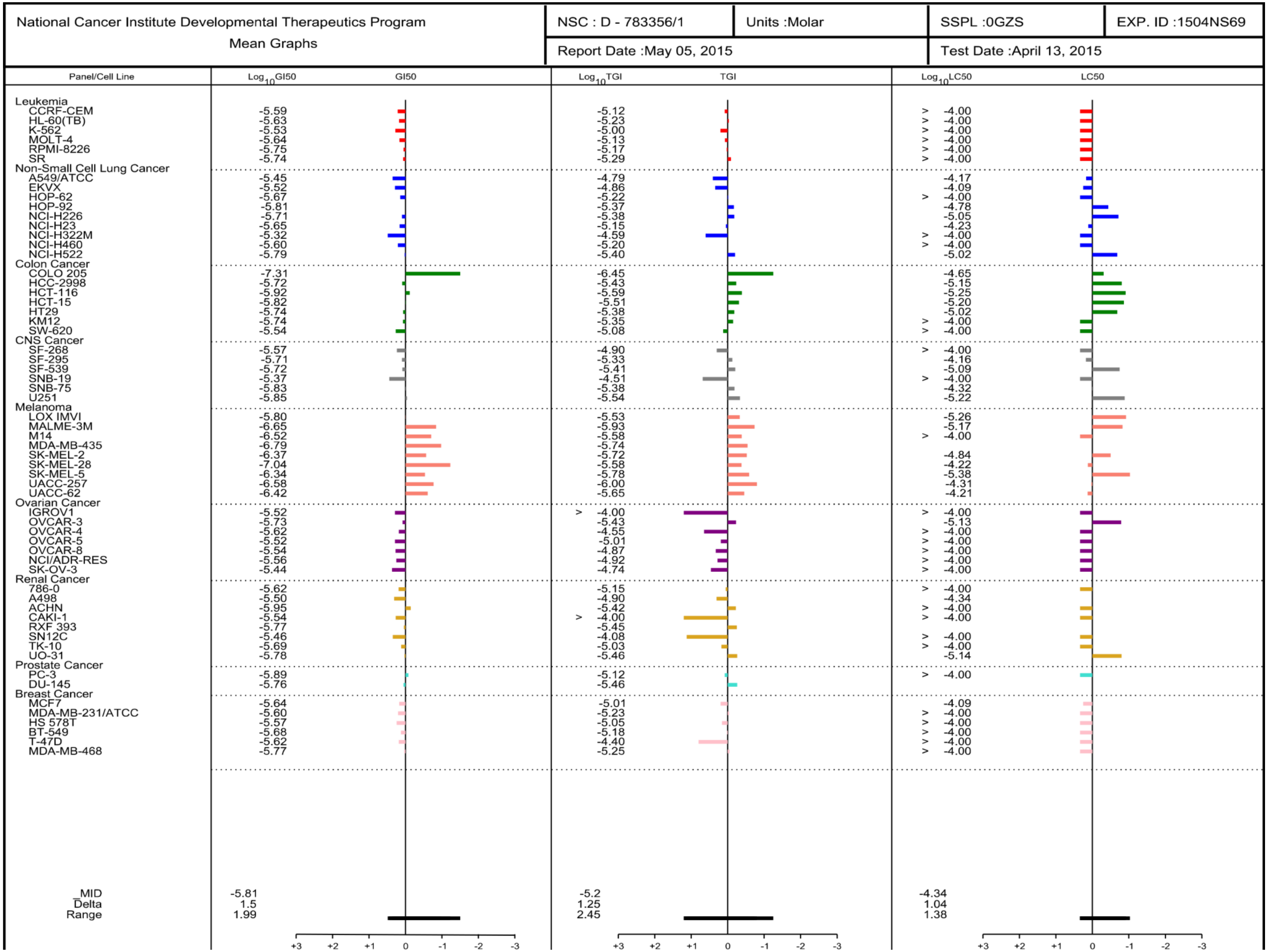


**Figure S18B.** $GI_{50}$ (50% Growth Inhibition), TGI (Total Growth Inhibition) and $LC_{50}$ (50% Lethal Concentration) mean graphs obtained for compound **5** (NSC 783356) tested at five concentrations (0.01, 0.1, 1, 10, 100 μM) against the NCI-60 human cancer cell lines.

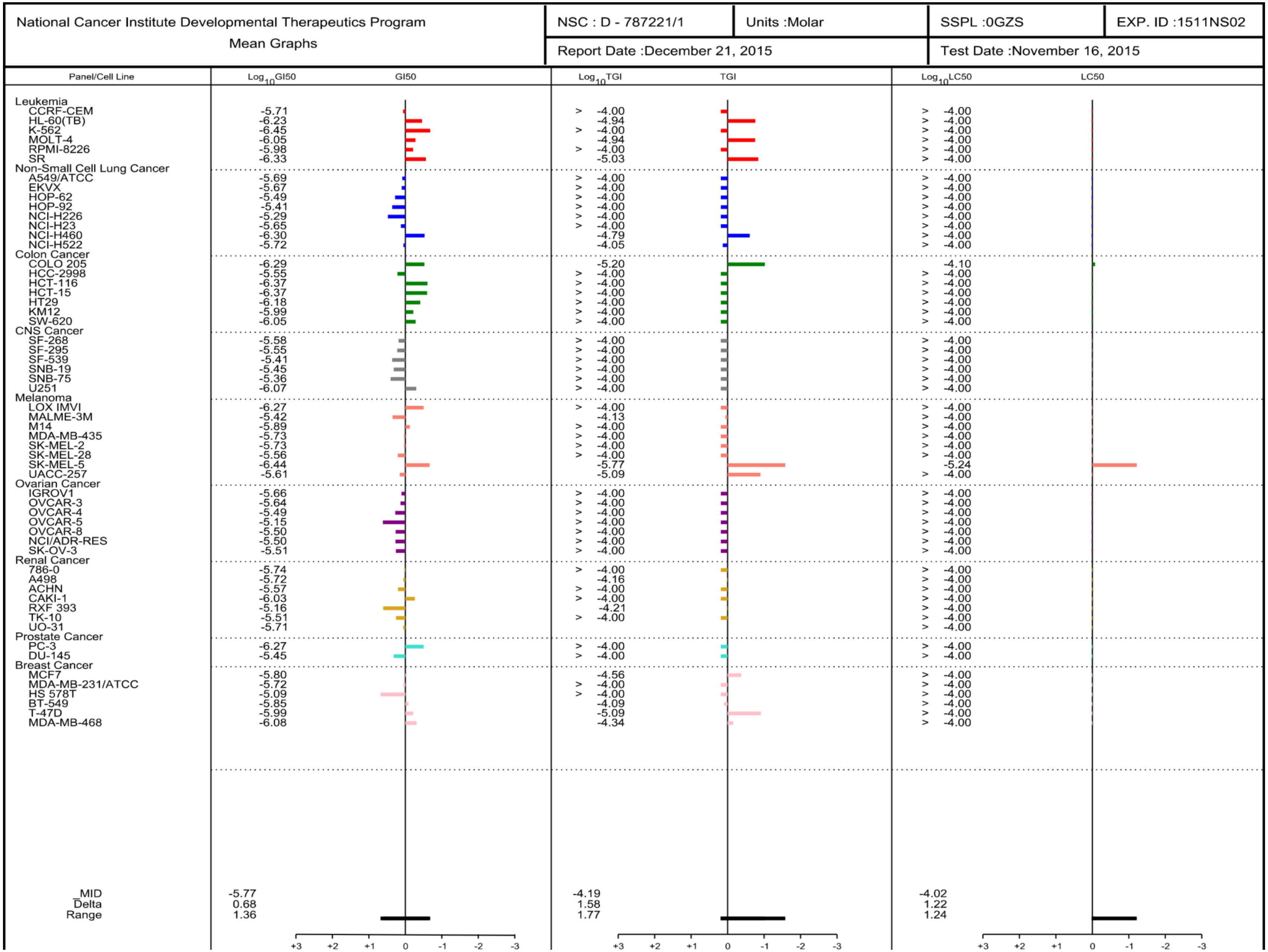


**Figure S19B.** $GI_{50}$ (50% Growth Inhibition), TGI (Total Growth Inhibition) and $LC_{50}$ (50% Lethal Concentration) mean graphs obtained for compound **6** (NSC 787221) tested at five concentrations (0.01, 0.1, 1, 10, 100 μM) against the NCI-60 human cancer cell lines.

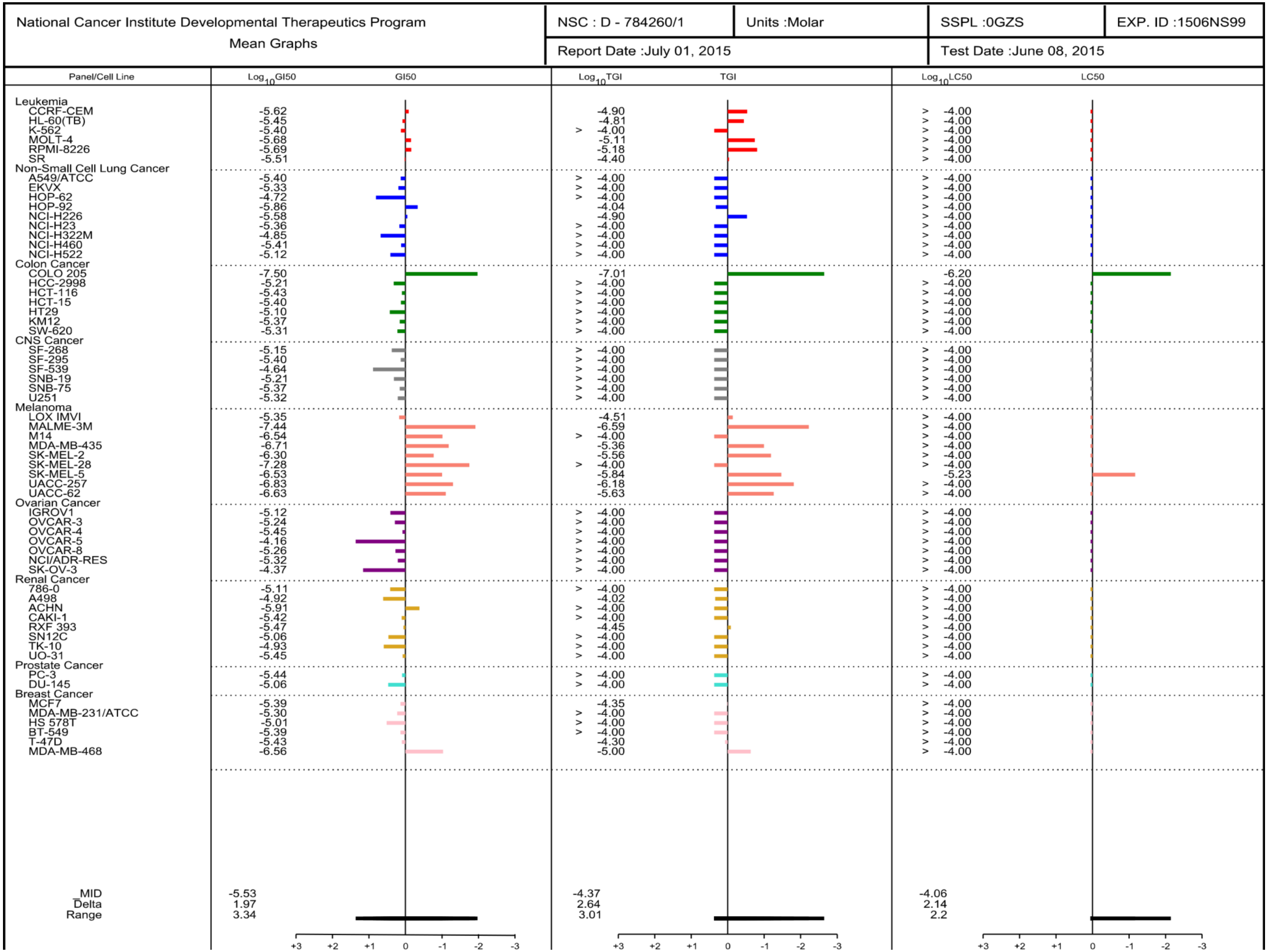


**Figure S20B.** $GI_{50}$ (50% Growth Inhibition), TGI (Total Growth Inhibition) and $LC_{50}$ (50% Lethal Concentration) mean graphs obtained for compound **12** (NSC 784260) tested at five concentrations (0.01, 0.1, 1, 10, 100 µM) against the NCI-60 human cancer cell lines.

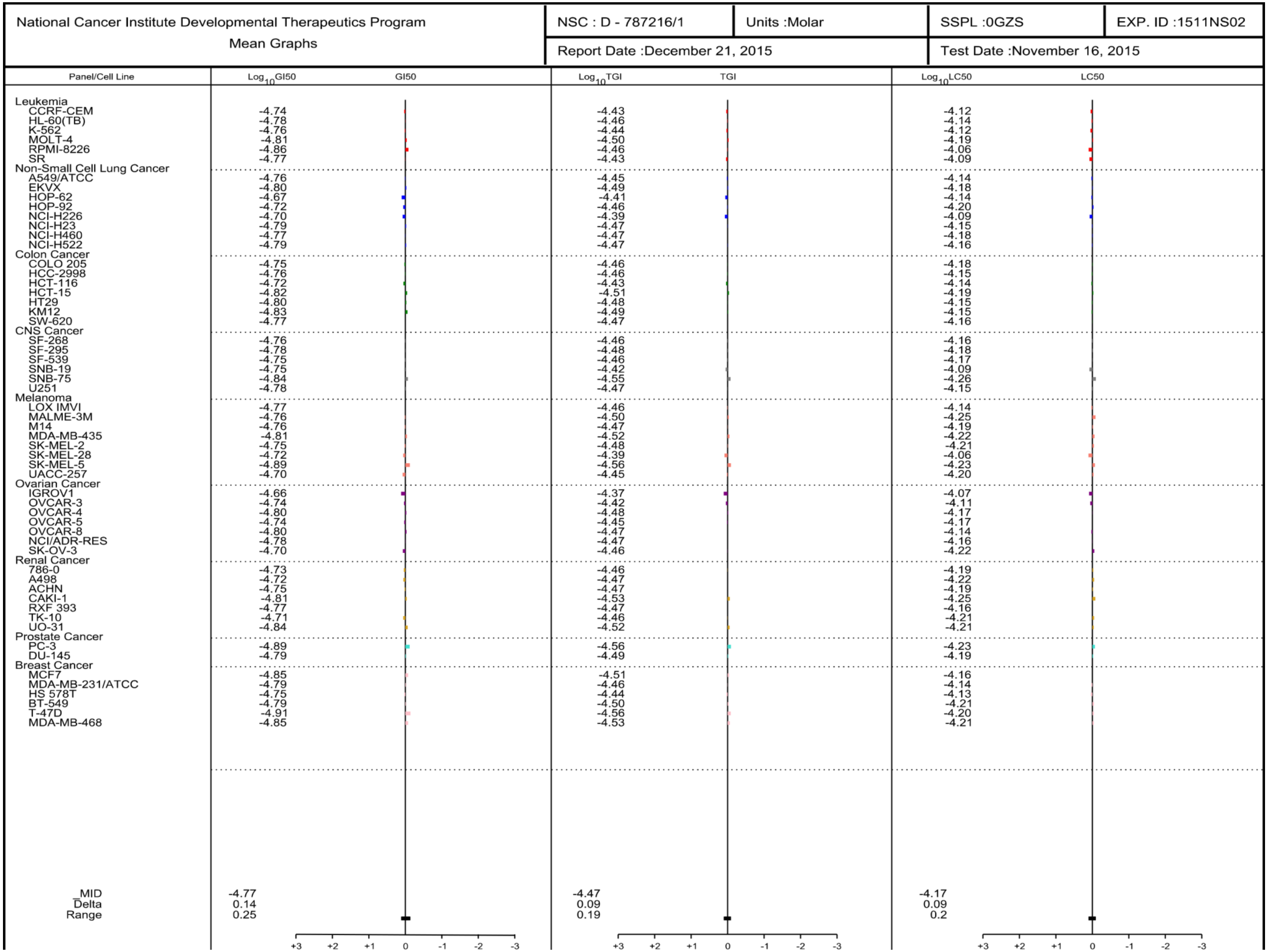


**Figure S21B.** $GI_{50}$ (50% Growth Inhibition), TGI (Total Growth Inhibition) and $LC_{50}$ (50% Lethal Concentration) mean graphs obtained for compound **14** (NSC 787216) tested at five concentrations (0.01, 0.1, 1, 10, 100 µM) against the NCI-60 human cancer cell lines.

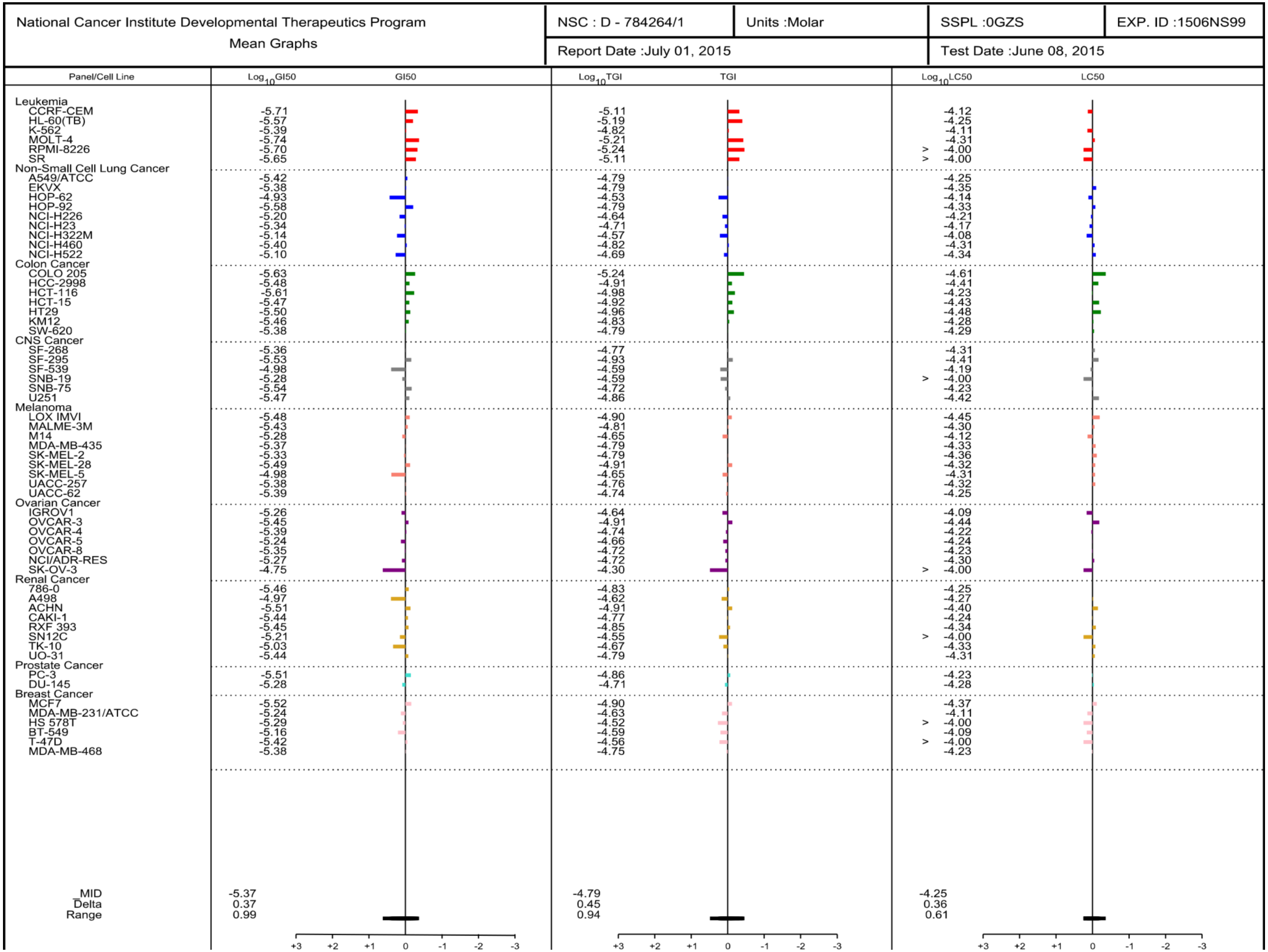


**Figure S22B.** $GI_{50}$ (50% Growth Inhibition), TGI (Total Growth Inhibition) and $LC_{50}$ (50% Lethal Concentration) mean graphs obtained for compound **16** (NSC 784264) tested at five concentrations (0.01, 0.1, 1, 10, 100 µM) against the NCI-60 human cancer cell lines.

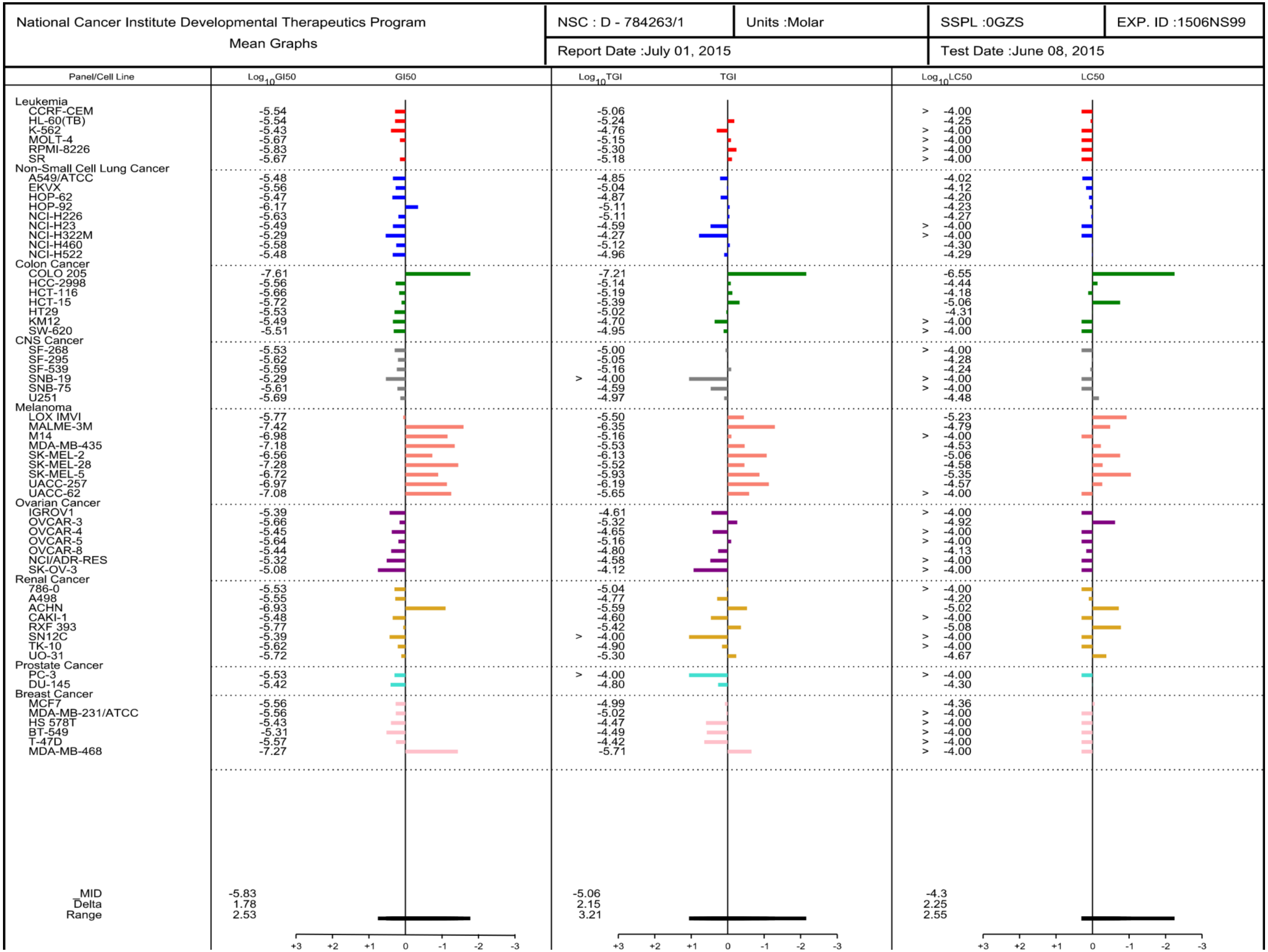


**Figure S23B.** $GI_{50}$ (50% Growth Inhibition), TGI (Total Growth Inhibition) and $LC_{50}$ (50% Lethal Concentration) mean graphs obtained for compound **17** (NSC 784263) tested at five concentrations (0.01, 0.1, 1, 10, 100 µM) against the NCI-60 human cancer cell lines.

| NSC 706829 | NSC 763760 | NSC 761431 | 5 | 12 | 17 |
|---|---|---|---|---|---|

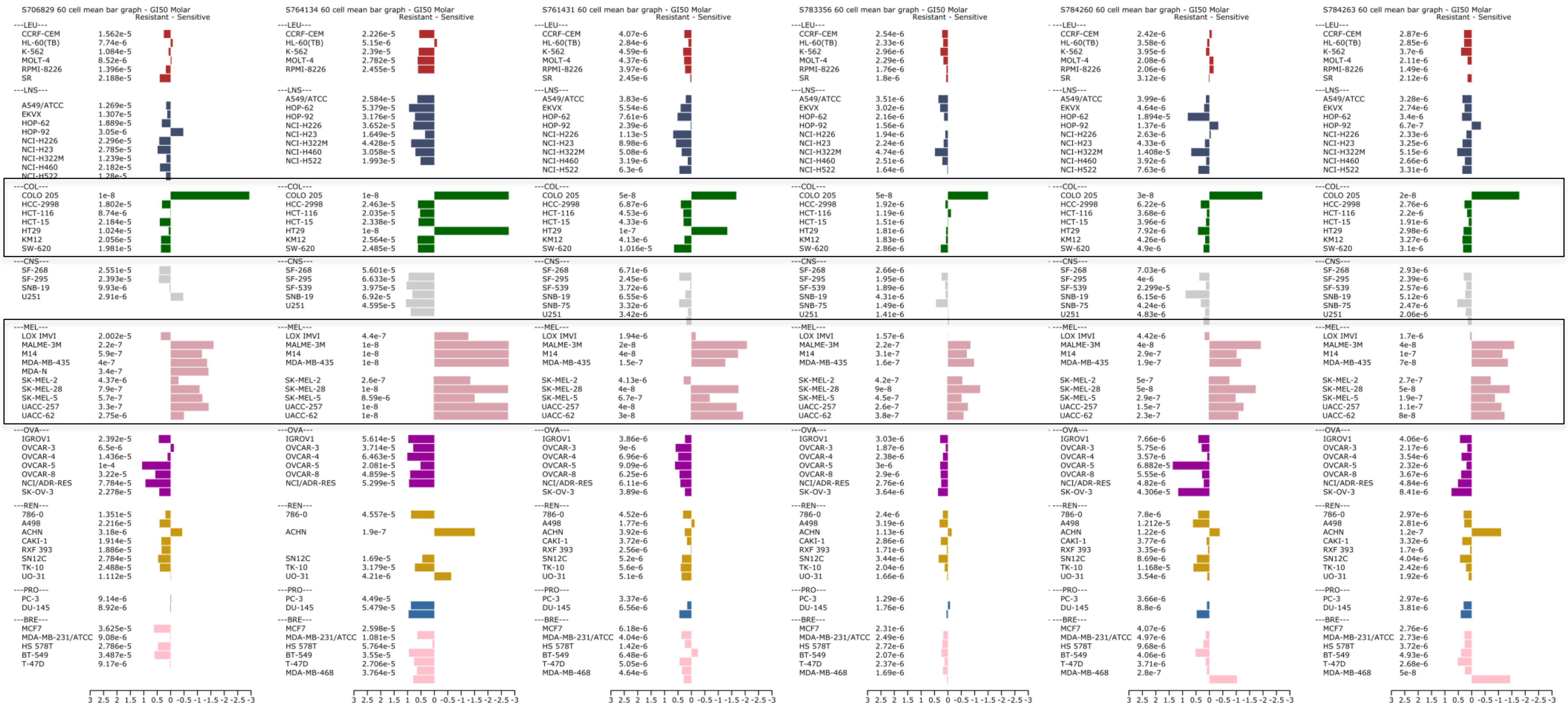


**Figure S24.** Comparison of the NCI-60 mean bar graphs of the three top-ranking drugs identified by CellMiner™ (NSC 706829, NSC 763760 and NSC 761431) and the mean graphs of our three selective MEA congeners (**5**, **12** and **17**).
